# Accelerating Proton Exchange in 1,8-Bis(dialkylamino)naphthalene Proton Sponges through Intramolecular Catalysis for CEST MRI

**DOI:** 10.1021/jacs.6c06687

**Published:** 2026-05-25

**Authors:** Mai T. Huynh, Madalina Ranga, Sara Chirayil, Janaka Wansapura, Elena Vinogradov, Xiaodong Wen, Mariangela Boccalon, Attila Benyei, James Ratnakar, Zsolt Baranyai, Zoltan Kovacs

**Affiliations:** † Advanced Imaging Research Center, 12334 University of Texas Southwestern Medical Center, 5323 Harry Hines Boulevard, Dallas, Texas 75390, United States; ‡ CRB Trieste, Bracco Imaging SpA, S. S. 14 km 163,5 AREA Science Park, Basovizza, 34149 Trieste, Italy; § Department of Radiology, UTSW Medical Center, Dallas, Texas 75390, United States; ∥ Department of Physical Chemistry, University of Debrecen, Egyetem tér 1, H-4032 Debrecen, Hungary

## Abstract

Chemical exchange saturation transfer (CEST) MRI generates contrast by transferring saturation from exchangeable protons to bulk water. In diamagnetic agents, labile −NH/–OH protons typically resonate close to water (1–5 ppm), severely limiting selectivity. Thus, larger chemical shift offsets are highly desirable for in vivo applications. Efficient CEST at clinical field strengths also requires exchange rates within a narrow kinetic window. Tetraalkyl 1,8-diaminonaphthalenes (proton sponges) are attractive scaffolds for diaCEST agents because the labile proton locked in a strong hydrogen bond between the two nitrogens in the monoprotonated forms can exhibit extraordinarily large downfield chemical shift offsets from water. However, these compounds do not have CEST because the strong H-bond also results in very slow exchange with the bulk. Here, we report the design, synthesis, and evaluation of a new tetrasubstituted 1,8-diaminonaphthalene derivative in which proton exchange is accelerated by intramolecular catalysis. Detailed structural, thermodynamic, and kinetic studies of 1,8-diaminonaphthalene tetraacetic acid (DANTA) reveal that the carboxylate groups participate in intramolecular hydrogen-bonding interactions with the central N···H^+^···N bridge, markedly accelerating the proton exchange rate to 492 s^–1^ at 37 °C, which is within the optimal kinetic regime for CEST at 3 T. Importantly, the carboxylate substituents do not affect the chemical shift offset of the exchanging proton, which remains substantially deshielded and highly downfield shifted at 12.5 ppm from water. In vivo MRI studies further demonstrate that DANTA generates exquisite CEST contrast in the kidneys of mice at 3 T using clinically permissible saturation RF field strengths.

## Graphical abstract

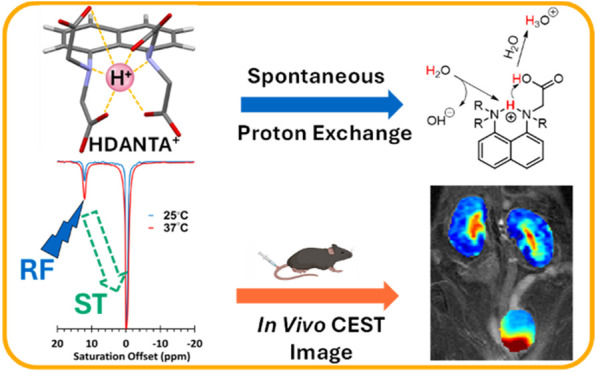

## Introduction

Achieving simultaneous control over proton exchange kinetics and chemical shift remains a fundamental challenge in the design of chemical exchange saturation transfer (CEST) MRI contrast agents. CEST MRI is a powerful imaging modality that allows the noninvasive detection of biomolecules and physiological biomarkers, offering specificity at the molecular level that cannot be achieved with conventional *T*_1_/*T*_2_-weighted MRI. CEST contrast is generated by applying a frequency-selective radiofrequency saturation pulse (*B*_1_) to a small pool of exchangeable protons that is in chemical exchange with the bulk water pool. Subsequent chemical exchange transfers the saturated magnetization to bulk water, reducing the water signal and generating negative contrast. CEST requires that the proton exchange rate remain in the slow-to-intermediate regime on the NMR time scale (*k*_ex_ ≤ Δ*ω*, where *k*_ex_ is the pseudo first order rate constant for the forward exchange and Δ*ω* is the chemical shift separation between the exchanging pools in Hz), so that their resonances remain spectrally distinguishable.^1–7^ In vivo CEST measurements are complicated by tissue magnetization transfer (MT) effects originating from dipolar interactions between water protons and endogenous macromolecules.^8^ Paramagnetic lanthanide and transition-metal complexes have therefore been explored as paraCEST agents because hyperfine interactions with unpaired electrons can shift exchangeable proton resonances far from the water signal, often outside the MT window (±100 ppm).^1,9,10^ However, their *in vivo* application remains limited by nonoptimal exchange kinetics and exchange-induced broadening of the water signal.^11^ These limitations motivate the development of metal-free diamagnetic CEST (diaCEST) agents,^12^ especially the ones that combine large chemical shift offsets from water (Δ*ω*) with exchange rates suitable for efficient saturation transfer.

Most diaCEST agents contain exchangeable −NH or −OH protons that resonate within 1–5 ppm from water, overlapping with CEST signals originating from endogenous molecules. In addition, residual asymmetry of tissue MT near the water signal, which is due to broad MT effects that are not centered on the water frequency, interferes with the CEST contrast computation. This effect diminishes around 10 ppm from water but it can extend to larger offsets when high B_1_ saturation rf field strength is used.^13^ Larger chemical shift offsets are therefore highly desirable because they improve spectral specificity, reduce direct water saturation, and minimize MT interference.^14^ Higher B_1_ strength is needed for efficient saturation of faster exchange protons. Although a large Δ*ω* may allow a wide kinetic window in which efficient CEST can occur even at clinical magnetic field strength (≈3 T), the clinically admissible *B*_1_ strength limits the proton exchange rates on the order of a few hundred s^–1^. To adhere to safety limits for specific absorption rates (SAR), most human studies use saturation strengths less than 4 μT (most commonly 2 μT). Higher *B*_1_ strengths could be applied for shorter saturation times, as long as the safety limits for specific absorption rate (SAR) are met.^7^ Elegant work by McMahon and co-workers demonstrated that strong intramolecular hydrogen bonding in salicylates, anthranilates, substituted imidazoles, and related systems can generate highly downfield-shifted labile proton resonances (Table S1).^14^ Although most of these compounds exhibit nonoptimal exchange kinetics that limit their use at clinically relevant field (3 T) and acceptable *B*_1_ saturation strengths, salicylate has emerged as the benchmark diaCEST agent.^15,16^

Here we introduce a new class of highly shifted diaCEST agents based on the 1,8-bis­(dialkylamino)­naphthalene scaffold (Figure 1), commonly known as proton sponges. The two amino groups in 1,8 also known as *peri* position in these compounds are closer to each other than *ortho* substituents. The attachment of the first proton results in the formation of a high stability intramolecular N···H^+^···N hydrogen bridge and the high basicity of these compounds is attributed to the relief of the severe lone-pair repulsion between the two N atoms in the monoprotonated species. The proton participating in this hydrogen bond has an extraordinarily downfield shifted NMR signal, often 16 ppm from water, significantly larger than those typically observed for conventional diaCEST agents.^17^ Despite this favorably large shift, protonated proton sponges cannot be used as CEST agents because the exchange of the highly shifted proton is exceedingly slow. In this work, we modify the structure to include a functional group that accelerates proton exchange via intramolecular catalysis without affecting the large Δ*ω*. This approach establishes proton sponges as a versatile platform for the design of metal-free diaCEST probes with unusually large chemical shift offsets, bridging the gap between conventional diaCEST agents and paraCEST systems.

**Figure 1:**
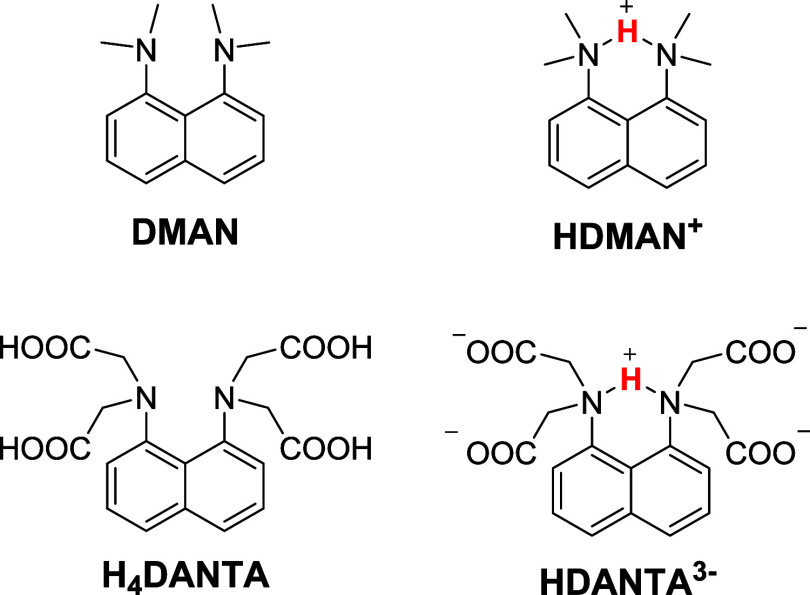
Chemical structure of 1,8-bis­(dimethylamino)­naphthalene (DMAN) and 1,8-bis­(diamino)­naphthalene tetraacetic acid (H_4_DANTA), and their monoprotonated species that exist between pH 6 and 9 in aqueous media. The highly shifted exchanging proton is highlighted in red. The protonation constants are shown in Table 1.

## Results and Discussion

### Probe Design and Synthesis

Preliminary experiments and literature data^18^ clearly show that proton exchange in tetraalkyl-substituted 1,8-diaminonaphthalenes (Figure 1) is too slow to generate efficient CEST contrast. Although the large chemical shift separation of the N···H^+^···N proton would allow exchange rates beyond 1000 s^–1^ even at clinical magnetic field strengths, practical applications require substantially lower exchange rates to maintain reasonable *B*_1_ saturation strength. According to the relationship *k*_ex_ ≈ 2π*B*_1_,^19–21^ optimal CEST performance at *B*_1_ < 3 μT corresponds to exchange rates of less 800 s^–1^. Therefore, the proton exchange rate of tetraalkyl diaminonaphthalenes must be increased by several orders of magnitude to reach this regime.

Because proton exchange in these compounds is base-catalyzed, based on our previous experience with paraCEST agents and Gd­(III) chelates, we hypothesized that exchange of the intramolecularly hydrogen-bonded proton could be accelerated through intramolecular catalysis.^22–25^ In this design, a basic functional group could transiently accept the proton from the N···H^+^···N bridge and subsequently transfer it to bulk water, thereby facilitating exchange. To implement this strategy, we designed a derivative of 1,8-diaminonaphthalene bearing four acetate side arms capable of acting as internal proton acceptors. The resulting ligand, DANTA (Figure 1), was synthesized as outlined in Scheme 1. The product was obtained in good yield and fully characterized by high-resolution NMR spectroscopy, LC–MS, and elemental analysis. It is worth noting that the slowly exchanging proton is clearly visible as a broad singlet at around 17.2 ppm in water (Figure 2).

**Scheme 1:**
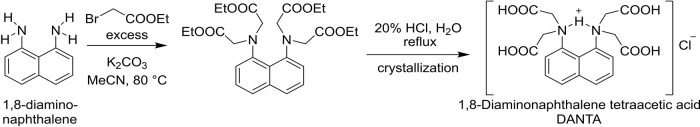
Synthesis of 1,8-Diaminonaphthalene Tetraacetic Acid (DANTA)

**Figure 2:**
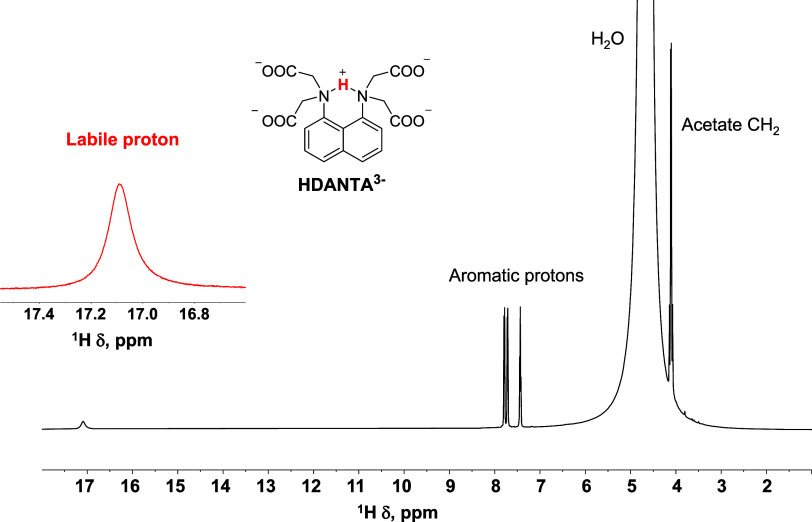
^1^H NMR spectrum of DANTA at pH = 7.07 and 293 K in 0.15 M NaCl aqueous solution. ([DANTA] = 0.012 M, 9.4 T, H_2_O).

### Acid–Base and Complexation Properties of DANTA

As the basicity of the carboxylates obviously plays an important role in the intramolecular catalysis of the proton exchange, the protonation constants of DANTA, as well as the thermodynamic stability of its complexes formed with biologically relevant metal ions were determined by pH-potentiometry, ^1^H NMR spectroscopy (Figures 2 and S3–S5) and spectrophotometry (Figure S6). The data are summarized in Tables 1, S2 and S3. Equations and expressions used for the characterization of the acid–base and the complexation properties of DANTA are reported in the ESI.

**Table 1:** Protonation Constants (log*K*_i_^H^) of the DANTA and DMAN, Stability Constant (log*K*_ML_) and Protonation Constant (log*K*_MHL_) of Ca­(II), Zn­(II) and Cu­(II) Complexes with DANTA (25 °C)

	DANTA			DMAN^18^
method	pH-pot.	UV-tit.	^1^H NMR	photometry
I	0.15 M NaCl			0.1 M NaCl
log*K*_1_^H^	10.75 (2)	10.70 (3)	10.65 (3)	12.1
log*K*_2_^H^	4.84 (5)	4.68 (5)	4.68 (2)	
log*K*_3_^H^	4.00 (5)	4.06 (5)	3.95 (5)	
log*K*_4_^H^	3.15 (5)	3.31 (9)	2.91 (2)	
log*K*_5_^H^	1.70 (6)			

[M(DANTA)]^2–^				
	log*K*_ML_		log*K*_MHL_	
I	0.15 M NaCl			
Ca^2+^	5.68 (6)		7.19 (8)	
Zn^2+^	13.33 (2)		5.19 (8)	
Cu^2+^	17.72 (2)		2.95 (3)	

NMR titrations show that the first two protonation steps correspond to the amine nitrogens of the 1,8-diaminonaphthalene scaffold, whereas the remaining protonation constants arise from the carboxylate groups. The overall protonation pattern and total basicity (Σlog*K*_i_^H^, Table S2) are consistent with those expected for diamino tetraacetate ligands and are comparable to EDTA and related systems.^26^ In contrast, the stability constants of the Ca­(II), Zn­(II), and Cu­(II) complexes of DANTA are generally 1–5 orders of magnitude lower than those of structurally similar polyamino polycarboxylate ligands (Tables 1 and S3). This reduced stability likely reflects the high rigidity of the 1,8-diaminonaphthalene backbone, which provides a coordination cavity that is less flexible and not ideally matched to these metal ions.

### X-ray Structure of the Monoprotonated HDANTA^3–^

To evaluate potential interactions between the acetate side arms and the central N···H^+^···N bridge, the structure of the monoprotonated species was examined by single-crystal X-ray diffraction.

The structures obtained from the X-ray diffraction data, selected bond distances and the hydrogen bond parameters of the monoprotonated HDANTA^3–^ are shown in Figures 3, S36 and S37, Tables S5 and S6, respectively. Protonation of 1,8-diaminonaphthalene derivatives is known to induce pronounced structural changes, including shortening of the N···N distance and increasing the planarity of the naphthalene framework. Consistent with these trends, the crystal structure of HDANTA^3–^ reveals a protonated N···H^+^···N bridge characteristic of proton sponge systems. The asymmetric unit contains two crystallographically distinct conformers (δ and λ isomer; Figure 3). These two isomers differ in the orientation of the acetate arms: in the δ isomer, the acetate arms are oriented clockwise while in the λ isomer, they are oriented counterclockwise around the naphthalene core. Comparison of the geometric parameters of the N···H^+^···N bridge in HDANTA^3–^ with those of the monoprotonated bis­(dimethylamino)­naphthalene (HDMAN^+^) is summarized in Table 2.

**Figure 3:**
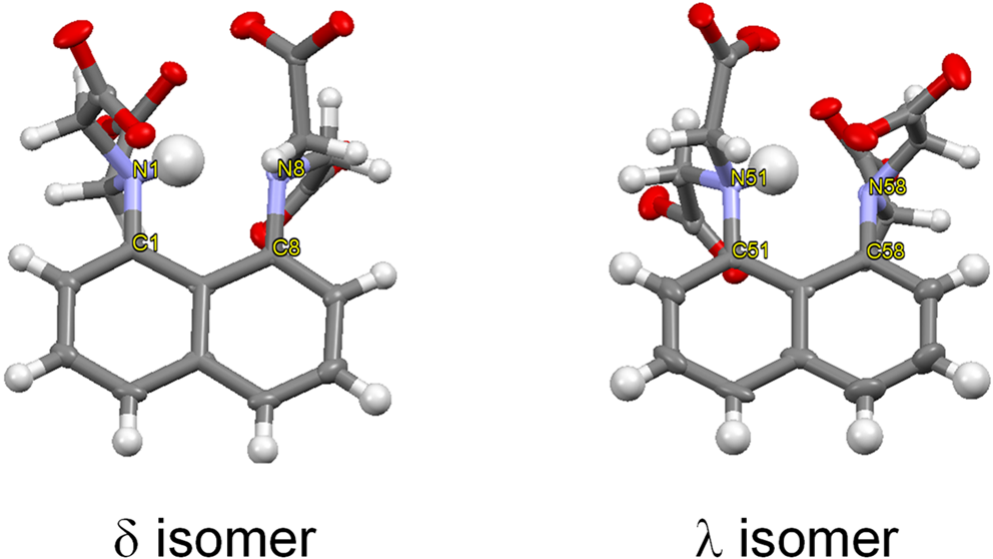
ORTEP view monoprotonated HDANTA^3–^ at 50% probability level. Solvent water and ethanol molecules as well as sodium counterions are omitted for clarity.

**Table 2:** Geometry Parameters of N···H^+^···N Hydrogen Bridge in the Mono-Protonated HDANTA^3–^ and HDMAN^+^

	distances (Å)					angles (°)	
compound	N···N	N–H	N···H	N–C_N_	D*N* ^a^	N···H–N	ring twist^b^
HDANTA^3–^ δ isomer	2.645	0.87(2)	1.84(4)	1.460(5) (N–C_N_) 1.479(5) (HN-C_N_)	0.245	153(8)	4.9
HDANTA^3–^ λ isomer	2.619	0.87(2)	1.85(5)	1.455(5) (N–C_N_) 1.466(5) (HN-C_N_)	0.196	146(8)	4.1
HDMAN^+^ ^27^	2.621	1.057	1.628	1.462 (N–C_N_) 1.475 (HN-C_N_)	0.025	154	1.0

a The average distance of the N atoms from the mean ring plane.

b The twist angle of the naphthalene ring.

The N···N distances in both HDANTA^3–^ conformers are similar to those observed in HDMAN^+^, whereas the naphthalene framework exhibits a significantly larger twist. In addition, the nitrogen atoms deviate more strongly from the mean plane of the aromatic ring system than in HDMAN^+^, reflecting structural distortion introduced by the acetate substituents. The X-ray structures indicate weak interactions between the carboxylate oxygens and the bridging proton (for the two closest oxygens, δ isomer: H···O^–^ = 2.57 and 2.76 Å; λ isomer: H···O^–^ = 2.48 and 2.90 Å). The corresponding N–H···O angles (80–100°) suggest suboptimal hydrogen-bond geometries and therefore relatively weak interactions. The larger asymmetry of the N···H^+^···N bridge in DANTA arises from the intramolecular hydrogen-bond network formed with the carboxylate groups. Importantly, although these interactions with the carboxylates weaken the N···H^+^···N bridge, they do not disrupt it. From a probe design perspective, this balance is advantageous: stronger interactions with the carboxylates would destabilize the central hydrogen bond and reduce the chemical shift separation, whereas weaker interactions would have little accelerating effect on the proton exchange rate. The observed structures therefore support our proposed mechanism of intramolecularly assisted proton exchange. Finally, interactions between Na^+^ counterions and the carboxylate groups in the crystal packing (Figure S37) further contribute to the asymmetry of the N···H^+^···N bridge in the solid state.

### Proton Exchange Kinetics

The rate of exchange of the labile proton (*k*_ex_) directly determines the magnitude of saturation transfer. Tetraalkyl-substituted 1,8-diaminonaphthalenes such as HDMAN^+^ exhibit proton exchange rates that are five to 7 orders of magnitude slower than those of conventional ammonium ions, which typically undergo diffusion-controlled proton transfer.^18,28^ According to the general proton-transfer theory, exchange occurs via the formation of a hydrogen-bonded adduct between the proton donor and acceptor. Eigen’s model predicts that the rate of proton transfer is proportional to the difference in basicity between donor and acceptor (*k*_ex_ ∼ Δp*K* = p*K*_donor_ – p*K*_acceptor_).^28^ However, the proton transfer rates of tetraalkyl-substituted 1,8-diaminonaphthalenes to OH^–^ are several orders of magnitude slower than the predicted based on the basicities of the deprotonated compounds and hydroxide ion.^18^ This deviation from a diffusion controlled process arises from the strong intramolecular hydrogen bond present in the monoprotonated species. The X-ray structure of HDMAN^+^ reveals a short, nearly symmetrical N···H^+^···N hydrogen bond (Table 2).^27^ Consistent with this structure, our ^1^H NMR studies confirmed the extraordinarily slow exchange between the N···H^+^···N proton of HDMAN^+^ and water at pH 7.05 over the temperature range 293–343 K (Figures S20–S28). To investigate the influence of hydrogen bonding between the N···H^+^···N proton and the carboxylate groups of the pendant arms on the proton exchange, the rate of proton transfer between HDANTA^3–^ and water was studied by ^1^H NMR spectroscopy over the pH range 4.5–9.5 and temperatures from 273 to 298 K. Representative spectra recorded at different pH values and temperatures are shown in Figures 4, S10 and S11. As temperature increases and pH decreases, the N···H^+^···N resonance broadens, indicating acceleration of the chemical exchange process (Scheme 2).

**Scheme 2:**
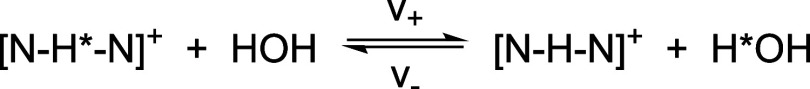
Chemical Exchange between Monoprotonated HDANTA^+^ and Water, Where * Denotes the Exchanging Proton and *v*_+_ and *v*_–_ are the Forward and Backward Rates (*v*_±_ = *k*_±_[N···H^+^···N] × 2­[H_2_O])

Since the system is at equilibrium (*K* = 1, Δ*G* = 0 and Δ_r_ *G* ^0^= 0 = −*RT*ln*K*), the rates of the forward and backward exchange reactions are identical (*v*_+_ = *v*_–_) and component concentrations do not change at constant pH.

The parameters governing the proton exchange rate of HDANTA^+^ were determined using variable-temperature and variable-pH selective magnetization transfer experiments employing a DANTE (Delays Alternating with Nutations for Tailored Excitation) pulse train with selective 180° irradiation at either the water or labile-proton resonance.^29^ Representative DANTE experiments are shown in Figures 4, S12 and S14. Signal integrals as a function of delay time, together with fitted curves for a two-site exchange model, are presented for pH = 7.07 at 273 K in Figures 4, S13 and S15. The longitudinal relaxation times (*T*_1_^H2O^ and *T*_1_^NHN^) and the forward and reverse rate constants (*k*_+_ and *k*_–_) were obtained from the simultaneous fitting of the integrals for water and the N···H^+^···N proton signal. Fitting was performed using eqs S17 and S18, and the resulting parameters are summarized in Figures 4, S16–S17. Between pH 6.5 and 9.5, *k*_+_ and *k*_–_ are essentially pH-independent, indicating dominant spontaneous exchange (*k*_0_, Figure 4) between the N···H^+^···N proton of HDANTA^3–^ and water. The relatively rapid spontaneous exchange is attributed to intramolecular catalysis arising from hydrogen bonding between the carboxylate groups and the N···H^+^···N proton, which was also supported by X-ray diffraction data (Figures 3 and S36, Table S6). At lower pH, *k*_+_ and *k*_–_ rate constants increase due to proton-assisted exchange involving formation of the diprotonated species H_2_DANTA^2–^ (characterized with the equilibrium constant *K*_2_^H^, Table 1) followed by rapid spontaneous proton exchange with water (*k*_H2L_). The proposed mechanism is summarized in Figure 4. The temperature dependence of the spontaneous (*k*_0_) and proton-assisted rate constants (*k*_1_ = *k*_H2L_ × *K*_2_^H^) is shown in Figure S18, and experimental details are provided in the Supporting Information. Eyring plots for *k*_0_ and *k*_1_ are shown in Figure S19, and the corresponding activation parameters are summarized in Table 3. Both the spontaneous (*k*_0_) and acid catalyzed (*k*_1_) processes exhibit similar activation enthalpies but markedly different entropies. The spontaneous exchange pathway shows a negative Δ*S* ^‡^, consistent with a highly organized transition state involving rearrangement of the hydrogen-bond network and hydration of the pendant arms. In contrast, the proton-assisted pathway displays a large positive Δ*S* ^‡^, likely reflecting a more open structure of the diprotonated species due to electrostatic repulsion between the protonated amines. Rearrangement in the transition state may involve dehydration of the carboxylate group, giving rise to the positive entropy term. Because of the large difference in activation entropy, the Δ*G*_298_^‡^ and Δ*G*_310_^‡^ free-energy barrier values for the spontaneous exchange is nearly twice that of the proton-assisted pathway. From the measured rate *k*_±_ constants, the *k*_ex_ pseudo-first-order rate constant can be calculated at 298 and 310 K as *k*_ex_^298^ = *k*_±_^298^ × 2­[H_2_O] = 208 s^–1^ and *k*_ex_^310^ = *k*_±_^310^ × 2­[H_2_O] = 492 s^–1^ (assuming a water proton concentration is twice of the water concentration, 111 M). Notably, the value of *k*_ex_ at 310 K (37 °C) is near optimal for *in vivo* CEST applications.

**Figure 4:**
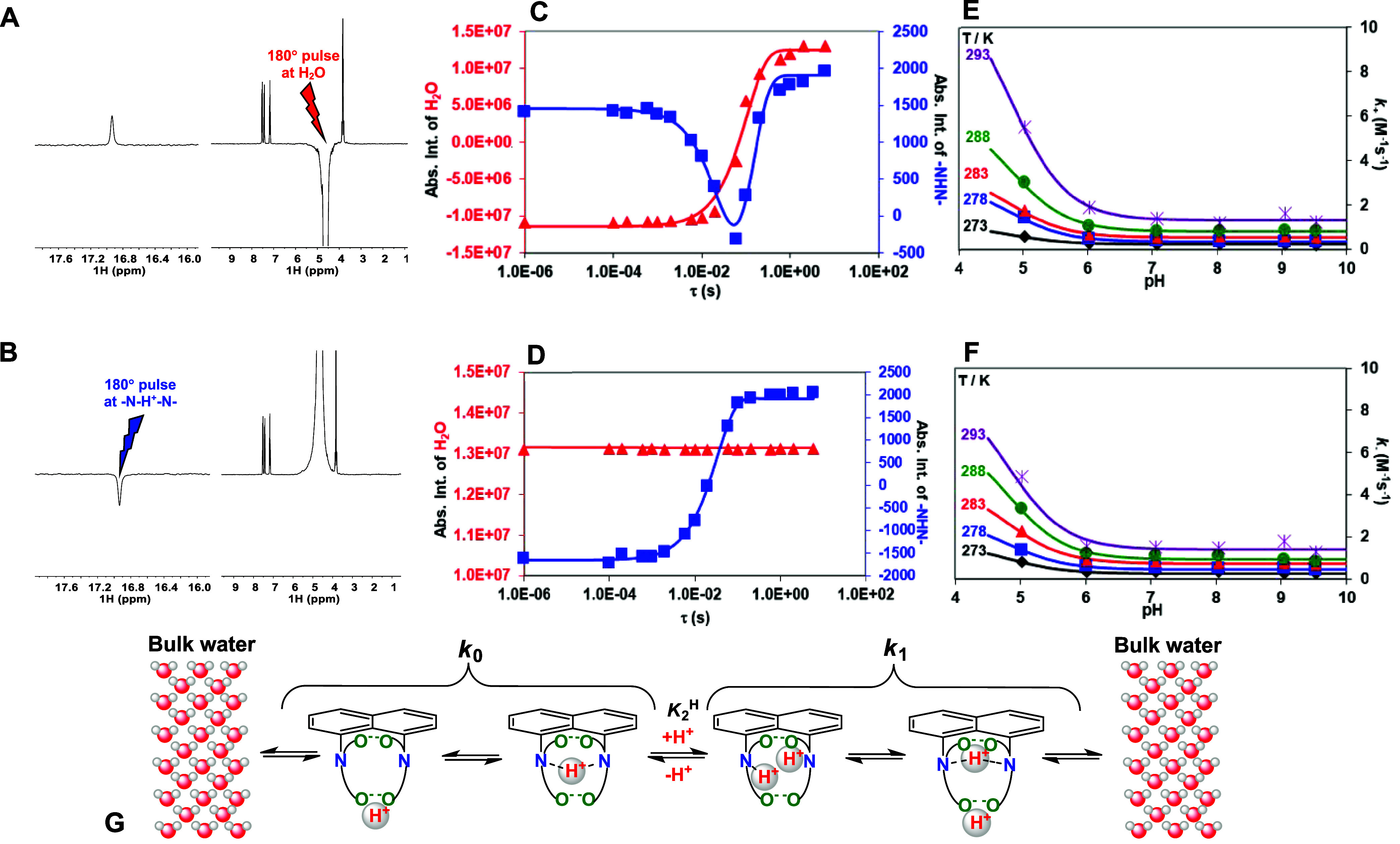
^1^H NMR spectra of HDANTA^+^ with the selective excitation of the H_2_O (A) and −N-H^+^-N- (B) resonances at 273 K and pH = 7.07 (τ = 10 μs). Absolute intensity of the N···H^+^···N (blue diamond) and H_2_O (red solid triangle) proton signals obtained by the selective excitation of the H_2_O (C) and N···H^+^···N (D) resonances as a function of τ delay time between the 180° (DANTE) and 90° pulses at pH = 7.07 and 273 K. The *k*_+_ (E) and *k*_–_ (F) rate constants characterizing the exchange between H_2_O and N···H^+^···N protons in HDANTA^3–^ solution (*v*_±_ = *k*_±_[N···H^+^···N] × 2­[H_2_O]). Symbols and solid lines represent experimental and calculated rate constants, respectively. Proposed mechanism (G) of the exchange reactions between the N···H^+^···N proton of HDANTA^3–^ and H_2_O ([DANTA] = 0.012 M, 9.4 T, H_2_O, 0.15 M NaCl).

**Table 3:** Activation Parameters of the Spontaneous (*k*_0_) and Acid Catalyzed (*k*_1_) Proton Exchange Processes the Rate Constants for the Proton Exchange Reactions Shown in Scheme 2 and Figure 4 in the Forward and Backward Directions (*k*_+_ and *k*_–_) and the Pseudo First Order Rate Constant (*k*_ex_) between N···H^+^···N Proton of HDANTA^3–^ and H_2_O

	*k*_0_	*k*_1_
Δ*H* ^‡^/kJ·mol^–1^	52 ± 4	65 ± 4
Δ*S* ^‡^/J·mol^–1^ K^–1^	–64 ± 10	88 ± 15
Δ*G*_298_^‡^/kJ·mol^–1^	71.4	38.6
Δ*G*_310_^‡^/kJ·mol^–1^	72.2	37.6
*k*_±_^298^/M^–1^ s^–1^ at pH = 7.4	**1.88**	
*k*_ex_^298^ = *k*_±_^298^ × 2[H_2_O]/s^–1^	**208**	
*k*_±_^310^/M^–1^ s^–1^ at pH = 7.4	**4.43**	
*k*_ex_^310^ = *k*_±_^310^ × 2[H_2_O]/s^–1^	**492**	

Among the commonly used diaCEST agents, the phenolate–OH proton of the salicylate has the largest chemical shift offset (Δ*ω* = 9.3 ppm, Table S1, Figures S29, S30 and S32). However, the exchange rate of this proton is significantly faster than that of the N···H^+^···N proton of HDANTA^3–^ (salicylate: *k*_ex_^298^ = 330 s^–1^; HDANTA^3–^: *k*_ex_^298^ = 208 s^–1^, pH = 7.4, 298 K, 0.15 M NaCl) due to the very fast, exclusively acid catalyzed proton exchange process of salicylate (*k*_1_ = (4.0 ± 0.4) × 10^7^ M^–2^s^–1^ at 298 K in 0.15 M NaCl) (Figures S29–S34 and eqs S23–S27). The rapid acid catalyzed proton exchange of the phenolate–OH proton in salicylate has been confirmed by CEST experiments previously.^15^

### Interaction with Albumin

Albumin has specific hydrophobic sites (Sudlow sites I and II) that can bind a wide variety of aromatic compounds including naphthalene derivatives.^30^ Therefore, the binding constant of [Ca­(DANTA)]^2–^ to human serum albumin (HSA) was determined with ultrafiltration of [Ca­(DANTA)]^2–^ – HSA mixtures followed by measuring the free [Ca­(DANTA)]^2–^ in the filtrate with capillary zone electrophoresis (Figures S8 and S9). The *K*_a_ binding constant of DANTA containing species ([DANTA]_free_, [Ca­(DANTA)] and [Ca­(HDANTA)]) to HSA was found to be weak, 158 ± 32 M^–1^. In comparison, liver specific gadolinium based agents such as Eovist^31^ and MultiHance^32^ have a binding constant of 772 and 490 M^–1^, respectively, which results in less than 10% protein bound fraction (Eovist: gadolinium ethoxybenzyl-diethylenetriaminepentaacetic acid; MultiHance: gadolinium benzyloxymethyl-diethylenetriaminepentaacetic acid). This weak albumin binding is not expected to significantly alter the biodistribution and clearance of DANTA.

### CEST Spectroscopy

CEST spectroscopy experiments were performed as a function of agent concentration, saturation strength, pH, temperature, and Ca^2+^ concentration. As expected, the CEST effect of DANTA increased linearly with agent concentration at pH = 7 (Figure S38). The optimal temperature was approximately 37 °C, which is ideal for *in vivo* applications. The optimal saturation strength was *B*_1_ ≈ 3 μT at 37 °C (Figure 5), in agreement with the theoretical prediction of *k*_ex_ ≈ 2π*B*_1_.

**Figure 5:**
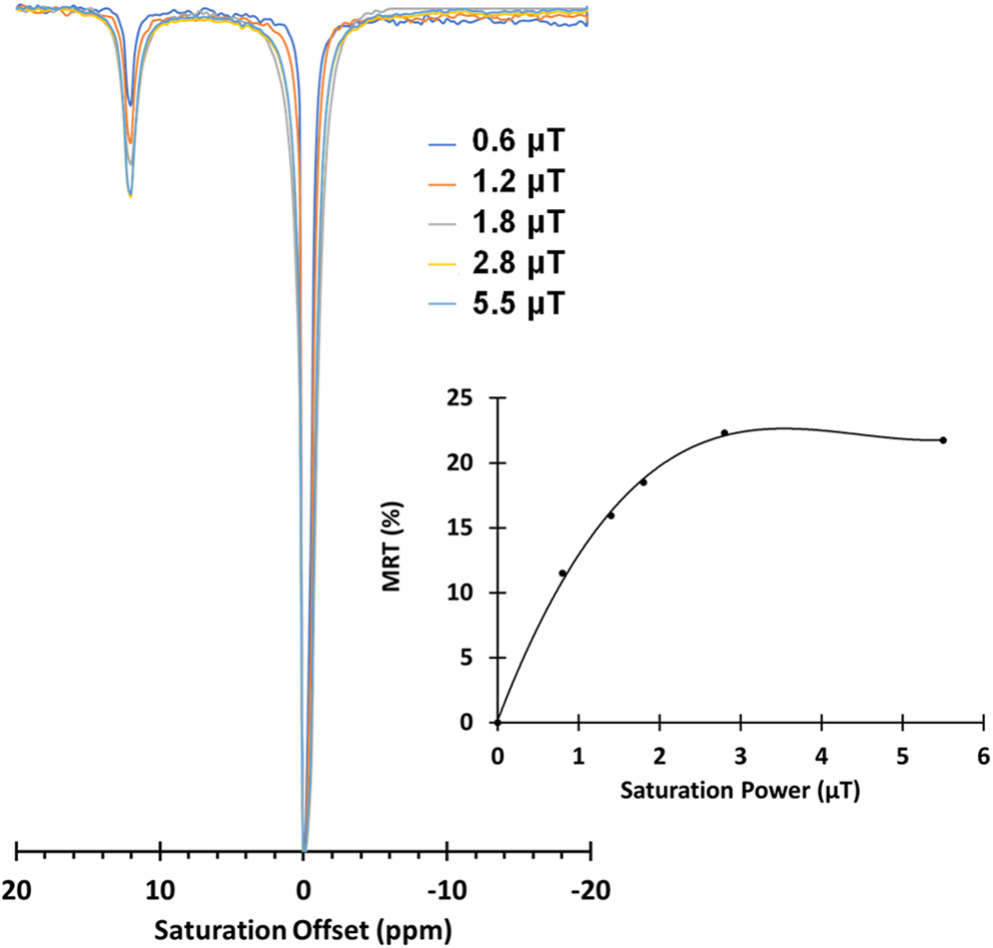
Dependence of CEST intensity on the *B*_1_ saturation strength at 14.1 T at pH 7, 37 °C, 12.5 mM DANTA.

In the absence of Ca^2+^, the CEST signal was essentially independent of pH between pH 6 and 9 at 37 °C (Figure 6A), whereas below pH = 6 the signal decreased due to protonation of both nitrogen atoms. In the presence of Ca^2+^, the magnitude of the CEST signal is governed by the Ca^2+^ + [HDANTA]^3–^ ⇌ [Ca­(DANTA)]^2–^ + H^+^ equilibrium. Because the Ca­(II) complex lacks an exchangeable proton, it does not produce a CEST signal. However, as seen from Table 1, this complex is rather weak and it begins to dissociate below pH = 8, shifting the equilibrium toward the monoprotonated species [HDANTA]^3–^, which gives rise to the CEST signal. Consequently, in the presence of Ca^2+^ the CEST signal depends on both the pH and agent concentration (Figure 6B). The CEST effect correlates well with the speciation of DANTA (Figure 6).

**Figure 6:**
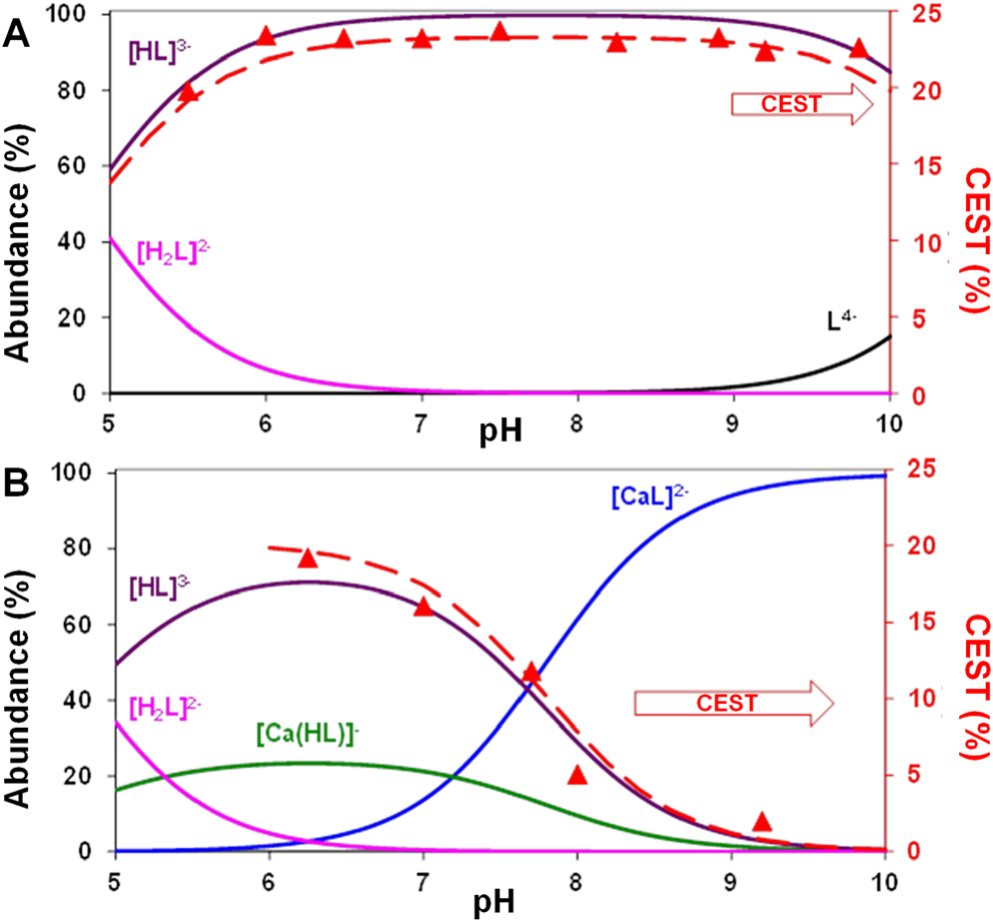
Species distribution of DANTA^4–^ (L) and the effect of pH on the CEST at 37 °C in the absence (A) and in the presence (B) of Ca^2+^ ion ([DANTA] = [Ca^2+^] = 15 mM, 0.15 M NaCl). Graph A shows that the CEST effect is proportional to the concentration of the monoprotonated species [HL]. In the absence of Ca^2+^, the CEST is independent of pH between pH 6 and 9. Graph B shows the CEST (right) and the distribution of Ca^2+^ – DANTA^4–^ system (left) as a function of pH in the presence of equimolar Ca^2+^ ion calculated from the data summarized in Tables 1, S2 and S3. The CEST effect (red dashed line) correlates well with the speciation of [HL].

### CEST Imaging

Phantom imaging experiments performed at 3 T with DANTA and salicylate (Figure 7) demonstrate that DANTA is approximately three to four times more effective than the widely used diaCEST agent salicylate when using *B*_1_ = 3 μT, a saturation strength compatible with clinical imaging. Salicylate exhibits a faster-than-optimal exchange rate, causing its CEST signal to appear as a shoulder on the downfield side of the water resonance. In contrast, DANTA produces a well-resolved CEST peak.

**Figure 7:**
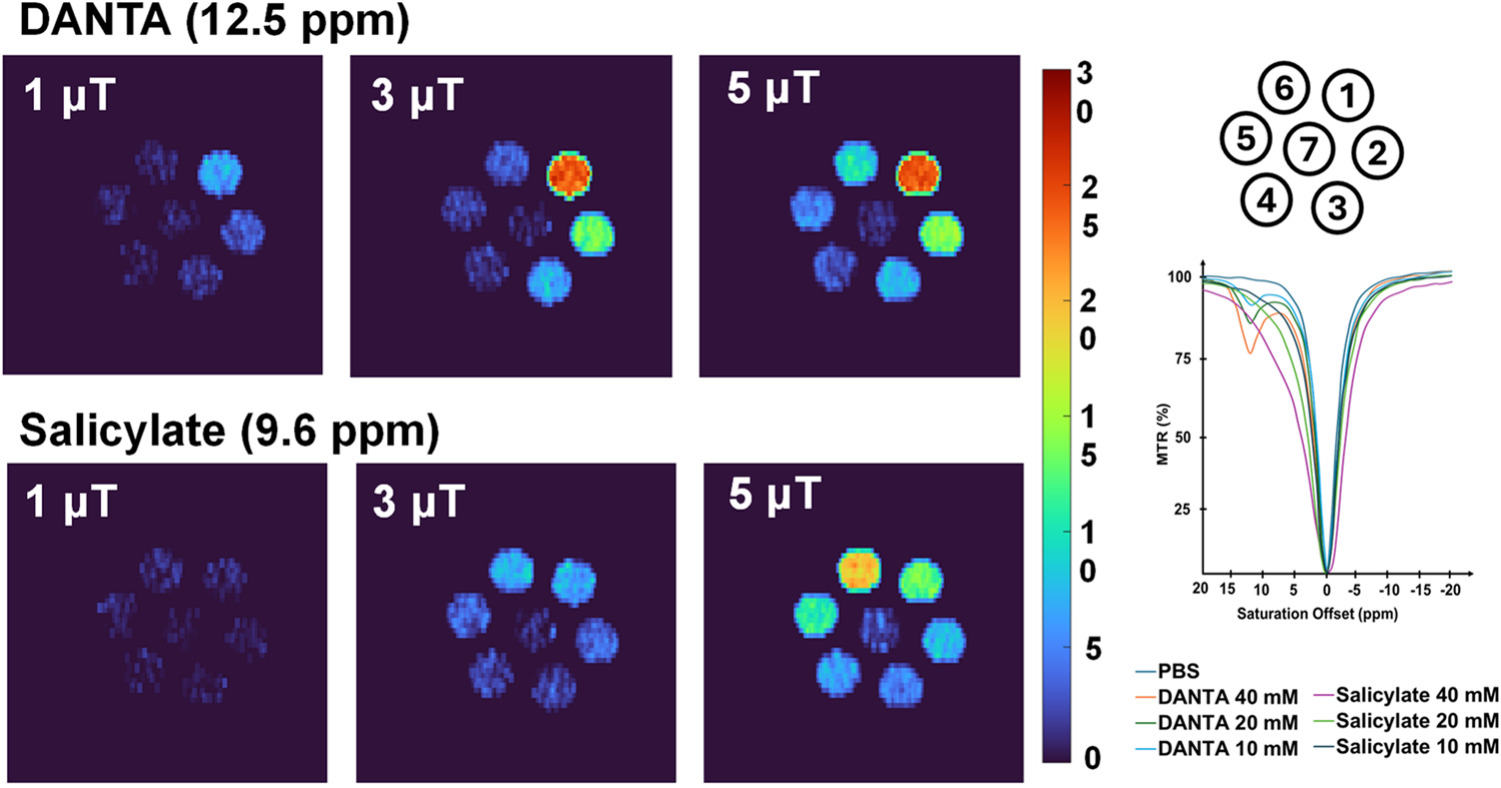
Phantom CEST MR imaging with DANTA and salicylate was performed at 3 T, 37 °C, and pH 7. The phantom consisted of three tubes (1–3) containing DANTA at 40, 20, and 10 mM, respectively; three tubes (4–6) containing salicylate at 10, 20, and 40 mM, respectively; and tube 7 containing PBS buffer. RARE pulse sequence was used with three different *B*_1_ saturation strength.

In vivo CEST imaging experiments were performed in healthy mice. To prevent Ca^2+^ sequestration, the agent was coadministered intravenously at a dose of 1 mmol kg^–1^ (200 μL of a 150 mM solution, pH = 7) with CaCl_2_ at molar ratios of 1.33, 0.66, and 0.33. No adverse effects were observed in mice. CEST imaging was performed at 3 T using a mouse volume coil and a RARE pulse sequence with a *B*_1_ saturation strength of 3 μT applied from −20 to +20 ppm in 2.5 ppm increments. Representative images are shown in Figure 8. Strong CEST contrast enhancement is observed in the renal pelvis, and high-quality images of the kidneys and bladder were obtained at clinical field strength (3 T).

**Figure 8:**
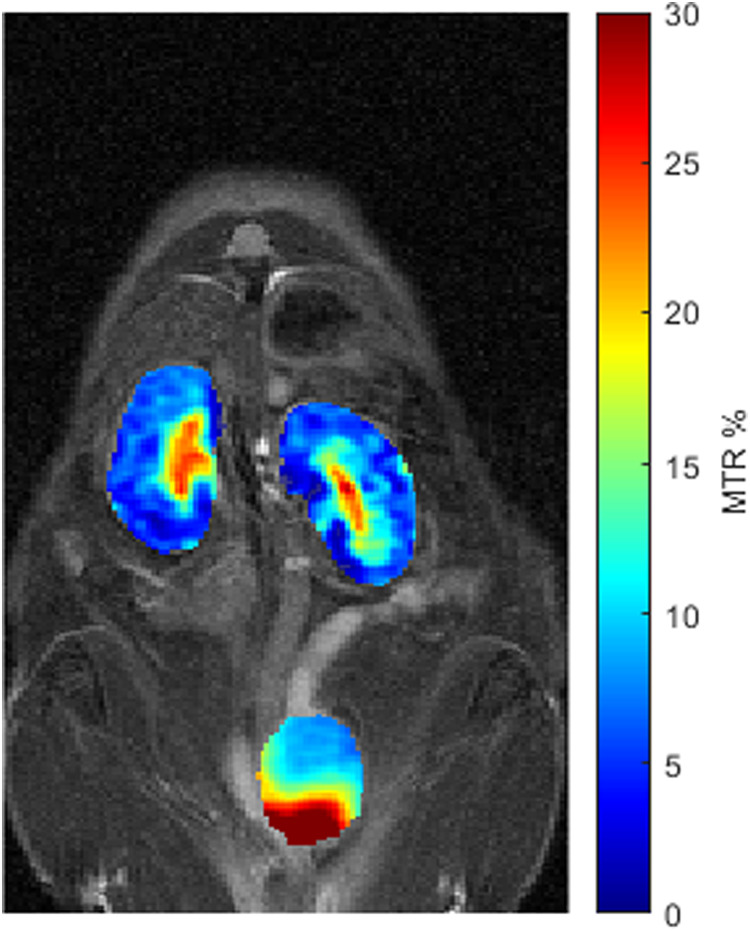
In vivo CEST imaging in mice with DANTA at 3 T. Dose: approximately 1 mmol/kg body weight DANTA plus 0.33 mmol/kg body weight CaCl_2_ (200 μL, 150 mM DANTA and 50 mM CaCl_2_ in pH 7 PBS buffer). Imaging parameters: RARE sequence, *B*_1_ = 3 μT, *t*_sat_ = 5 s, TR = 5.240 s, TE *=* 6.7 ms, RARE factor = 32. The CEST images are overlaid onto *T*_2_-weighted anatomical reference images.

## Conclusions

The labile proton in monoprotonated tetraalkyl 1,8-diaminonaphthalenes is locked a strong N···H^+^···N hydrogen bond that generates an extraordinarily large downfield shift in the proton resonance frequency, but it also dramatically slows proton transfer. We synthesized a novel proton sponge derivative with four acetate side arms. X-ray structural studies reveal that the incorporation of carboxylate functionalities establishes a hydrogen-bond network with the central N···H^+^···N bridge that facilitates proton exchange with the bulk water via intramolecular catalysis. Selective magnetization transfer experiments and kinetic analysis indicate that the acetate side arms accelerate the exchange by several orders of magnitude compared to the parent compound DMAN without affecting the chemical shift of the exchanging proton. The resulting exchange rate (*k*_ex_ ≈ 5 × 10^2^ s^–1^ at 310 K) combined with the large Δ*ω* (12.5 ppm) falls within the optimal regime for CEST contrast generation at clinically relevant *B*_0_ fields and allowable *B*_1_ saturation strengths. Consistent with these properties, DANTA produces strong and well-resolved CEST contrast at 3 T and generates exquisite *in vivo* CEST MRI images in the kidneys and bladder. Moreover, the magnitude of the CEST effect exhibited pH sensitive behavior in the physiologically relevant pH range via the pH-dependent formation/dissociation of the [Ca­(DANTA)]^2–^ complex. This unexplored mechanism for pH sensing could form the basis of novel pH-responsive diaCEST probes. More broadly, this work demonstrates that rational control of proton exchange through intramolecular catalysis offers an effective strategy for fine-tuning proton exchange kinetics in the design of next-generation CEST contrast agents optimized for clinical MRI.

## Supplementary Material



## Notes

Details on the synthesis of DANTA, sample preparation, characterization, experimental procedures for equilibrium studies, proton exchange investigations, CEST and MR imaging, and X-ray diffraction studies of the [Na_3_(HDANTA)] (PDF)
