# Supplementary material for: Accelerating Proton Exchange in 1,8-Bis(dialkylamino)naphthalene Proton Sponges through Intramolecular Catalysis for CEST MRI
Source: J Am Chem Soc. 2026 May 25;148(22):23325–33. doi: 10.1021/jacs.6c06687 (PMC13421967; doi:10.1021/jacs.6c06687)
Supplement: Supplementary file 1 [file ja6c06687_si_001.pdf]

Supporting Information

Accelerating proton exchange in 1,8-bis(dialkylamino)naphthalene proton sponges through intramolecular catalysis for CEST MRI

Mai T. Huynh,[1] Madalina Ranga,[2] Sara Chirayil,[1] Janaka Wansapura,[1] Elena Vinogradov,[1,3] Xiaodong Wen,[1] Mariangela Boccalon,[2] Attila Benyei,[4] James Ratnakar,[1] Zsolt Baranyai,[2,] Zoltan Kovacs[1,*]*

[1] Advanced Imaging Research Center, University of Texas Southwestern Medical Center, 5323 Harry Hines Boulevard, Dallas, Texas 75390 (US)
[2] CRB Trieste, Bracco Imaging SpA, S. S. 14 km 163,5 AREA Science Park, 34149 Basovizza (TS), Italy.
[3] Department of Radiology, UTSW Medical Center, Dallas, TX, United States
[4] Department of Physical Chemistry, University of Debrecen, Egyetem tér 1, H-4032 Debrecen, Hungary

KEYWORDS: CEST, proton sponge, proton exchange, intramolecular catalysis, MRI

*Corresponding author email:

zsolt.baranyai@bracco.com; zoltan.kovacs@UTSouthwestern.edu

1. Examples of CEST agents 3
2. Synthesis 4
2.1. Materials and Methods 4
2.2. 1,8-Diaminonaphthalene-N,N,N',N'-tetraacetic acid ethyl ester 4
2.3. 1,8-Diaminonaphthalene-N,N,N',N'-tetraacetic acid (DANTA) 4
2.4. Characterization of DANTA 4
3. Protonation and complexation equilibria of DANTA 7
3.2. Acid-base properties of H_4DANTA ligand 7
3.3. Complexation properties of DANTA 12
4. Interaction between $[Ca(DANTA)]^{2-}$ and HSA with the formation of HSA-$[Ca(DANTA)]^{2-}$ adduct 13
4.1. Experimental 13
4.2. Interaction between $[Ca(DANTA)]^{2-}$ and HSA 14
5. Proton exchange measurements 16
5.1. Experimental 16

5.2. Proton exchange properties of $HDANTA^{3-}$ 17
5.3. Proton exchange properties of $HDMAN^{+}$ 25
5.4. Proton exchange properties of salicylate 30
6. X-ray diffraction studies of $HDANTA^{3-}$ 34
6.1. Experimental 34
6.2. X-ray structure of the mono-protonated $HDANTA^{3-}$ 35
7. In vitro and in vivo MRI investigation of $HDANTA^{3-}$ 41
7.1. CEST NMR spectroscopy 41
7.2. CEST spectroscopy experiments in the presence of Ca^{2+} 42
7.3. Phantom imaging 42
7.4. In vivo CEST MRI 42
8. References 43

1. Examples of CEST agents

Table S1. Examples of reported CEST agents

Agent	Conditions (temp., pH)	Δω (ppm)	k_{ex} (s^{-1})
EuDOTA(Gly)4 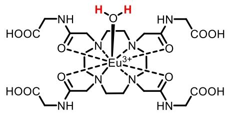	37 °C, pH 7.4 Ref [1]	49.8	10752
5,5'-Azodisalicylic acid (Olsalazine) 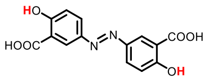	37 °C, pH 7.4 Ref [2]	9.8	697
Salicylic acid 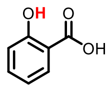	37 °C, pH 7.0 Ref [3,4]	9.3	1200
4,5-Bis[(Glu)carbonyl]-1H-imidazole 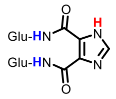	37 °C, pH 7.5 Ref [5]	7.8	5300
Iopamidol 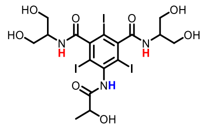	37 °C, pH 6.5 Ref [6–8]	5.5	265
Barbituric acid 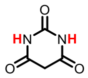	37 °C, pH 7 Ref [9–11]	5.0	900
Tetraphenylporphin sulfonate 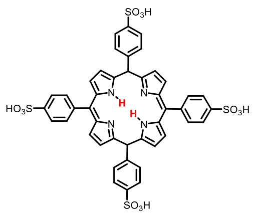	37 °C, pH 6.6 Ref [12]	-9.8	430

2. Synthesis

2.1. Materials and Methods

All commercially available reagents and solvents were purchased from Sigma Adrich and used as received. Nuclear magnetic resonance (NMR) spectra were recorded on Bruker 400 or 600 MHz spectrometer at ambient temperature unless otherwise noted. High resolution mass spectra (HRMS) were recorded using a Water Acquity RDa TOF LC/MS instrument.

2.2. 1,8-Diaminonaphthalene-N,N,N',N'-tetraacetic acid ethyl ester

Anhydrous potassium carbonate (6.21 g, 45 mmol) was added to a stirred solution of 1,8-diaminonaphthalene (1.58 g, 10 mmol) in acetonitrile (50 mL) followed by ethyl bromoacetate (7.52 g, 45 mmol). The reaction mixture was refluxed for 96 hours. Additional ethylbromoacetate (0.84 g, 5 mmol) was added at 24 hours and 48 hours to drive the reaction to completion. After cooling to room temperature, the mixture was filtered and the solvent was removed by rotary evaporation. The crude residue was purified by silica gel column chromatography (20% ethyl acetate in hexanes) to afford 1,8-diaminonaphthalene-N,N,N',N'-tetraacetic acid ethyl ester as a pink viscous liquid (4.2 g, 83.4%) which crystallized to pink crystals on standing. ^{1}H NMR (400MHz, $CDCl_3$, 298K): δ 7.45 (d, 2H), 7.26 (t, 2H), 7.1 (d,2H), 4.30 (dd, 8H), 4.04 (q, 8H), 1.13 (t, 12H) ppm. ^{13}C{^{1}H} NMR (100 MHz, $CDCl_3$, 298K): δ 170.77, 145.75, 138.03, 125.01, 124.80, 121.43, 117.17, 60.26, 55.83, 14.18 ppm. HRMS (ESI-TOF) *m/z*: $[M+H]^+$ Calcd. for $C_{26}H_{35}N_2O_8$ 503.2388; Found 503.2405.

2.3. 1,8-Diaminonaphthalene-N,N,N',N'-tetraacetic acid (DANTA)

A stirred suspension of 1,8-diaminonaphthalene-N,N,N',N'-tetraacetic acid ethyl ester (1.04 g, 2 mmol) in HCl (20%, 10 mL) was refluxed for 24 hours. The ester hydrolysis was monitored using LC-MS. The reaction mixture was filtered hot to remove a small amount of insoluble impurities. The filtrate was allowed to cool overnight at 4 °C. The formed light brown precipitate was filtered, washed with small amount of cold HCl (2M). The crude product was recrystallized from HCl (2M, 10 mL) to obtain the product as white crystalline powder (550 mg, yield 58%). ^{1}H NMR (600 MHz, H_2O, 298K) δ 17.06 (s, 1H), 7.79 (d, 2H), 7.72 (d, 2H), 7.43 (t, 2H), 4.10 (q, 8H) ppm. ^{13}C{^{1}H} NMR (150 MHz, H_2O, 298K, Figure S1) δ 173.94, 143.03, 135.19, 128.55, 126.16, 122.89, 121.86 ppm. HRMS (ESI-TOF) *m/z*: $[M+H]^+$ Calcd for $C_{18}H_{19}N_2O_8$: 391. 1136, Found: 391.1212 Anal. Calcd for $C_{18}H_{18}N_2O_8$ x 1 HCl x 2.6 H_2O: C, 45.65; H, 5.15; N, 5.91; Cl, 7.48. Found: C, 45.33; H, 5.01; N, 5.94; Cl, 7.72

2.4. Characterization of DANTA

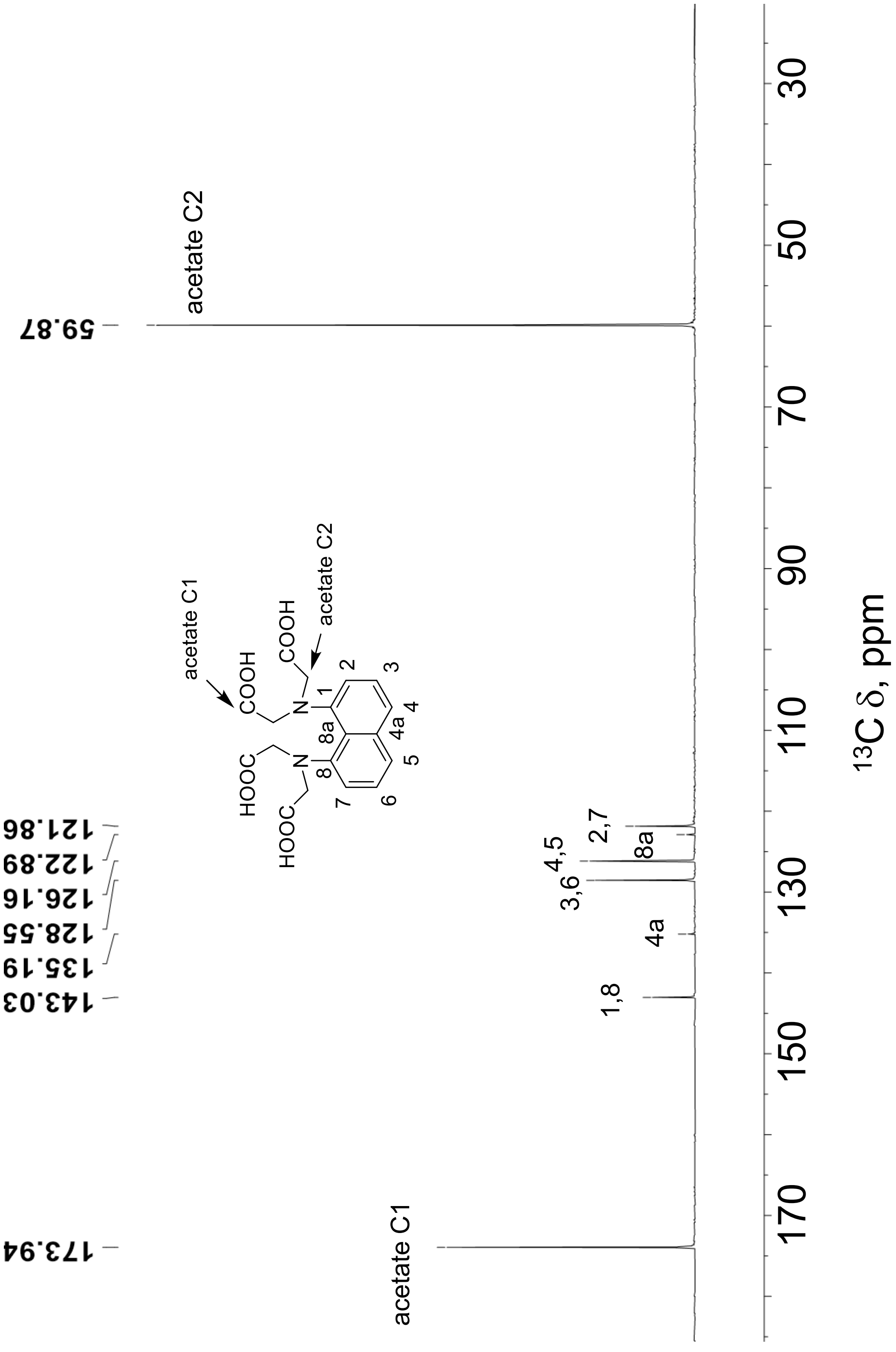

Figure S1. ^{13}C NMR spectra of DANTA at 14.1 T and 298K in H_2O.

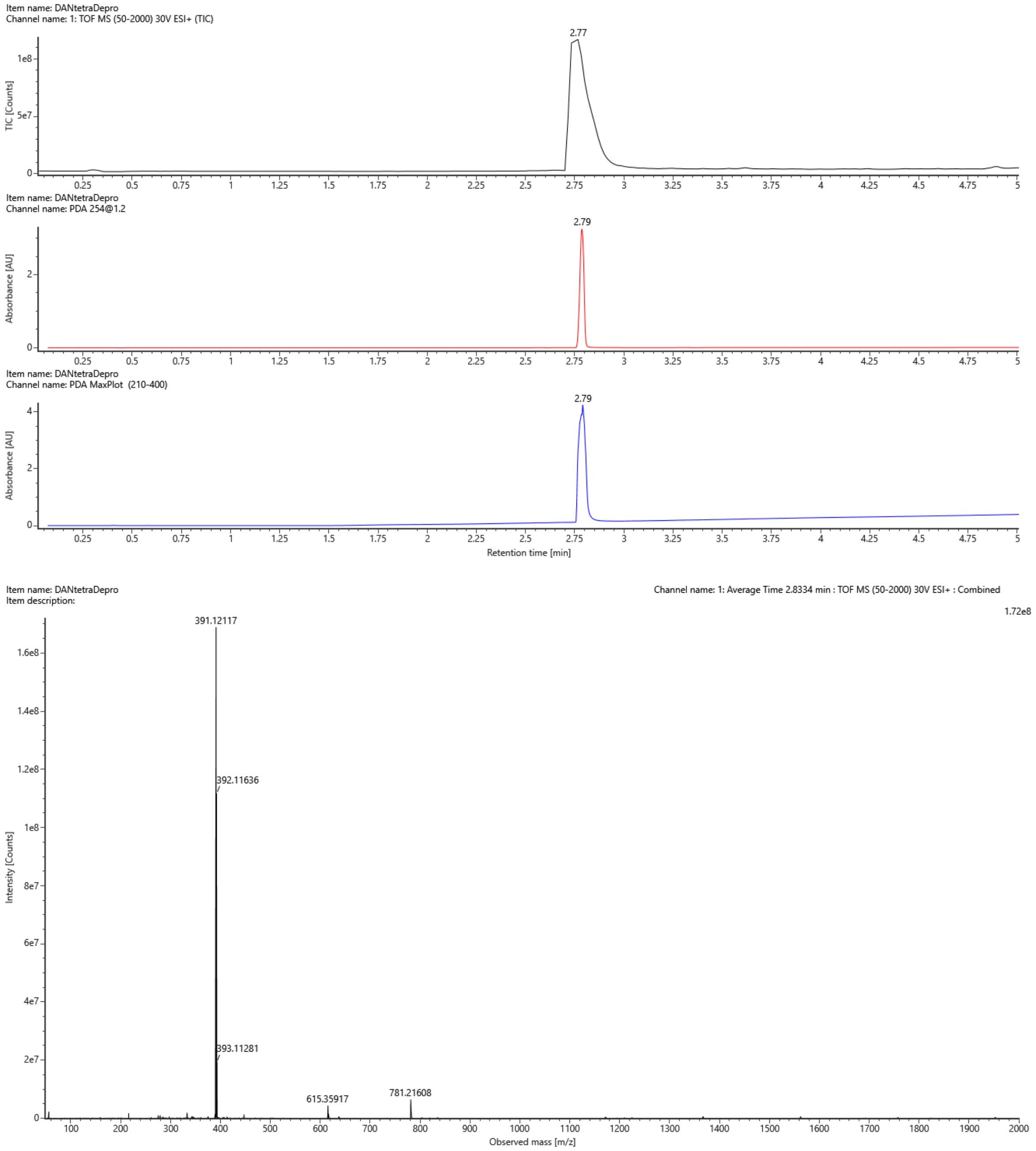

Figure S2. LC-MS chromatogram of DANTA (C18 reversed phase column, acetonitrile-water 1:1 isocratic elution).

3. Protonation and complexation equilibria of DANTA

3.1. Experimental

Materials: The chemicals used for the experiments were of the highest analytical grade. The concentration of the $CaCl_2$, $ZnCl_2$, $CuCl_2$ solutions were determined by complexometric titration with standardized Na_2H_2EDTA and *xylenol orange* ($ZnCl_2$,), *murexid* ($CuCl_2$) and *Patton & Reeder* ($CaCl_2$) as indicators. The concentration of the H_4DANTA and DMAN were determined by pH-potentiometric titration in the presence and absence of a large (40-fold) excess of $CaCl_2$. The pH-potentiometric titrations were made with standardized 0.2 M NaOH.

Equilibrium measurements: The protonation constants of DANTA and DMAN, the stability and protonation constants of metal complexes formed with DANTA ligand were determined by pH-potentiometric titration. The metal-to-ligand concentration ratio was 1:1 (the concentration of the ligand was generally 0.002 M). For the pH measurements and titrations, *Metrohm 888 Titrando* titration workstation *Metrohm-6.0234.110* combined electrode was used. Equilibrium measurements were carried out at a constant ionic strength (0.15 M NaCl) in 6 ml samples at 25 °C. The solutions were stirred, and N_2 was bubbled through them. The titrations were made in the pH range of 1.7-12.0. KH-phthalate (pH=4.005) and borax (pH=9.177) buffers were used to calibrate the pH meter, For the calculation of $[H^+]$ from the measured pH values, the method proposed by *Irving et al.* was used.[13] A 0.01M HCl solution was titrated with the standardized KOH solution in the presence of 0.1 M KCl ionic strength. The differences (A) between the measured (pH_{read}) and calculated pH ($-\log[H^+]$) values were used to obtain the equilibrium H^+ concentration from the pH values measured in the titration experiments (A=0.044). For the equilibrium calculations, the stoichiometric water ionic product (pK_w) was also needed to calculate $[OH^-]$ values under basic conditions. The $V_{NaOH} - pH_{read}$ data pairs of the HCl – NaOH titration obtained in the pH range 10.5 – 12.0 were used to calculate the pK_w value (pK_w=13.74). The protonation and stability constants were calculated with the PSEQUAD program.[14]

3.2. Acid-base properties of H_4DANTA ligand

The protonation constants, defined by Eq. (1), have been determined by pH-potentiometry, ^{1}H-NMR spectroscopy and UV-spectrophotometry.

$$K_i^H = \frac{[H_iL]}{[H_{i-1}L][H^+]} \qquad (S1)$$

where i=1, 2…5. The protonation sequence of DANTA has been determined by ^{1}H-NMR spectroscopy, recording the chemical shift variations of the non-labile protons as a function of pH. The ^{1}H-NMR titration curves (Figure S3) at pH<10 display sharp changes, which are

related to the protonation/deprotonation of the ligand. At pH>10 there are two sets of signals in the ^{1}H NMR spectra of DANTA due to the slow chemical exchange between the monoprotonated $HDANTA^{3-}$ and the deprotonated $DANTA^{4-}$ species at the actual NMR time scale. At pH<10 the protonation/deprotonation is fast on the NMR time scale, the chemical shifts of the observed signals represent a weighted average of the shifts of the different species involved in a specific protonation step (Eq. (S2))[15]:

$$\delta_{\mathrm{H(obs)}} = \sum x_i \delta_{\mathrm{H}}^{\mathrm{H_iL}} \qquad (S2)$$

where, $\delta_{\mathrm{H(obs)}}$ is the observed chemical shift of a given signal, x_{i} and $\delta_{\mathrm{H}}^{\mathrm{HiL}}$ are the molar fraction and the chemical shift of the involved species, respectively. At pH>10, the integral values of the given signal are directly proportional to the concentration of the monoprotonated $HDANTA^{3-}$ or the deprotonated $DANTA^{4-}$ species (Eq. (S3)).

$$\int_{\mathrm{obs}}^{\mathrm{HL}} = \int_{\mathrm{H}}^{\mathrm{HL}} \times [\mathrm{HDANTA}]\ ;\ \int_{\mathrm{obs}}^{\mathrm{L}} = \int_{\mathrm{H}}^{\mathrm{L}} \times [\mathrm{DANTA}] \qquad (S3)$$

where, $\int_{\mathrm{obs}}^{\mathrm{HL}}$, $\int_{\mathrm{obs}}^{\mathrm{L}}$, $\int_{\mathrm{H}}^{\mathrm{HL}}$ and $\int_{\mathrm{H}}^{\mathrm{L}}$ are the observed integral values and the molar integral value of the given signal in $HDANTA^{3-}$ and $DANTA^{4-}$ species, respectively. The protonation/deprotonation of the DANTA has been also studied by spectrophotometry on the absorption band of the aromatic group of the ligand, following the absorbance values at 225, 245 and 340 nm. The UV-spectra and the absorbance values at 225, 245 and 340 nm of the DANTA are shown in Figure S6. The absorbance of the ligand is a combination of the absorption of each protonated species, which can be expressed by Eq. (S4)[16]:

$$\mathrm{A} = \sum [\mathrm{H_iL}] \times \varepsilon_{\mathrm{H}}^{\mathrm{H_iL}} \times l \qquad (S4)$$

where, A is the absorbance at a given wavelength, $[\mathrm{H_iL}]$, $\varepsilon_{\mathrm{H}}^{\mathrm{HiL}}$ and l are the concentration and the molar absorptivity of the species and the path length of the cell, respectively. The observed chemical shift ($\delta_{\mathrm{H(obs)}}$, Figure S4), integral ($\int_{\mathrm{obs}}$, Figure S5) and absorbance values (A, Figure S6) have been fitted to the Eqs. (S2) – (S4), respectively (the molar fractions x_{i} and the concentration of the different protonated species have been expressed by the protonation constants $K_{\mathrm{i}}^{\mathrm{H}}$, Eq. (S1)). The fittings of the experimental data points are also shown in Figures S4 – S6. The obtained $\log K_{\mathrm{i}}^{\mathrm{H}}$ values are listed and compared with those of structurally similar ligands (DMPDTA and PDTA) in Table S2.

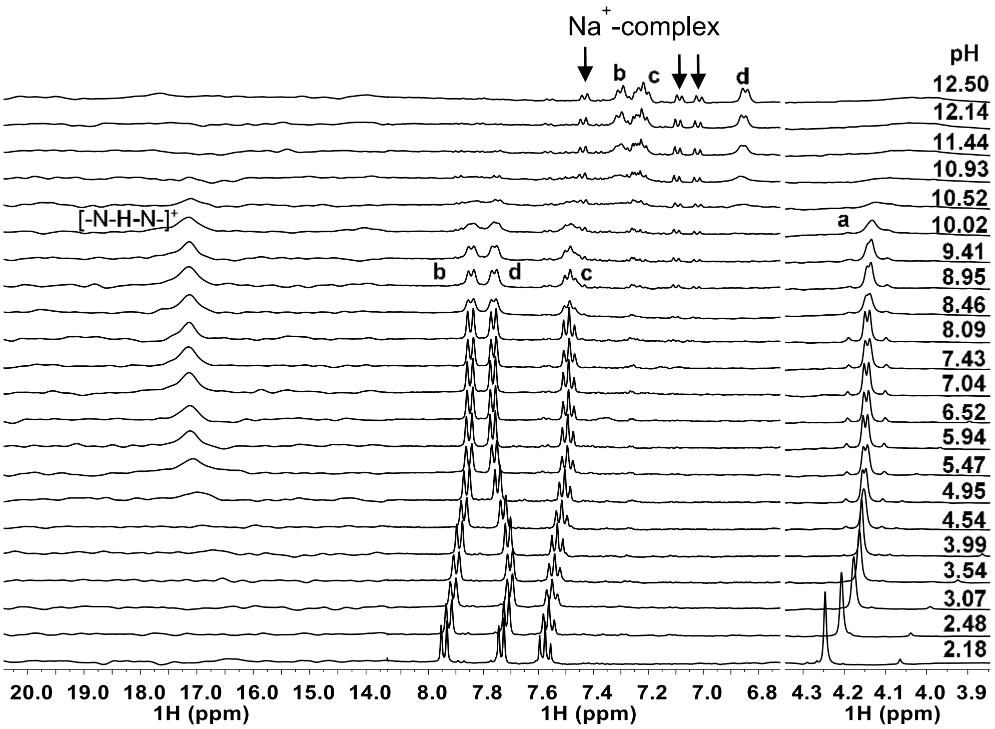

Figure S3. ^{1}H NMR spectra of DANTA as a function of pH ([L]=10 mM, 9.4 T, 298 K, H_2O, 0.15 M NaCl).

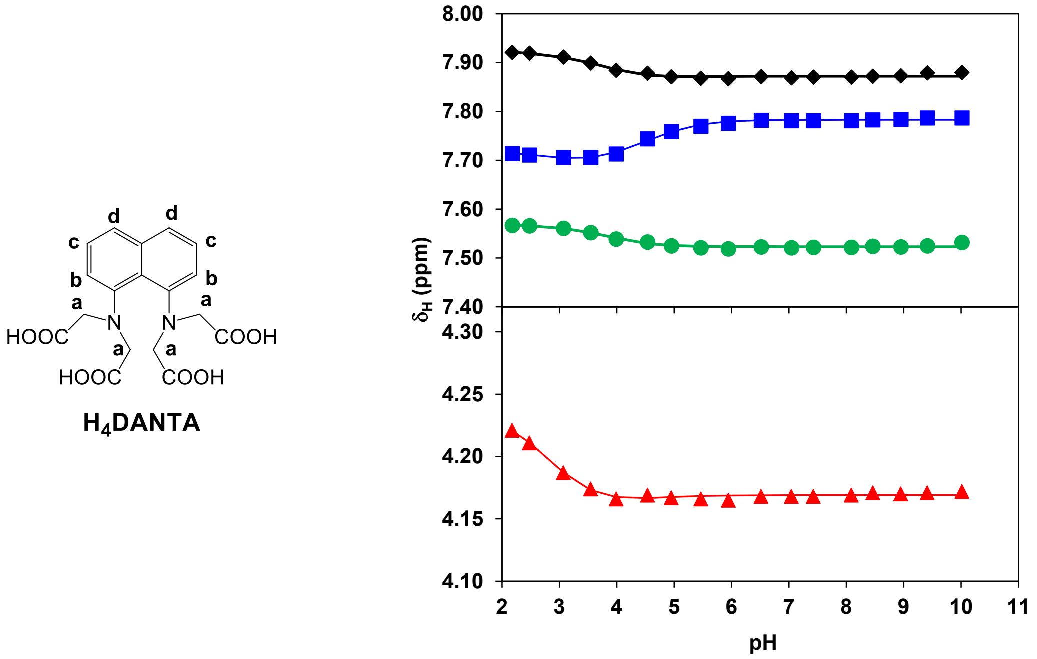

Figure S4. Chemical shifts ($\delta_{H(obs)}$) of CH (◆- b, ■ - d, ● - c) and CH_2 (▲ - a) protons in DANTA. Symbols and solid lines represent the experimental and the calculated chemical shift values, respectively. ([L]=10 mM, H 9.4 T, 298 K, H_2O, 0.15 M NaCl).

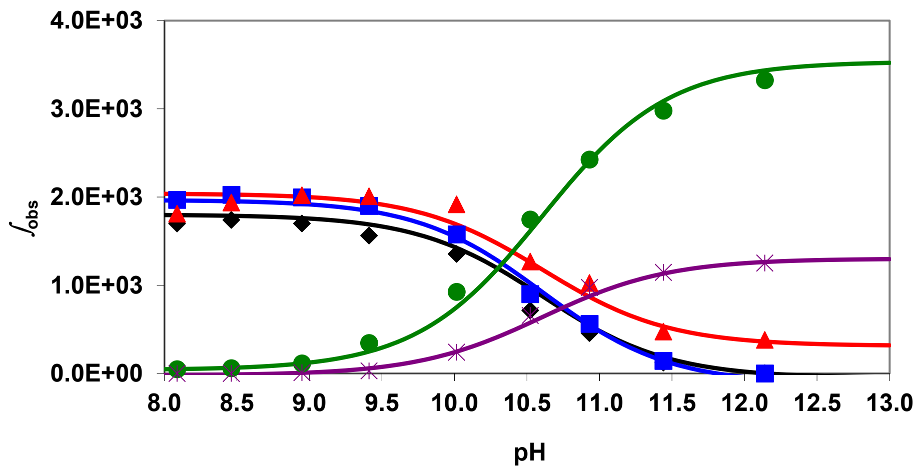

Figure S5. Integral values of -CH protons (◆ - b, ■ - d, ▲ - c) in $HDANTA^{3-}$ and (● - b and c', ✳ - d') in $DANTA^{4-}$ species ([L]=10 mM, 9.4 T, 298 K, H_2O, 0.15 M NaCl).

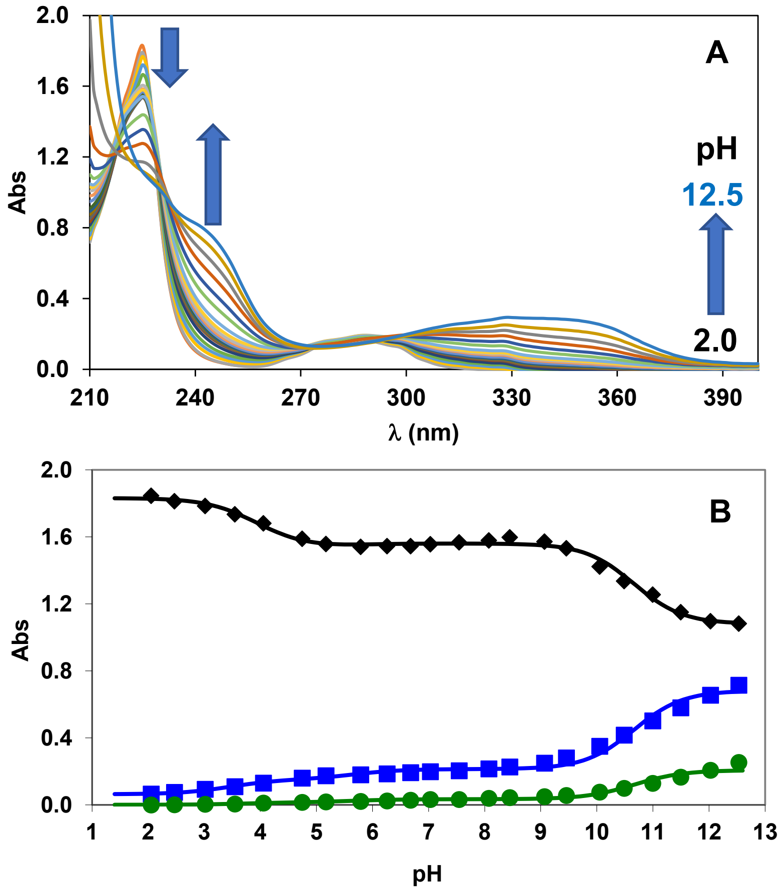

Figure S6. Absorption spectra (**A**) and absorbance values (**B**) of the DANTA ligand as a function of pH at 225 (◆), 245 (■) and 340 nm (●). Solid lines and symbols represent the experimental and the calculated absorbance values, respectively. ([L]=25 μM, 0.15 M NaCl, 25°C).

Table S2. Protonation constants ($\log K_i^H$) of the DANTA, DMPDTA, PDTA and DMAN ligands at (25°C).

	**DANTA**			**DMPDTA**[a]	**PDTA**[a,b]		**DMAN**[c]
	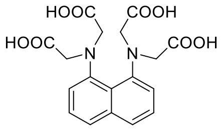			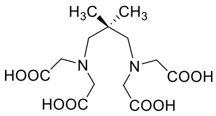	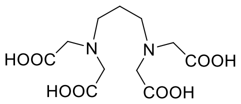		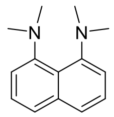
**Method**	pH-pot.	UV-tit.	^{1}H NMR	pH-pot.	pH-pot.		Photomet.
I	0.15 M NaCl			0.1 M KCl	0.1M KCl[a]	0.1M KNO_3[b]	0.1 M
$\log K_1^H$	10.75 (2)	10.70 (3)	10.65 (3)	10.28	10.29	10.46	12.1
$\log K_2^H$	4.84 (5)	4.68 (5)	4.68 (2)	6.52	7.94	8.02	–
$\log K_3^H$	4.00 (5)	4.06 (5)	3.95 (5)	2.66	2.70	2.47	–
$\log K_4^H$	3.15 (5)	3.31 (9)	2.91 (2)	1.93	1.94	1.88	–
$\log K_5^H$	1.70 (6)	–	–	–	–	–	–
$\boldsymbol{\Sigma\log K_{1\text{-}4}^H}$	**22.74**	**22.75**	**22.19**	**21.39**	**22.86**	**22.81**	–

[a] Ref. [17]; [b] Ref. [18,19]; [c] Ref. [20]

In the ^{1}H-NMR spectra of DANTA the methylene protons of the acetate groups (*a*) and the methyl protons of the naphthalene (*b, d* and *c*) give rise to AB doublet, two doublets and the triplet at pH=7 (Figure S3). The N···H^+···N proton in the monoprotonated species appears as a broad singlet at 17.2 ppm in the pH range 4.0 – 11.4. To determine the protonation scheme of $DANTA^{4-}$, the ^{1}H NMR and UV-spectrophotometric titration curves (Figures S3-S6) have been analyzed simultaneously. The addition of one equivalent of acid to $DANTA^{4-}$ results in a new set of the NMR signals of the *a*, *b*, *c* and *d* protons (Figures S3 and S5) due to the slow chemical exchange between the $DANTA^{4-}$ and $HDANTA^{3-}$ species on the NMR time scale. Since the UV absorption spectra of the ligand show changes in the same pH range (Figure S6), it was assumed that the first protonation takes place on both nitrogen atoms (both nitrogen atoms are partially protonated) of the 1,8-diamino-naphthalene backbone. In the pH range 6 – 10, the chemical shifts *a*, *b*, *c* and *d* protons and the absorbance values of the $HDANTA^{3-}$ are unchanged, which indicate the absence of protonation processes (Figure S4). The methylene protons of the acetate groups (*a*) in $HDANTA^{3-}$ have a well resolved AB doublet, which is attributed to the hindered rotation of the acetate pendant arms due to the intramolecular H-bonding with the protonated nitrogen atoms (Figure S3). In the pH range 3 – 6, the second protonation process results in the downfield shifts of the *b*, *c* and *d* methyl proton signals,

confirming that the $\log K_2^H$ value is related to the protonation of the nitrogen atom in the ligand backbone (Figure S4). The protonation of the second nitrogen atom is evidenced by the changes of the absorbance values in the pH range 3 – 6 (Figure S6). Further lowering of pH induces a strong downfield shift of the methylene protons in the acetate pendant arms due to the protonation of the carboxylate groups (Figure S4). The AB doublet of the acetate methylene protons transforms into a broad singlet in the last three protonation steps of the second N-atom and the carboxylates. (Figure S3). The protonation constants of the DANTA ligand obtained by ^{1}H NMR and UV-spectrophotometric studies agree well with those determined by pH-potentiometry (Table S2).

3.3. Complexation properties of DANTA

The stability and protonation constants of the metal complexes formed with DANTA ligand are defined by Eqs. (S5) and (S6):

$$K_{\mathrm{ML}} = \frac{[\mathrm{ML}]}{[\mathrm{M}][\mathrm{L}]} \quad \text{(S5)}$$

$$K_{\mathrm{MH_iL}} = \frac{[\mathrm{MH_iL}]}{[\mathrm{MH_{i-1}L}][\mathrm{H^+}]} \quad \text{(S6)}$$

where i=1, 2, 3. The K_{ML} and K_{MHiL} values characterizing the formation of DANTA complexes with Ca^{2+}, Zn^{2+} and Cu^{2+} have been calculated from the pH-potentiometric titration data obtained at 1:1 metal to ligand concentration ratios. When the equilibrium constants were calculated, the best fit of the volume of NaOH (mL) – pH data was obtained by assuming the formation of ML and MHL species. The stability and protonation constants of these complexes with DANTA are presented and compared with those of DMPDTA and PDTA ligands in Table S3.

Table S3. Stability ($\log K_{\mathrm{ML}}$) and protonation constants ($\log K_{\mathrm{MHL}}$) of the Ca(II)-, Mn(II)-, Zn(II)-, Cu(II)-, and Ln(III)-complexes formed with DANTA, DMPDTA and PDTA ligands (25°C).

	**DANTA**		**DMPDTA**[a]		**PDTA**	
I	0.15 M NaCl		0.1 M KCl		0.1 M KCl[a] / 0.1 M KNO_3[b]	
	**log*K*ML**	**log*K*MHL**	**log*K*ML**	**log*K*MHL**	**log*K*ML**	**log*K*MHL**
**Ca^{2+}**	5.68 (6)	7.19 (8)	6.60	–	7.28[b]	6.28[b]
**Mn^{2+}**	7.79 (3)	5.19 (8)	10.55	–	10.01[b]	5.29[b]

**Zn^{2+}**	13.33 (2)	2.95 (3)	15.62	2.33	15.30[a] /15.26[b]	2.39[a] / 2.50[b]
**Cu^{2+}**	17.72 (2)	2.76 (5)	20.06	2.55	19.65[a] /18.80[b]	2.81[a] / 2.20[b]

[a] Ref. [17]; [b] Ref. [18,19]; $[Y(DANTA)]^-$: $\log K_{YL}$=9.74 (5) (0.15 M NaCl, 25°C)

By taking into account the protonation constants of DANTA ligand (Table S2), the stability and protonation constant of $[Ca(DANTA)]^{2-}$ complex (Table S3), the pH dependent species distribution of the Ca^{2+} - DANTA system has been calculated and plotted in Figure S7.

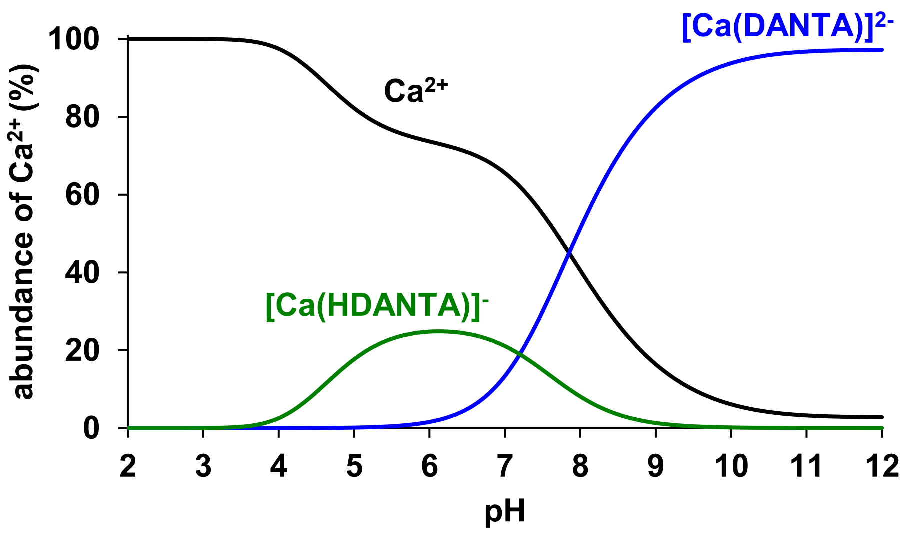

Figure S7. Species distribution of Ca^{2+} - DANTA system as a function of pH. The calculation of the species distribution was performed by using the equilibrium constants in Table 1. ($[Ca^{2+}]$=[DANTA]=2.0 mM, 0.15 M NaCl, 25°C).

4. Interaction between $[Ca(DANTA)]^{2-}$ and HSA with the formation of HSA-$[Ca(DANTA)]^{2-}$ adduct

4.1. Experimental

The interactions between $[Ca(DANTA)]^{2-}$ and HSA with the formation of HSA-$[Ca(DANTA)]^{2-}$ adduct have been studied with ultrafiltration by measuring the free $[Ca(DANTA)]^{2-}$ with Capillary Zone Electrophoresis (CZE) method. 1 mL 0.5 mM $[Ca(DANTA)]^{2-}$ complex was prepared at pH=7.4 and 25°C in the presence of 0.0 – 3.0 mM HSA in 0.15 M NaCl solution. Samples were ultrafiltered with Amicon Ultra-0.5 Centrifugal Filter 10 kDa MWCO (*Millipore*) for 10000 rmp and 30 s in order to separate ca. 20 μL solution

(the withdrawal of 20 μL solution from 1.0 mL sample has negligible effect for the speciation of the sample). Low molecular weight fraction was analyzed with CZE method. *Agilent 7100* capillary electrophoresis system was used in the CZE experiments. Separations were performed using bare fused-silica capillaries of 56 cm × 50 μm i.d. (*Agilent*). Before the first use of the capillary it was washed with 1.0 M NaOH (15 min), with 0.1 M NaOH (30 min) and with the buffer electrolyte (30 min). Prior to CZE analysis all buffers were filtered through a 0.45 μm syringe filter and stored in refrigerator at +4ºC. In all CZE experiments, the sample solutions were introduced at the anodic end of the capillary (50 mbar, 20 s). The migration time of DANTA is ca. 7 min. The capillary was preconditioned with the buffer electrolyte (50 mM sodium tetraborate, pH=9.2) for 3 minutes. The separation was performed at 25°C with the application of 30 kV voltage. After analysis, postconditioning [0.1 M NaOH (3 min) and buffer (3 min)] was applied to remove all possibly adsorbed materials from the capillary. In all measurements, 5 mM DMSO (migration time of DMSO ca. 2 min) as internal standard was applied in order to correct the injection and the migration time of components in the electropherogram. The detection was carried out by on-column DAD measurement at 220 nm. The electropherograms were recorded and processed by *ChemStation* computer program of C.01.09 version (*Agilent*). The equilibrium calculations have been performed with the *Micromath Scientist* computer program (version 2.0, Salt Lake City, UT, USA).

4.2. Interaction between $[Ca(DANTA)]^{2-}$ and HSA

$Area_{DANTA}$ / $Area_{DMSO}$ values obtained by CZE studies as a function of $[HSA]_{tot}$ in $[Ca(DANTA)]^{2-}$ and HSA systems are shown in Figure S8.

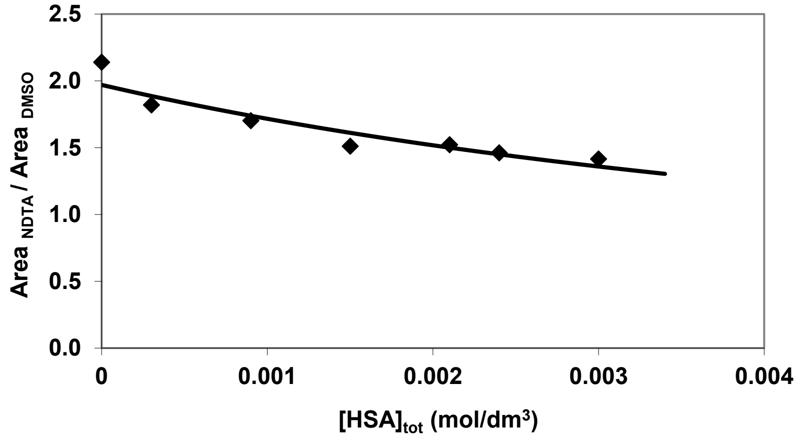

Figure S8. $Area_{NDTA}$ / $Area_{DMSO}$ values (◆) as a function of $[HSA]_{tot}$ in $[Ca(DANTA)]^{2-}$ – HSA systems. The filled symbols and the line represent the experimental and the calculated area values, respectively. ([Ca(DANTA)]=0.5 mM, pH=7.4, 25°C, 0.15 M NaCl).

In Figure S8, the $Area_{DANTA}$ / $Area_{DMSO}$ values decrease by increasing the $[HSA]_{tot}$, which is due to the decrease of $[Ca(DANTA)]^{2-}$ in the low molecular weight fraction. Based on the species distribution of the Ca^{2+} - DANTA system, Ca(II)-complex and the free DANTA ligand are present at pH=7.4 in solution (Figure S7). Since the ligand exchange between the free DANTA and the $[Ca(DANTA)]^{2-}$ complex is fast under the conditions CZE, the area of the signal at around 7 min migration time is directly proportional to the sum of $[DANTA]_{free}$, [Ca(DANTA)] and [Ca(HDANTA)].

$$\text{Area} = f_B \times [B]_{free} \qquad (S7)$$

where f_B and $[B]_{free}$ are the molar area value of the DANTA containing species and the sum of the concentration of DANTA containing free species ($[B]_{free}$= $[DANTA]_{free}$+[Ca(DANTA)]+[Ca(HDANTA)]) in the low molecular weight fraction. The interaction between HSA (A) and DANTA containing species (B) can be described by Eq. (S8)

$$A + B \overset{K_a}{\rightleftharpoons} AB \qquad (S8)$$

$$K_a = \frac{[AB]}{[A]_{free}[B]_{free}}$$

where $[A]_{tot}=[A]_{free}+[AB]$, $[B]_{tot}=[B]_{free}+[AB]$ and [AB] is the concentration of the adduct formed by HSA and DANTA containing species. By taking into account Eq. (S8), the equilibrium constant characterizing the formation of the adduct between HSA and DANTA containing species (K_a) can be expressed with Eq. (S9).

$$K_a = \frac{[AB]}{([A]_{tot}-[AB])([B]_{tot}-[AB])} = \frac{[AB]}{[A]_{tot}[B]_{tot}-([A]_{tot}+[B]_{tot})[AB]+[AB]^2} \qquad (S9)$$

$$K_a[AB]^2-[AB](K_a[A]_{tot}+K_a[B]_{tot}+1)+K_a[A]_{tot}[B]_{tot}=0$$

In S9, [AB] is the equilibrium concentration of the adduct formed by HSA and DANTA containing species, which can be expressed with Eq. (S10):

$$[AB] = \frac{K_a[A]_{tot}+K_a[B]_{tot}+1-\sqrt{(K_a[A]_{tot}+K_a[B]_{tot}+1)^2-4K_a^{\,2}[A]_{tot}[B]_{tot}}}{2K_a} \qquad (S10)$$

By taking into account Eq. (S7), the area values of the DANTA containing species in the low molecular weight fraction can be expressed withy Eq. (S11).

$$\text{Area} = f_{\mathrm{B}} \times \left([\mathrm{B}]_{\mathrm{tot}} - \frac{K_a[\mathrm{A}]_{\mathrm{tot}}+K_a[\mathrm{B}]_{\mathrm{tot}}+1-\sqrt{(K_a[\mathrm{A}]_{\mathrm{tot}}+K_a[\mathrm{B}]_{\mathrm{tot}}+1)^2-4K_a{}^2[\mathrm{A}]_{\mathrm{tot}}[\mathrm{B}]_{\mathrm{tot}}}}{2K_a} \right) \quad \text{(S11)}$$

The K_a and f_B values have been calculated by fitting of the $Area_{NDTA}$ / $Area_{DMSO}$ values to Eq. (S11), obtained at pH=7.4 and 25°C in the presence of 0.5 mM $[Ca(DANTA)]^{2-}$ and 0.0 – 3.0 mM HSA (Figure S8). The K_a and f_B values were found to be 158 ± 32 M^{-1} and 3942 ± 148 mAU, respectively. Species distribution of the $[Ca(DANTA)]^{2-}$ - HSA systems as a function of $[HSA]_{tot}$ are shown in Figure S9.

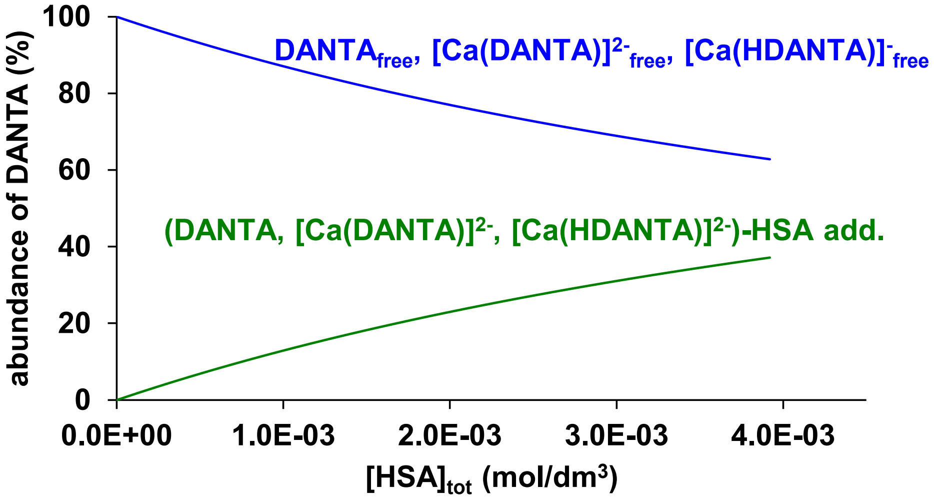

Figure S9. Species distribution of the $[Ca(DANTA)]^{2-}$ – HSA system as a function of $[HSA]_{tot}$ ($[Ca(NDTA)]$=0.5 mM, pH=7.4, 25°C, 0.15 M NaCl).

5. Proton exchange measurements

5.1. Experimental

The structural behavior and the proton exchange processes of the mono-protonated $HDANTA^{3-}$, $HDMAN^{+}$ and salicylate were followed by ^1H NMR measurements. NMR experiments were performed by Bruker Avance III (9.4 T) spectrometer, equipped with Bruker Variable Temperature Unit (BVT), Bruker Cooling Unit (BCU) and a BB inverse *z* gradient probe (5 mm). For these experiments, a 0.012 M aqueous solution of $HDANTA^{3-}$, $HDMAN^{+}$ and salicylate was prepared in the presence of 0.15 M NaCl (a capillary with D_2O was used for lock). The pH was adjusted by stepwise addition of concentrated NaOH and HCl solutions (both prepared in H_2O). The chemical shifts are reported in ppm, relative to DSS for ^1H as the external

standard. The chemical exchange between the $HDANTA^{3-}$, $HDMAN^{+}$ or salicylate and H_2O protons was investigated with selective inversion transfer experiments by using a DANTE pulse train[21] in the pH range 4.5 – 9.5 and in the temperature range 273 – 343 K. The DANTE pulses were typically 0.5 – 1.0 μs long and the selectivity was regulated by the delay time between the pulses. In each series of experiments, ^1H NMR signal of the H_2O, the $N\cdots H^{+}\cdots N$ proton of $HDANTA^{3-}$ or $HDMAN^{+}$ and the phenolate-OH of salicylate was inverted by a 180° pulse and then both signals were observed using a τ time delayed 90° pulse. The DANTE experiments were performed with 15 s relaxation delay (full relaxation). In the consequent calculations, both series of experiments were treated in parallel. For quantitative analysis the integrals of the deconvoluted peaks were used. The pulse imperfections were corrected by treating the initial and equilibrium magnetizations as adjustable parameters in the least-squares refinements. Calculations were performed by the fitting of the integrals of the deconvoluted peaks – τ delay time data pairs with the computer program *Micromath Scientist*, version 2.0 (Salt Lake City, UT, USA).

5.2. Proton exchange properties of $HDANTA^{3-}$

In order to studies the exchange between the $HDANTA^{3-}$ and H_2O protons (Eq. S14) selective inversion transfer experiments at various temperature and pH values (Figure S10 and S11) were performed by using a DANTE pulse train. ^1H NMR spectra of $HDANTA^{3-}$ obtained by the selective excitation of the H_2O and $N\cdots H^{+}\cdots N$ proton of $HDANTA^{3-}$ at pH=7.07 and 273 K are shown in Figures S12 and S14. Absolute integral values of the H_2O and $N\cdots H^{+}\cdots N$ proton of $HDANTA^{3-}$ as a function of τ delay time obtained at pH=7.07 and 273 K are shown in Figures S13 and S15.

$$[N\text{-}H^*\text{-}N]^+ + HOH \underset{v_-}{\overset{v_+}{\rightleftharpoons}} [N\text{-}H\text{-}N]^+ + H^*OH \qquad (S14)$$

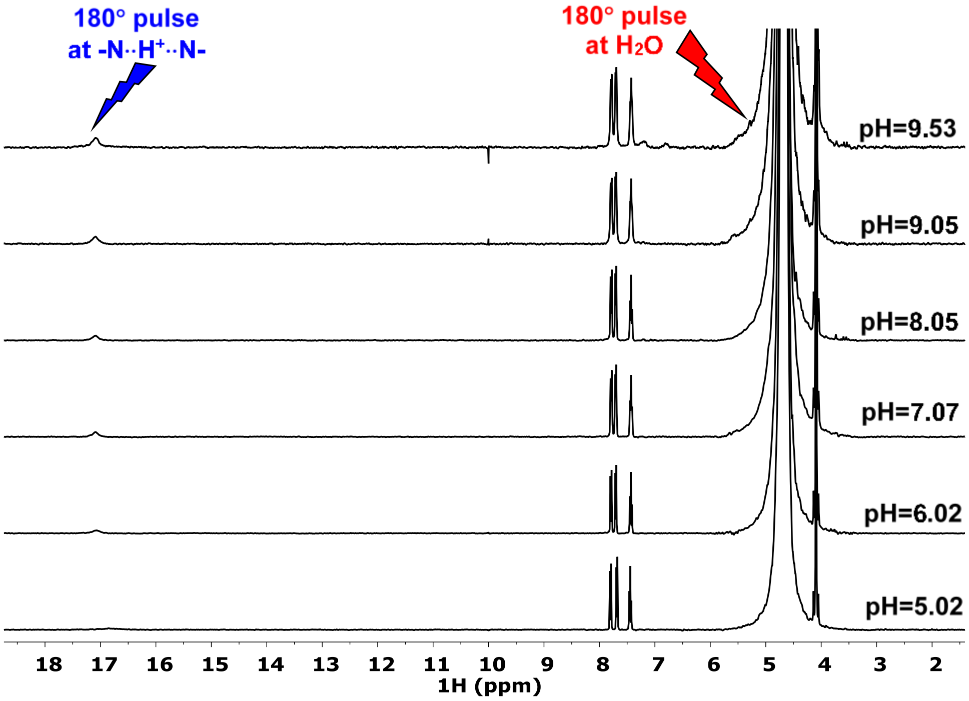

Figure S10. ^{1}H NMR spectra of DANTA at 293 K in 0.15 M NaCl solution ([DANTA]=0.012 M, 9.4 T, H_2O) at various pH values.

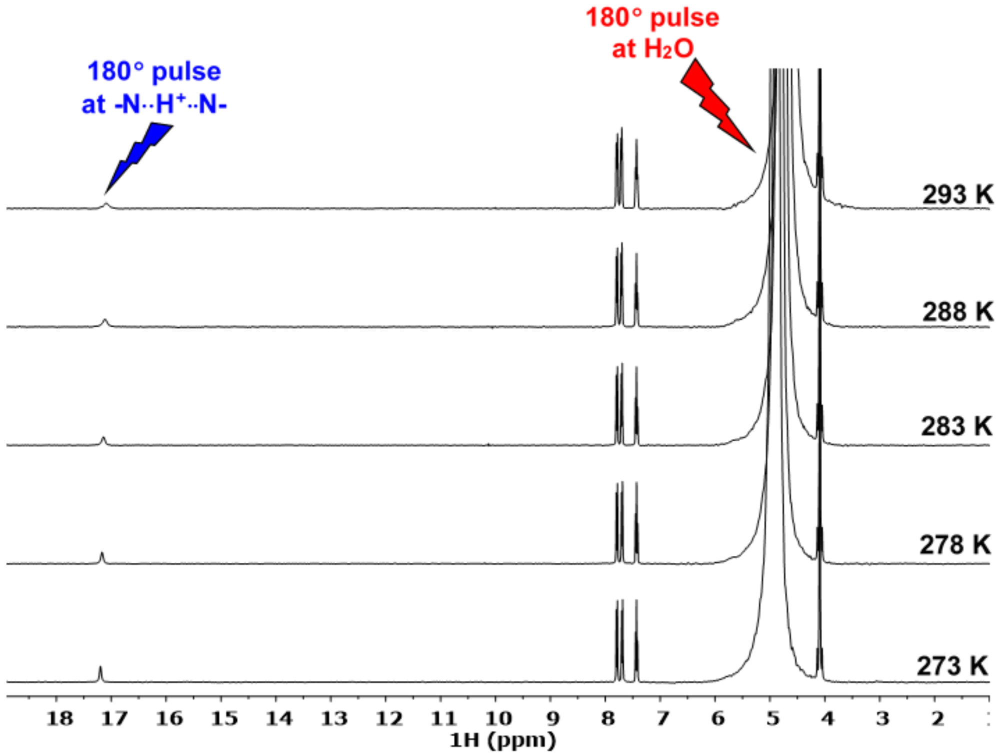

Figure S11. ^{1}H NMR spectra of DANTA at pH=7.07 in 0.15 M NaCl solution ([DANTA]=0.012 M, 9.4 T, H_2O) at varous temperatures.

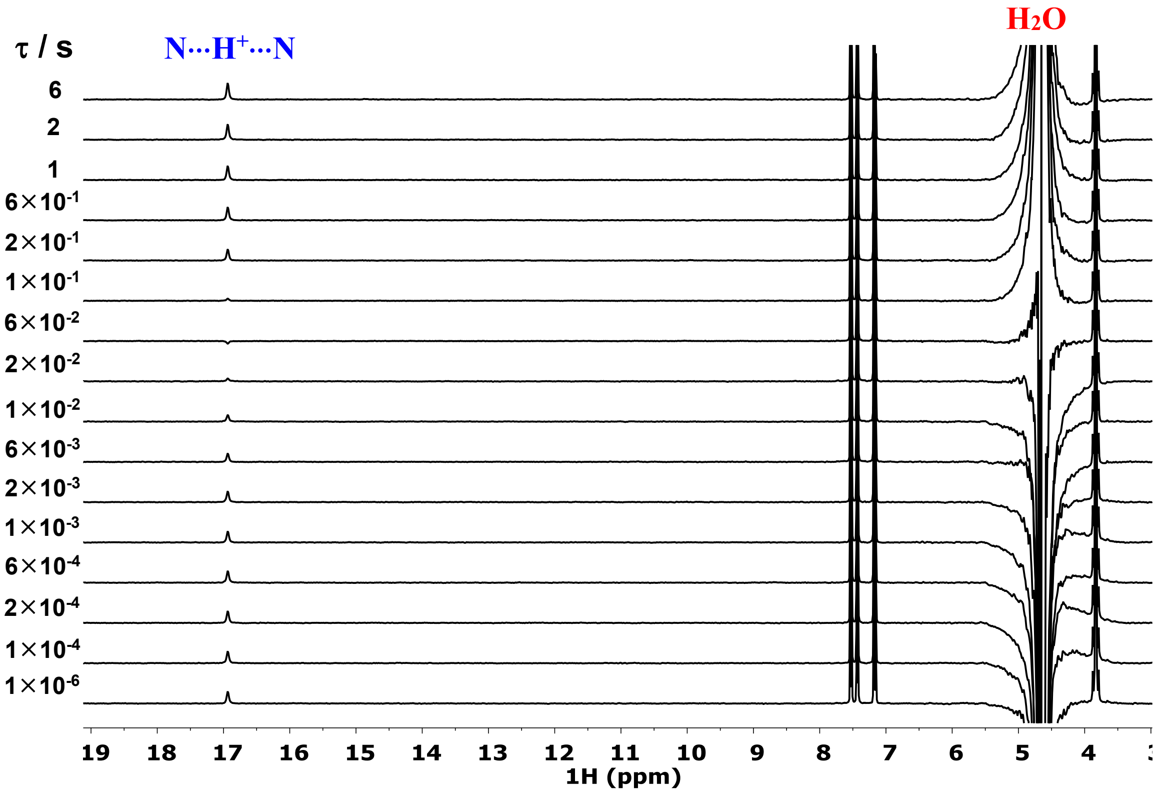

Figure S12. Selective excitation of the H_2O protons at pH=7.07 and 273 K in $HDANTA^{3-}$ solution ([HDANTA]=0.012 M, 9.4 T, 0.15 M NaCl).

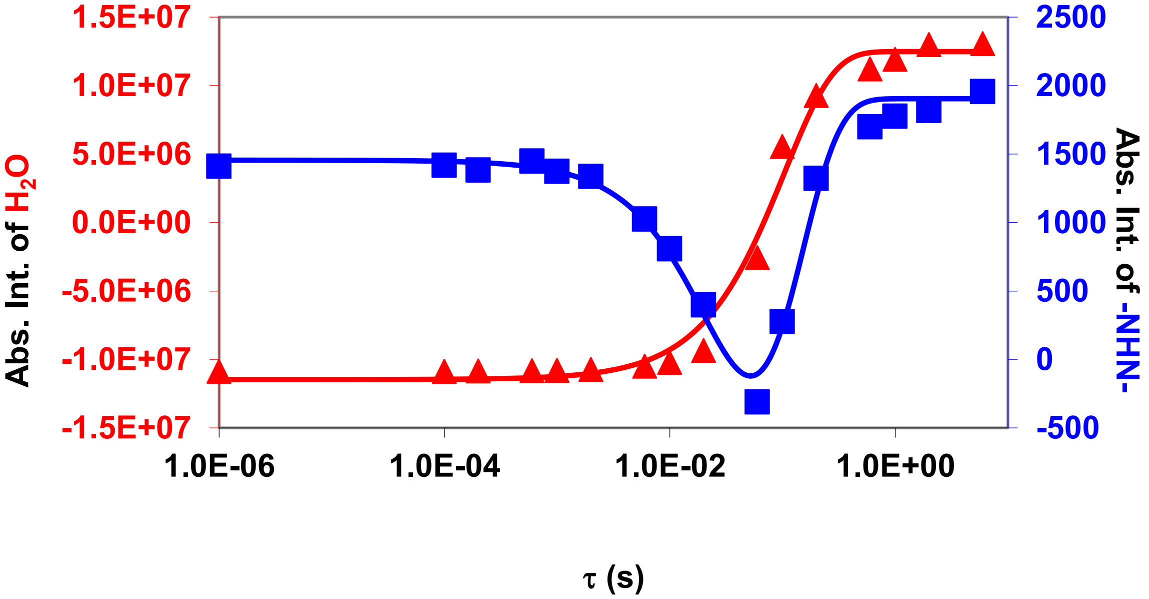

Figure S13. Absolute intensity values of the N···H⁺···N (◆) and H_2O (▲) protons obtained by the selective excitation of the of H_2O proton resonances at pH=7.07 and 273 K in $HDANTA^{3-}$ solution. Symbols and solid lines represent experimental and calculated absolute intensity values, respectively. ([HDANTA]=0.012 M, 9.4 T, 0.15 M NaCl).

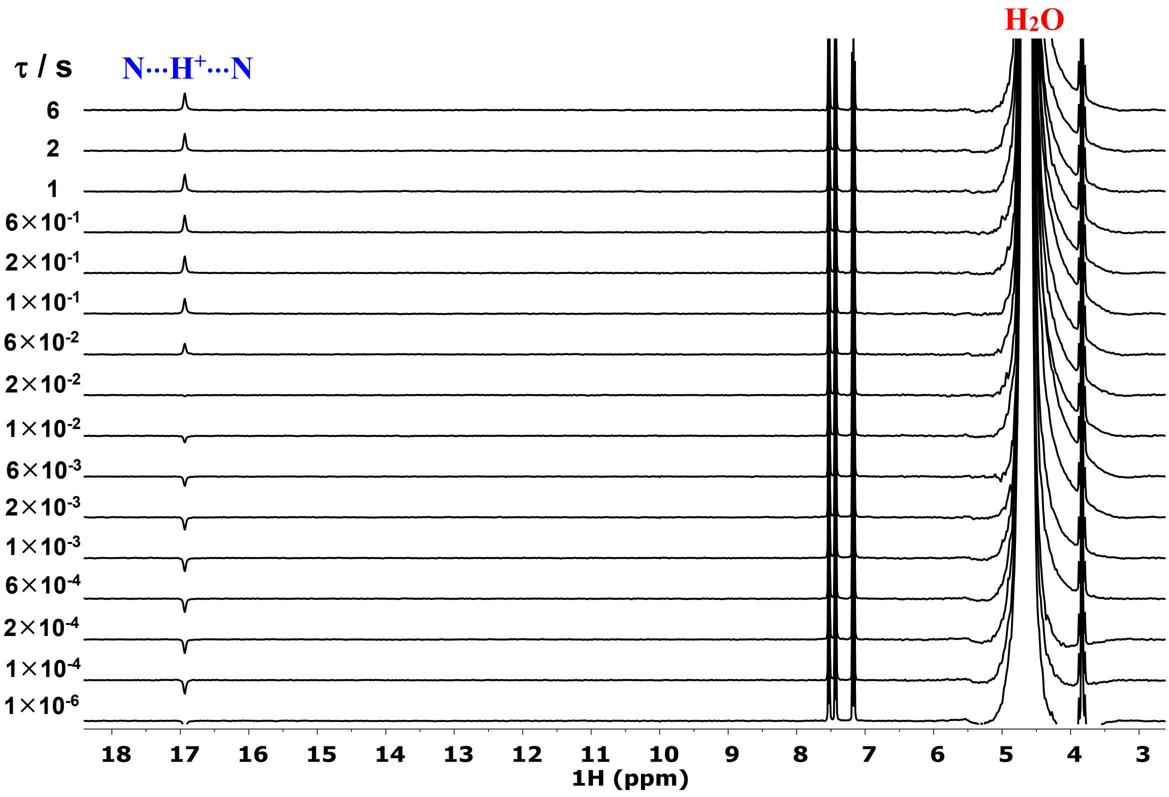

Figure S14. Selective excitation of the N···H$^+$···N proton at pH=7.07 and 273 K in HDANTA^{3-} solution ([HDANTA]=0.012 M, 9.4 T, 0.15 M NaCl).

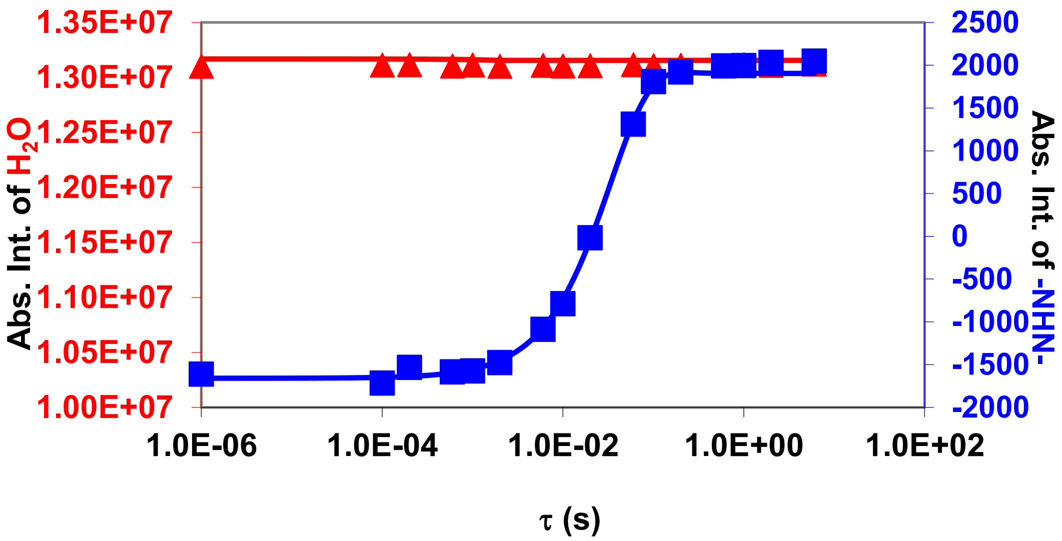

Figure S15. Absolute intensity values of the N···H$^+$···N (◆) and H_2O (▲) protons obtained by the selective excitation of the ^{1}H NMR signals of N···H$^+$···N protons at pH=7.07 and 273 K in HDANTA^{3-} solution. Symbols and solid lines represent experimental and Absolute intensity values, respectively. ([HDANTA]=0.012 M, 9.4 T, 0.15 M NaCl).

Rate of the proton exchange reaction between $HDANTA^{3-}$ and H_2O can be expressed by Eq. (S15).

$$k_{+}[\text{-NHN-}]\times 2[\text{HOH}] = k_{-}[\text{-NHN-}]\times 2[\text{HOH}] \quad \textit{(S15)}$$

where k_+ and k_- are the rate constants characterizing the proton exchange process between the N···H$^+$···N of $HDANTA^{3-}$ and H_2O in a forward and backward directions. In the slow exchange regime of the actual NMR time scale, the ^{1}H NMR signal of the H_2O and N···H$^+$···N of $HDANTA^{3-}$ appear as separate signals in the spectrum. In the selective inversion transfer experiments one of the signals was inverted by a soft 180° pulse, then a non-selective 90° pulse was applied, and the full ^{1}H NMR spectrum was measured after each τ delay time. The exchange phenomenon is described by the Bloch equations:

$$\frac{d[M-M_\infty]}{dt} = R[M - M_\infty] \quad \textit{(S16)}$$

where the z-magnetization is M at time t and M_∞ at time t=∞ ,whereas R is the sum of the k_{ij} rate constant and $1/(T_1)_i$ (where $i \neq j$ and $(T_1)_i$ is the longitudinal relaxation time for the site i). The differential equations describing the distribution of the negative magnetization between the protons of H_2O and N···H$^+$···N in $HDANTA^{3-}$ can be expressed by the following equations:

$$\frac{d[M^{-NHN-} - M_\infty^{-NHN-}]}{dt} = -\left(\left(\frac{1}{T_1^{NHN}}\right) + k_+ \times 2[\mathrm{H_2O}]\right)[M^{NHN} - M_\infty^{NHN}] + k_-[M^{H2O} - M_\infty^{H2O}] \times [\mathrm{DANTA}] \quad \textit{(S17)}$$

$$\frac{d[M^{H2O} - M_\infty^{H2O}]}{dt} = -\left(\left(\frac{1}{T_1^{H2O}}\right) + k_- \times [\mathrm{DANTA}]\right)[M^{H2O} - M_\infty^{H2O}] + k_+[M^{NHN} - M_\infty^{NHN}] \times 2[\mathrm{H_2O}] \quad \textit{(S18)}$$

where M^{-NHN-}, M^{H2O} are the value of magnetization at time t, and $M^{-NHN}{}_\infty$, $M^{H2O}{}_\infty$ at time $t = \infty$. The k_+, k_-, T_1^{NHN} and T_1^{H2O} values have been calculated by the simultaneous fitting the integrals of the deconvoluted N···H$^+$···N of $HDANTA^{3-}$ and H_2O – τ delay time data pairs to Eqs. (S17) and (S18). k_+ and k_- rate constants are shown in Figures S16 and S17. In the temperature range 273 – 293 K the T_1^{H2O} and T_1^{NHN} longitudinal relaxation times of protons in H_2O and N···H$^+$···N proton in $HDANTA^{3-}$ were found to be 0.4 – 1.3 and 0.01-0.005 s, respectively.

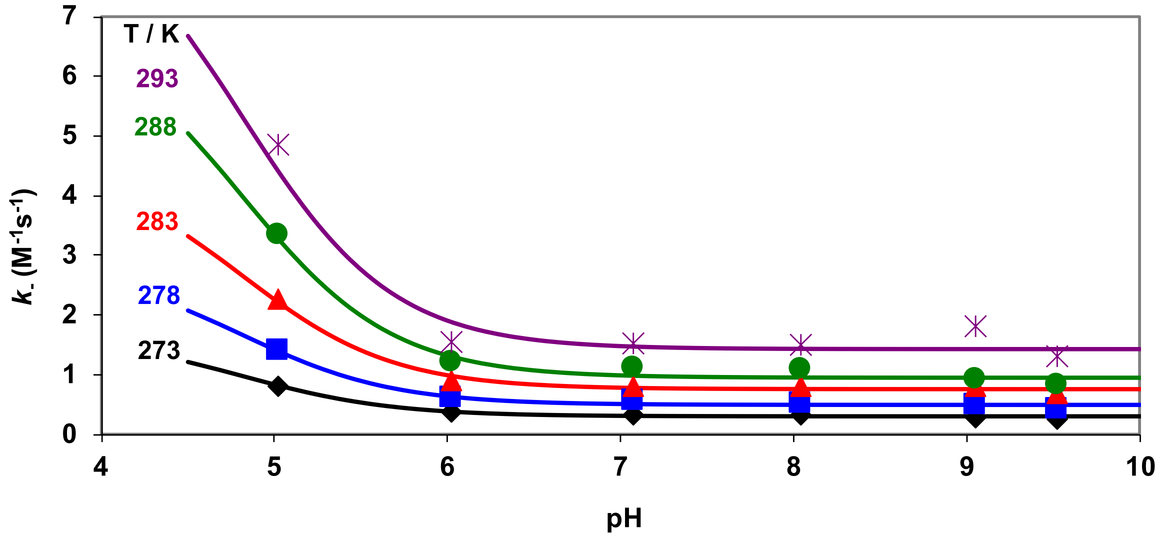

Figure S16. The k_{-} rate constants characterizing the backward exchange rate between **H_2O** and **N···H^{+}···N** protons in HDANTA^{3-} solution. Symbols and solid lines represent experimental and calculated k_{-} values, respectively. ([HDANTA]=0.012 M, 9.4 T, 0.15 M NaCl).

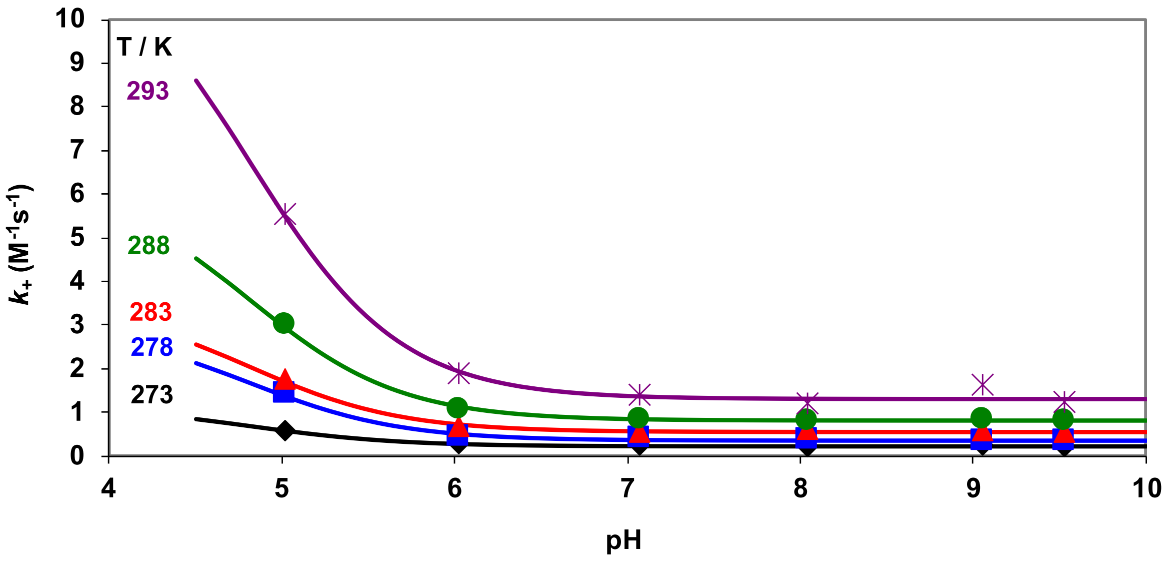

Figure S17. The k_{+} rate constants characterizing the forward exchange rate between **N···H^{+}···N** and **H_2O** protons in HDANTA^{3-} solution. Symbols and solid lines represent experimental and calculated k_{+} values, respectively. ([HDANTA]=0.012 M, 9.4 T, 0.15 M NaCl).

The obtained k_+ and k_- rate constants are independent from the pH in the pH range 6.5 – 9.5, whereas the k_+ and k_- values increase with decreasing pH at pH values <6.0. The pH independent values of k_+ and k_- rate constants in the pH range 6.5 – 9.5 represent the spontaneous proton exchange (k_0) between N···H$^+$···N of $HDANTA^{3-}$ and H_2O (Eq. S19). The dependent k_+ and k_- rate constants represent the proton assisted exchange (k_1) between the N···H$^+$···N of $HDANTA^{3-}$ and H_2O. The proton assisted exchange between the N···H$^+$···N of $HDANTA^{3-}$ and H_2O can be rationalized by the equilibrium formation of a di-protonated H_2DANTA^{2-} (K_2^H, Eq. (20)), which then can exchange the protons with H_2O spontaneously (k_{H2L}, Eq. (S21)).

$$H^*DANTA^{3-} + HOH \xrightarrow{k_0} HDANTA^{+} + HOH^* \qquad (S19)$$

$$HDANTA^{3-} + H^+ \overset{K_2^H}{\rightleftharpoons} H_2DANTA^{2-} \qquad (S20)$$

$$K_2^H = \frac{[H_2DANTA]}{[HDANTA][H^+]}$$

$$H^*HDANTA^{2-} + HOH \xrightarrow{k_{H2L}} H_2DANTA^{2-} + HOH^* \qquad (S21)$$

By taking into account all possible reaction pathways, the total concentration of DANTA ($[DANTA]_{tot}=[HDANTA]+[H_2DANTA]$) and the second protonation constant of DANTA (K_2^H, Eq. S20), k_+ and k_- rate constants can be expressed by Eq. (S22).

$$k_+ = k_- = \frac{k_0 + k_1[H^+]}{1 + K_2^H[H^+]} \qquad (S22)$$

where k_0 and $k_1 = k_{H2L} \times K_2^H$ are the rate constants characterizing the spontaneous and acid catalyzed exchange between the N···H$^+$···N proton of $HDANTA^{3-}$ and H_2O, respectively. k_0 and k_1 rate constants are calculated by fitting the k_+ and k_- - pH data pairs (Figures S16 and S17) to Eq. (S22). k_0 and k_1 rate constants obtained in the temperature range 273 – 298 K are shown in Figure S19. Activation parameters of the proton exchange between N···H$^+$···N of $HDANTA^{3-}$ and H_2O have been calculated by *Eyring* equations (Figure S20). Activation parameters and the

rate constants for the proton exchange of N···H$^+$···N of HDANTA^{3-} and H_2O are summarized in Table 3.

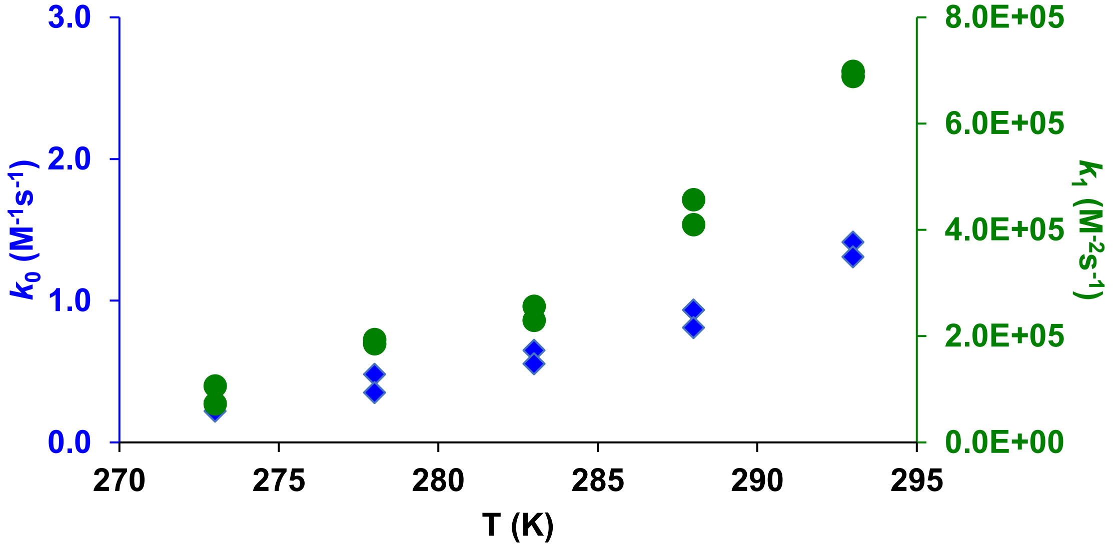

Figure S18. k_0 and k_1 rate constants characterizing the spontaneous and acid catalyzed exchange processes between N···H$^+$···N proton of HDANTA^{3-} and H_2O.

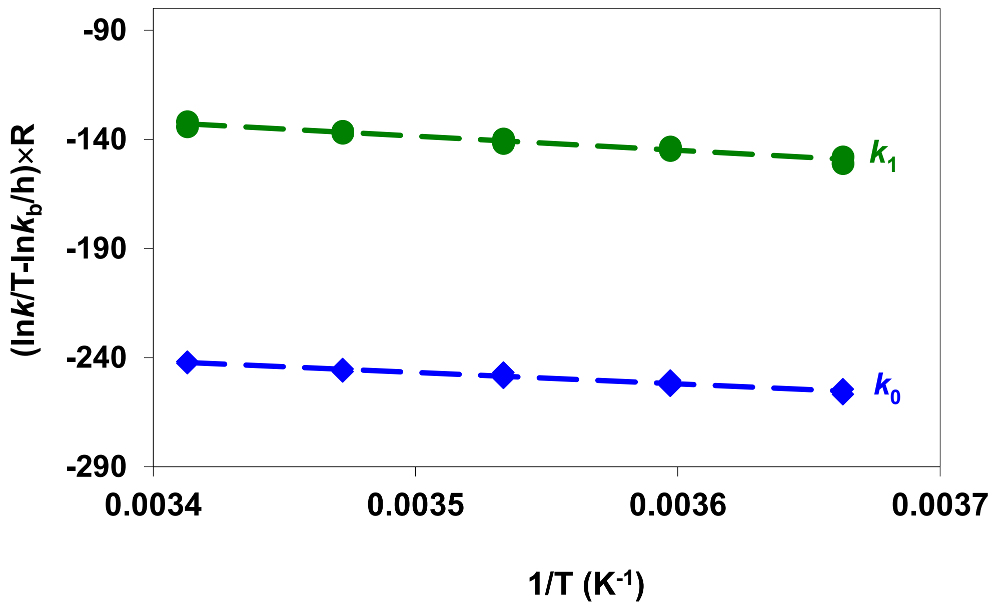

Figure S19. *Eyring* plot for determining the activation parameters of the spontaneous (k_0) and acid catalysed (k_1) proton exchange processes between N···H$^+$···N proton of HDANTA^{3-} and H_2O.

5.3. Proton exchange properties of $HDMAN^+$

The rate of the proton transfer between $HDMAN^+$ and H_2O have been studied by ^1H NMR spectroscopy at pH=7.05 and in the temperature range 293 – 343 K. Typical ^1H NMR spectra of $HDMAN^+$ at different temperatures are shown in Figure S20. ^1H NMR spectra of $HDMAN^+$ indicates that the signal of N···H$^+$···N proton is practically unchanged as the temperature increases from 293 to 343 K at pH=7.05 due to the extremely slow exchange between N···H$^+$···N proton of $HDMAN^+$ and H_2O even at high temperature.

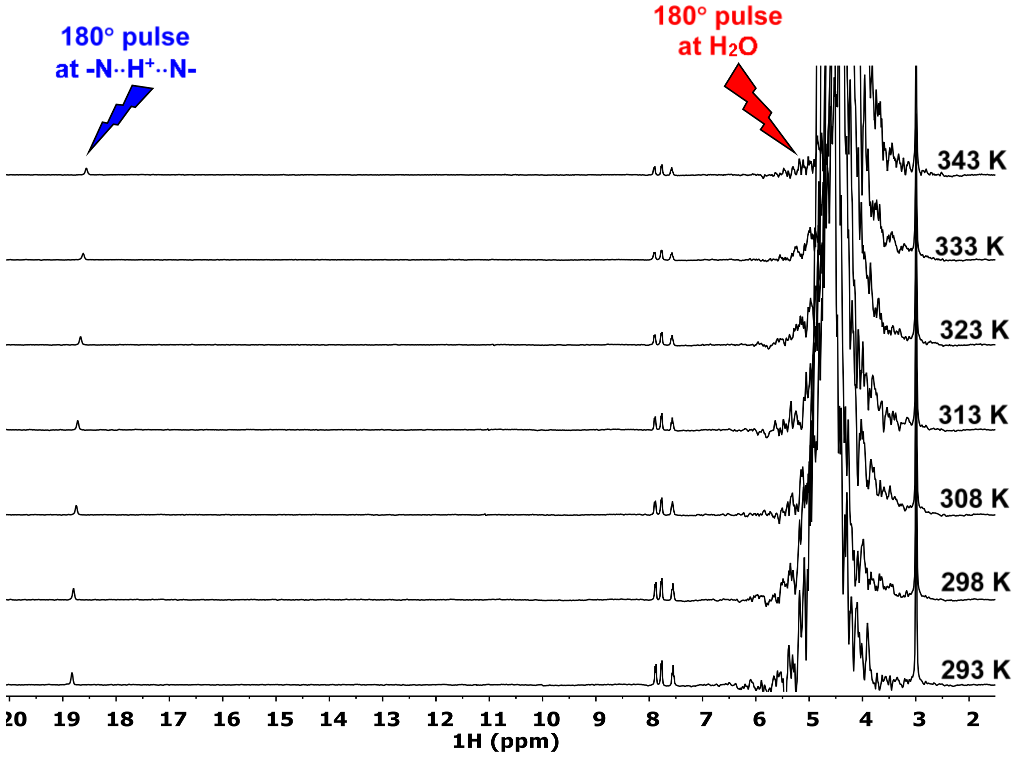

Figure S20. ^1H NMR spectra of $HDMAN^+$ at pH=7.05 in 0.15 M NaCl solution ([DMAN]=0.012 M, 9.4 T, H_2O).

To study the proton exchange between the $HDMAN^+$ and H_2O (Eq. S14) selective inversion transfer experiments were performed by using a DANTE pulse train. ^1H NMR spectra of $HDMAN^+$ obtained by the selective excitation of the H_2O and N···H$^+$···N proton of $HDMAN^+$ at pH=7.05, 298 and 323 K are shown in Figures S21, S23, S25 and S27, respectively. Absolute integral values of the H_2O and N···H$^+$···N proton of $HDMAN^+$ as a function of τ delay time obtained at pH=7.05, 298 and 323 K are shown in Figures S22, S24, S26 and S28.

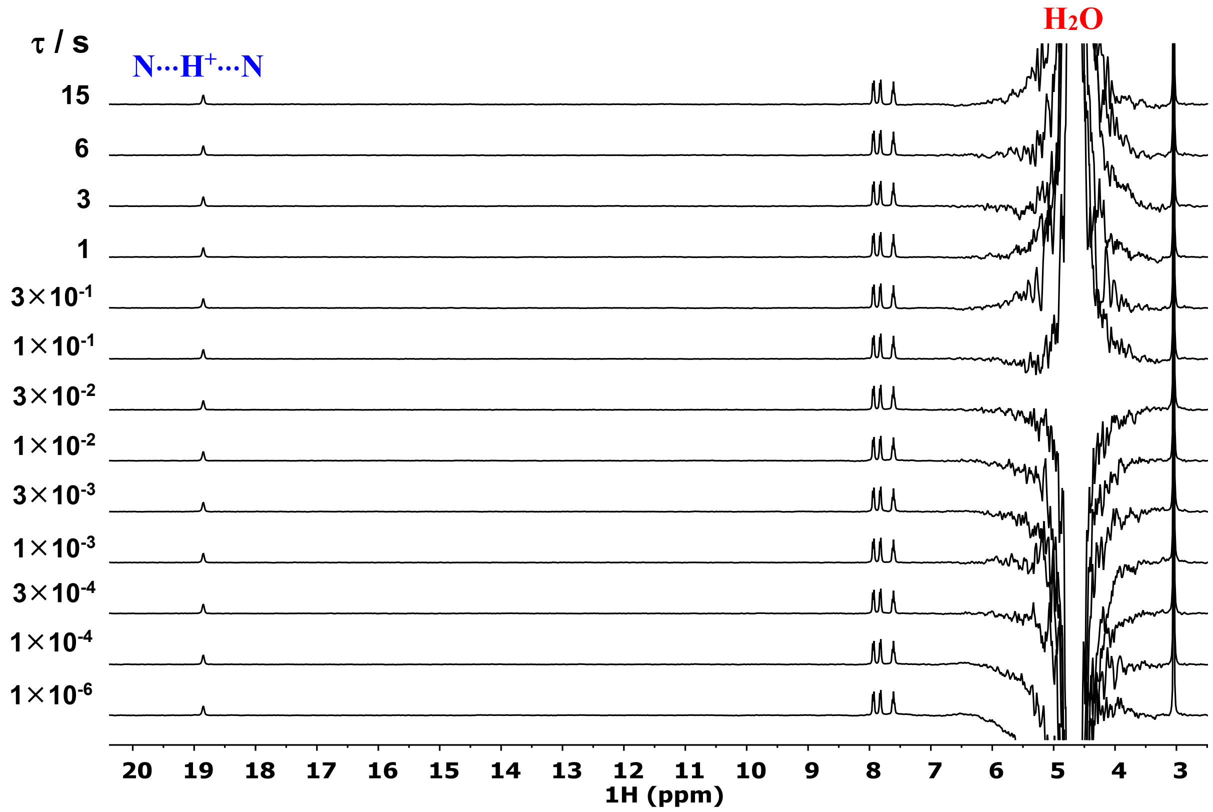

Figure S21. Selective excitation of the H_2O protons at pH=7.05 and 298 K in HDMAN$^+$ solution ([DMAN]=0.012 M, 9.4 T, 0.15 M NaCl)

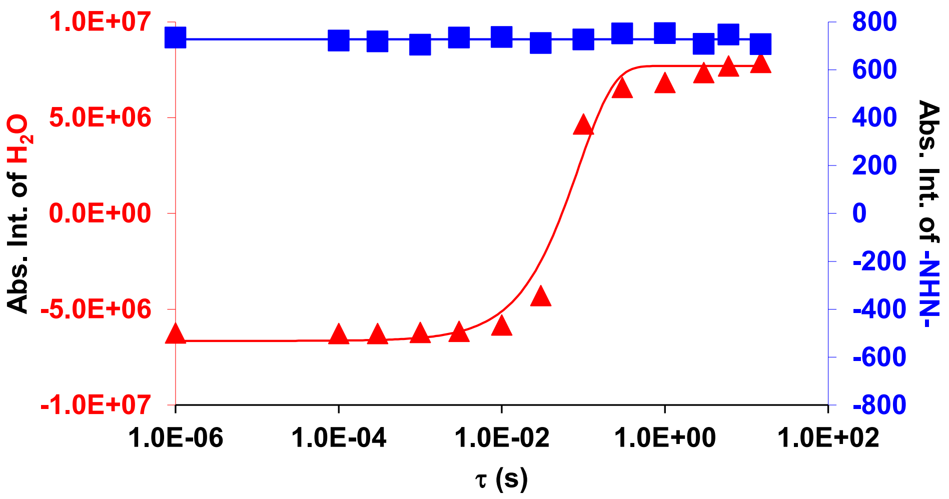

Figure S22. Absolute intensity values of the N···H$^+$···N (◆) and H_2O (▲) proton signals obtained by the selective excitation of the ^{1}H NMR signals of H_2O proton resonance at pH=7.05 and 298 K in HDMAN$^+$ solution. Symbols and solid lines represent experimental and calculated Absolute intensity values, respectively. ([DMAN]=0.012 M, 9.4 T, 0.15 M NaCl).

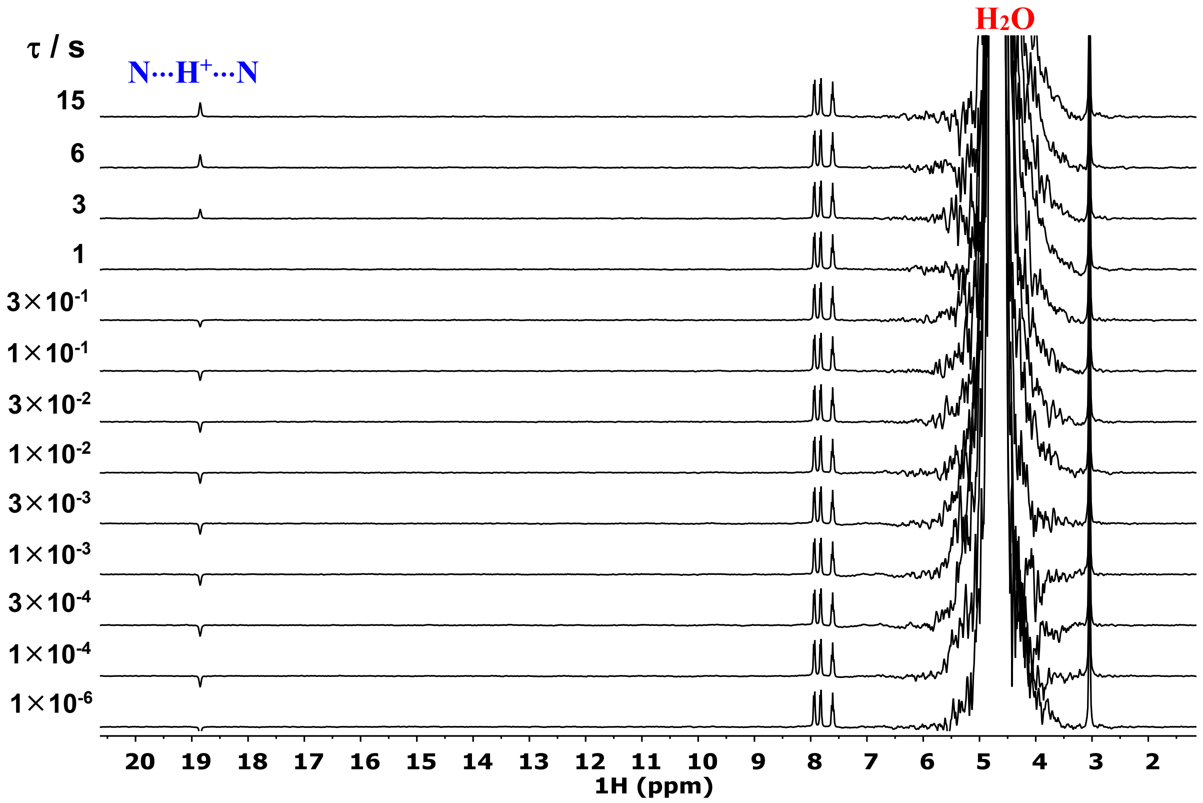

Figure S23. Selective excitation of the N···H$^+$···N proton at pH=7.05 and 298 K in HDMAN$^+$ solution ([DMAN]=0.012 M, 9.4 T, 0.15 M NaCl).

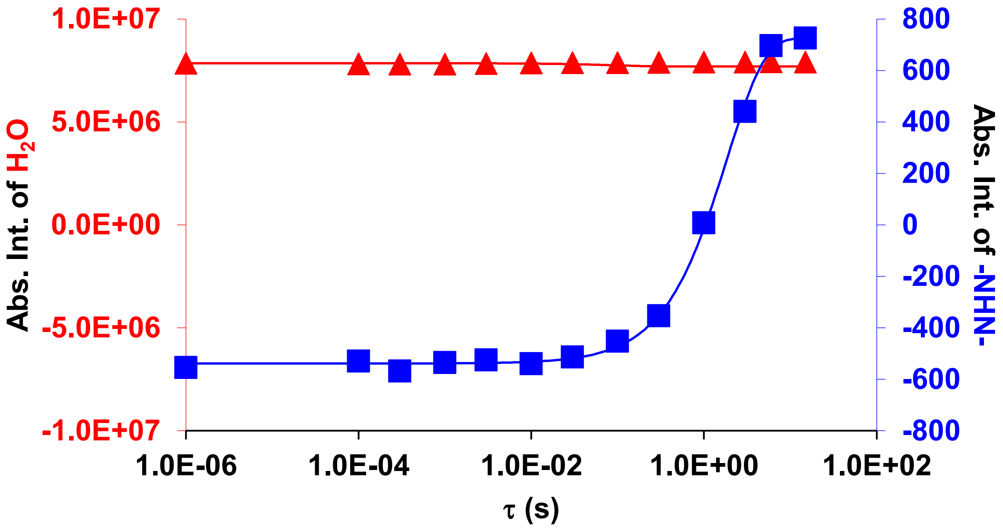

Figure S24. Absolute intensity values of the N···H$^+$···N (◆) and H_2O (▲) proton signals obtained by the selective excitation of the ^{1}H NMR signals of N···H$^+$···N proton resonance at pH=7.05 and 298 K in HDMAN$^+$ solution. Symbols and solid lines represent experimental and calculated abs. int. values, respectively. ([DMAN]=0.012 M, 9.4 T, 0.15 M NaCl).

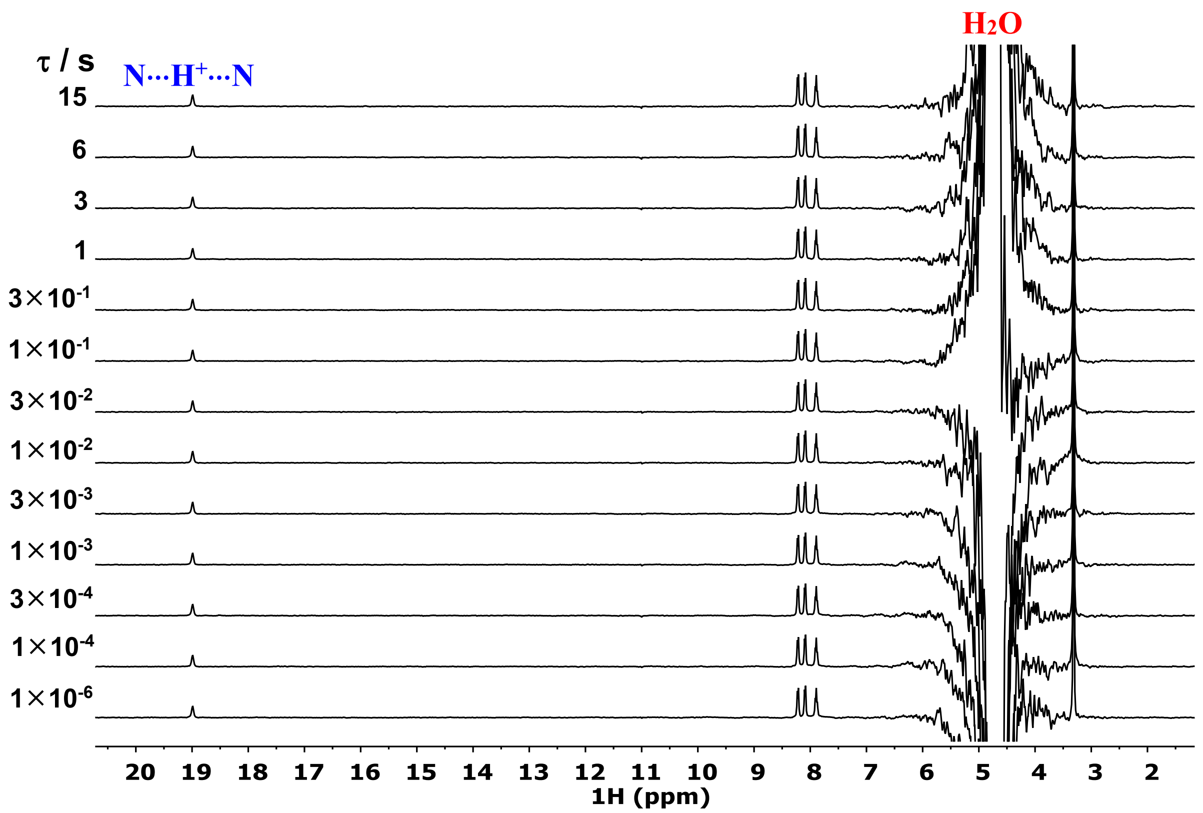

Figure S25. Selective excitation of the H_2O protons at pH=7.05 and 323 K in $HDMAN^+$ solution ([DMAN]=0.012 M, 9.4 T, 0.15 M NaCl).

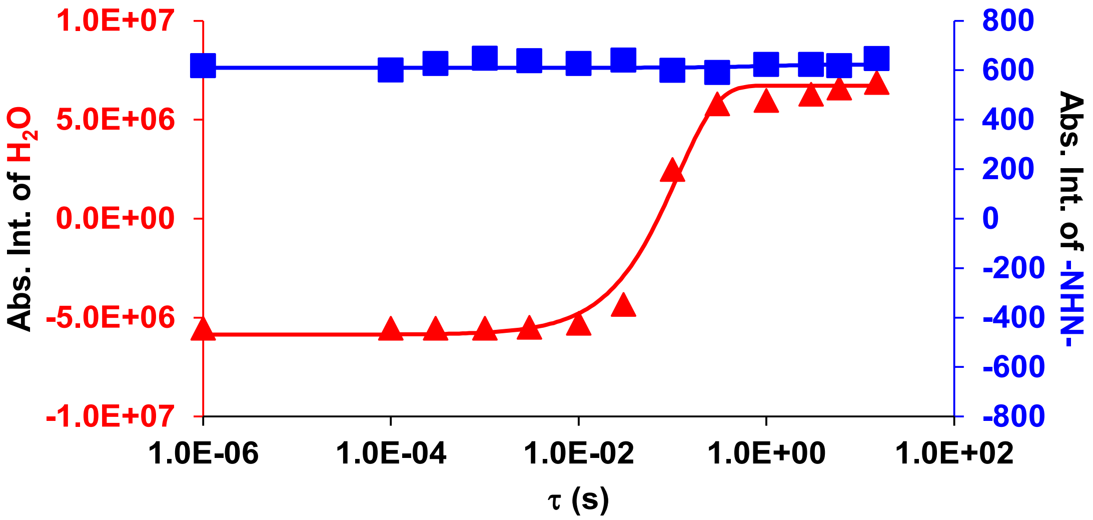

Figure S26. Absolute intensity values of the N···H$^+$···N (◆) and H_2O (▲) proton signals obtained by the selective excitation of H_2O proton resonance at pH=7.05 and 323 K in $HDMAN^+$ solution. Symbols and solid lines represent experimental and calculated absolute intensity values, respectively. ([DMAN]=0.012 M, 9.4 T, 0.15 M NaCl).

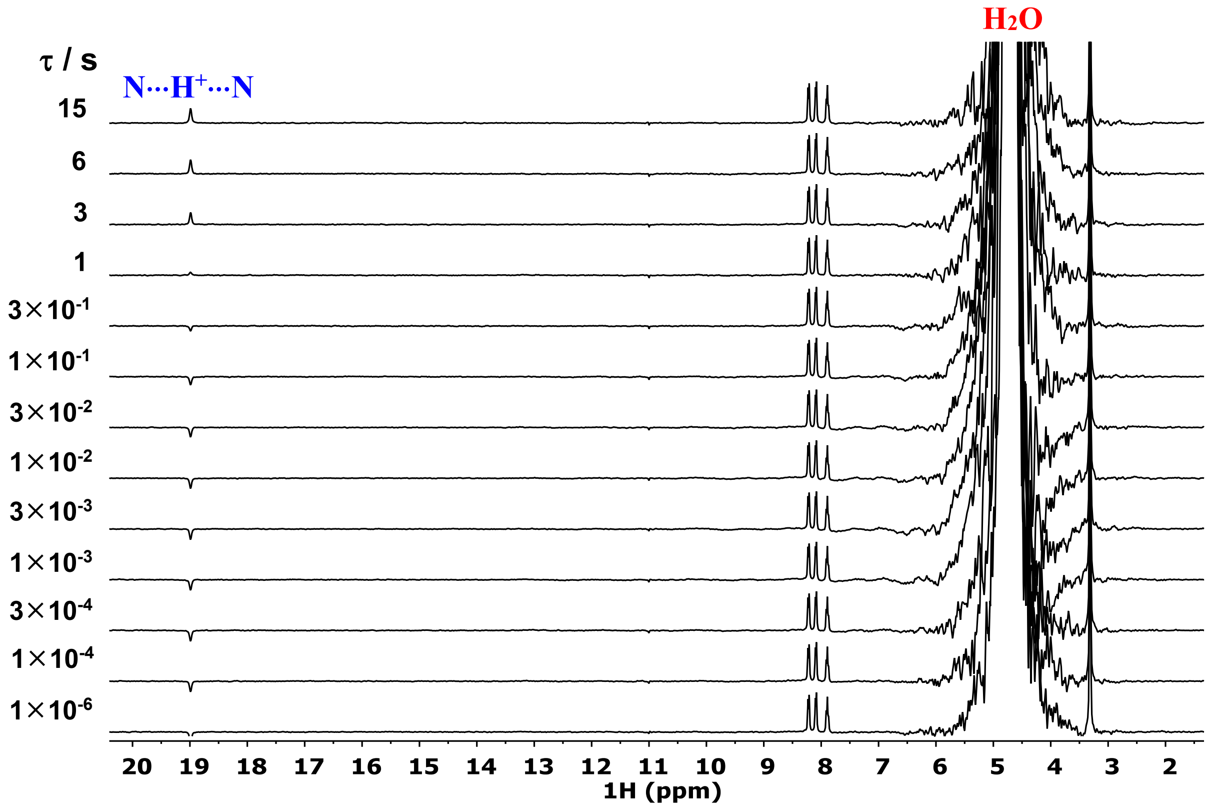

Figure S27. Selective excitation of the N···H$^+$···N proton at pH=7.05 and 323 K in HDMAN$^+$ solution ([DMAN]=0.012 M, 9.4 T, 0.15 M NaCl).

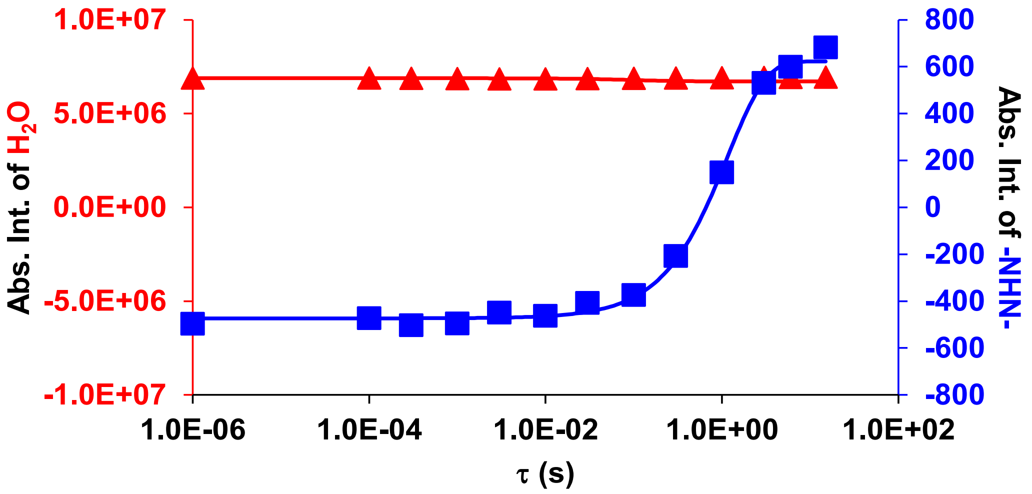

Figure S28. Abs. int. values of the N···H$^+$···N (◆) and H_2O (▲) protons obtained by the selective excitation of the ^{1}H NMR signals of N···H$^+$···N proton resonance at pH=7.05 and 323 K in HDMAN$^+$ solution. Symbols and solid lines represent experimental and calculated abs. int. values, respectively ([DMAN]=0.012 M, 9.4 T, 0.15 M NaCl).

5.4. Proton exchange properties of salicylate

The rate of the proton transfer between phenolate-OH of salicylate and H_2O have been studied by ^1H NMR spectroscopy at 298 K and in the pH range 6.58 – 7.98. Typical ^1H NMR spectra of salicylate at different pH values are shown in Figure S29. ^1H NMR spectra of salicylate indicates that the signal of the phenolate-OH proton is broaden with the decrease of the pH at pH<7.89 due to the increase of the exchange rate between phenolate-OH proton of salicylate and H_2O even at room temperature.

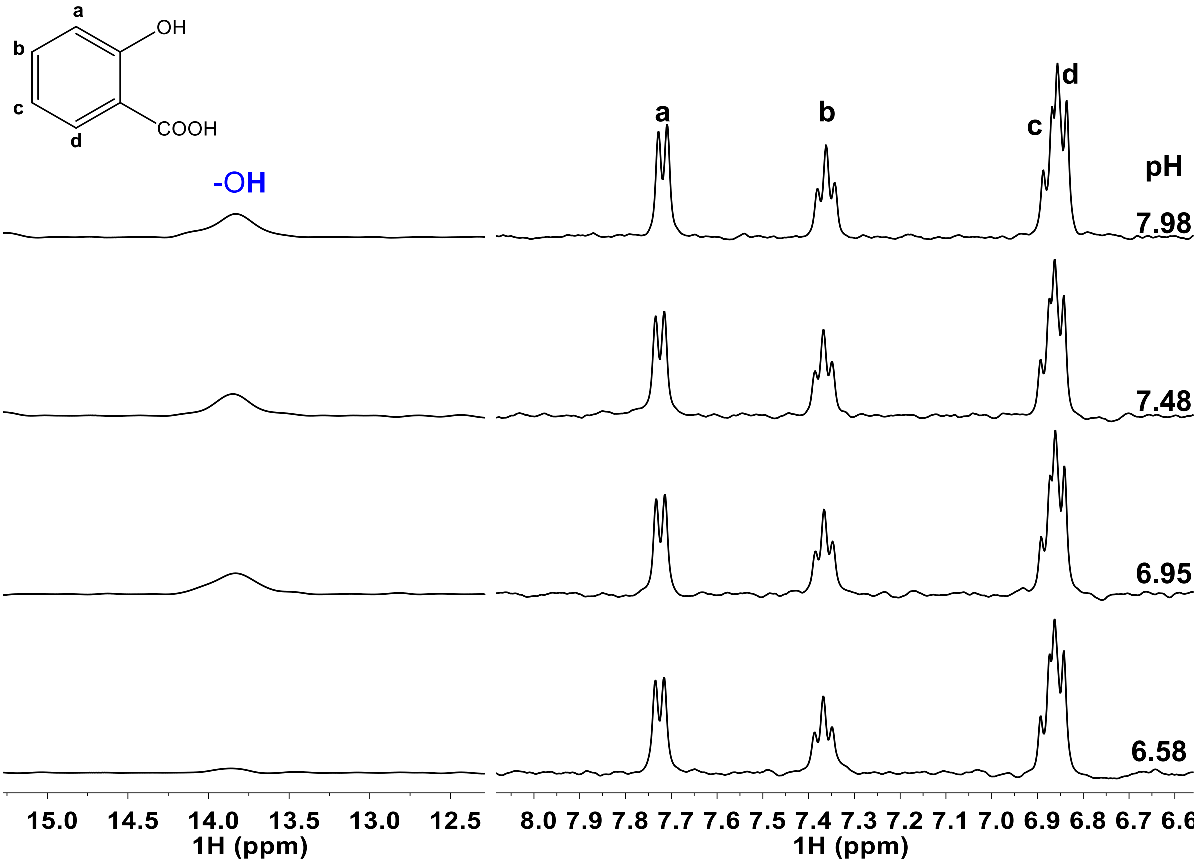

Figure S29. ^1H NMR spectra of salicylic acid as a function of pH ([salicylic acid]=12 mM, 9.4 T, 298 K, H_2O, 0.15 M NaCl).

To study the proton exchange between the phenolate-OH of salicylate and H_2O (Eq. S23) selective inversion transfer experiments were performed by using a DANTE pulse train. ^1H NMR spectra of salicylate obtained by the selective excitation of the H_2O and phenolate-OH proton of salicylate at pH=8.29 and 298 are shown in Figures S30 and S32, respectively. Absolute integral values of the H_2O and phenolate-OH proton of salicylate as a function of τ delay time obtained at pH=8.29 and 298 K are shown in Figures S31 and S33.

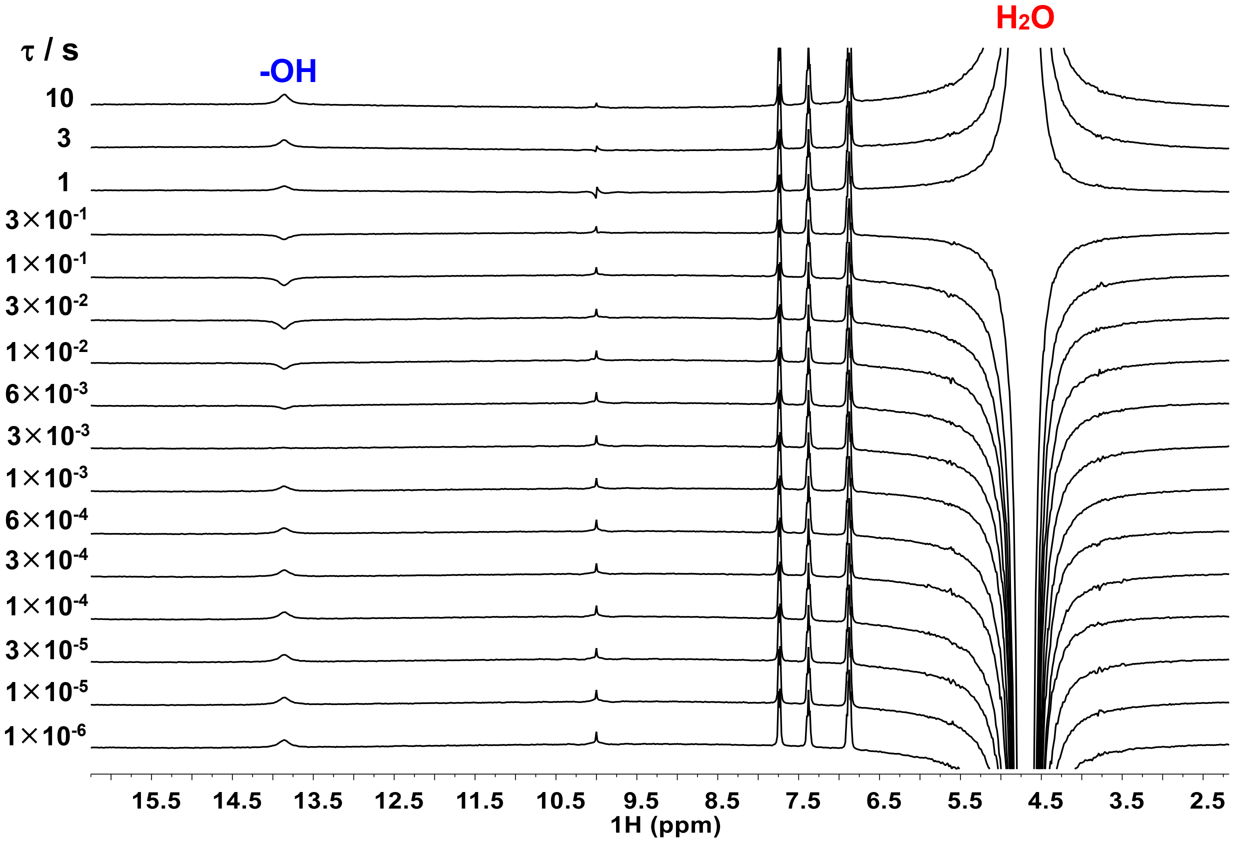

Figure S30. Selective excitation of the H_2O protons at pH=8.29 and 298 K in salicylate solution ([salicylate]=0.012 M, 9.4 T, 0.15 M NaCl).

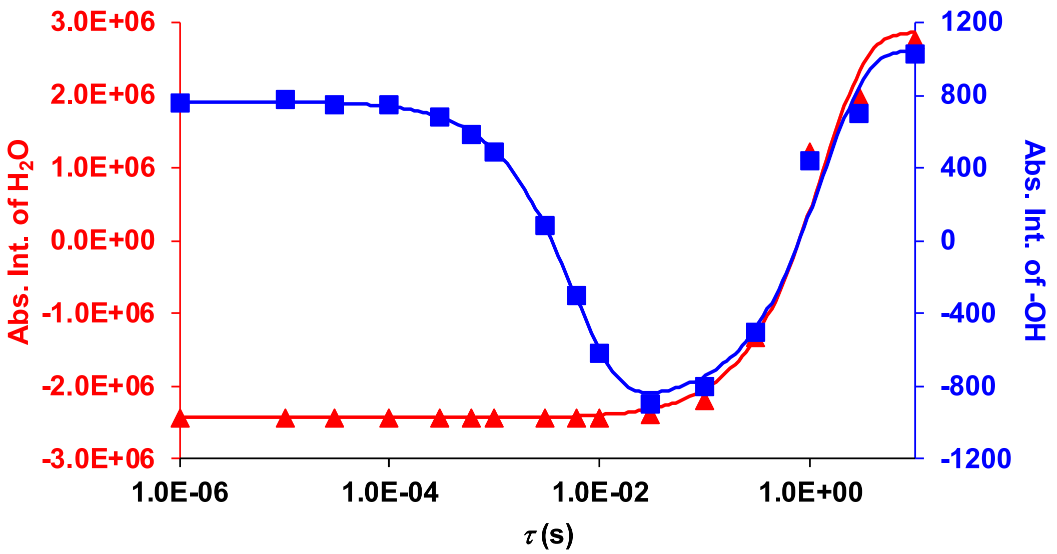

Figure S31. Abs. int. values of the -OH (◆) and H_2O (▲) protons obtained by the selective excitation of the ^{1}H NMR signals of H_2O protons at pH=8.29 and 298 K in salicylate solution. Symbols and solid lines represent experimental and calculated abs. int. values, respectively. ([salicylate]=0.012 M, 9.4 T, 0.15 M NaCl).

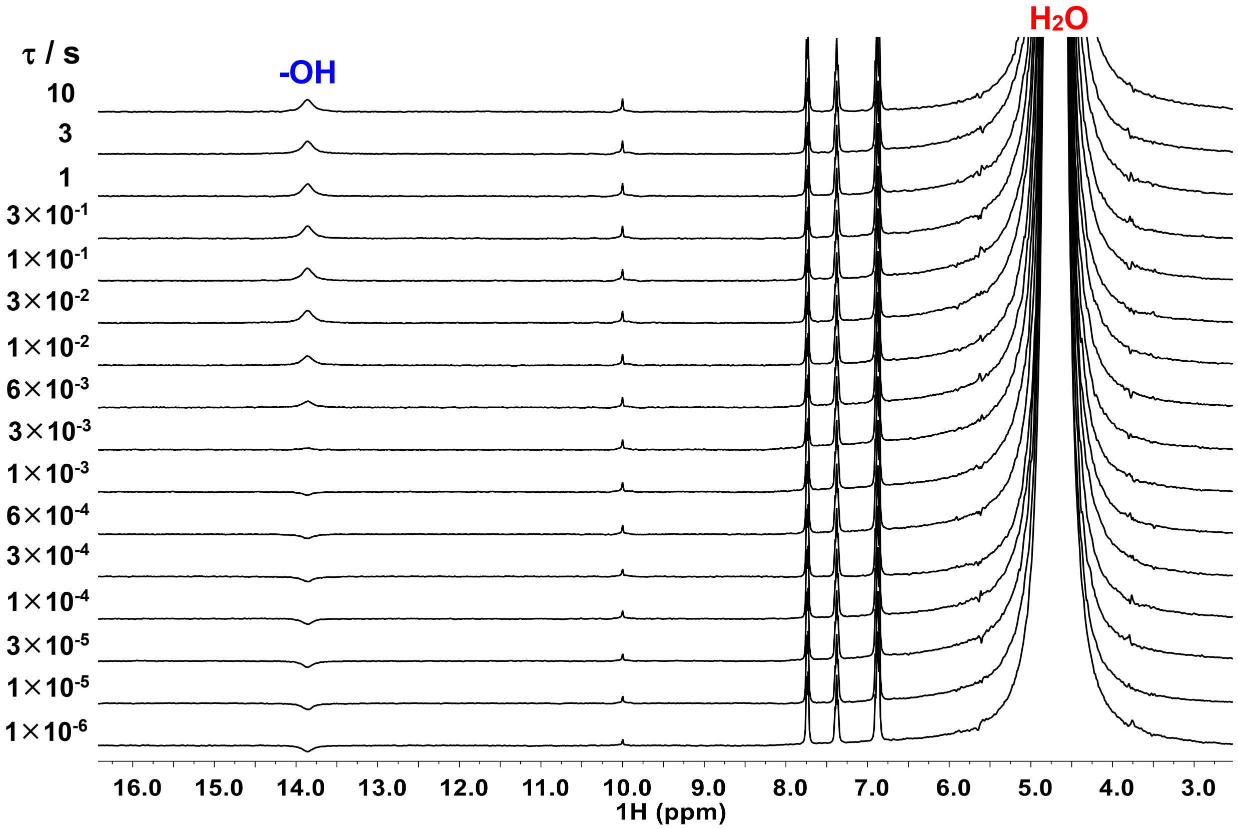

Figure S32. Selective excitation of the **-OH** protons at pH=8.29 and 298 K in salicylate solution ([salicylate]=0.012 M, 9.4 T, 0.15 M NaCl).

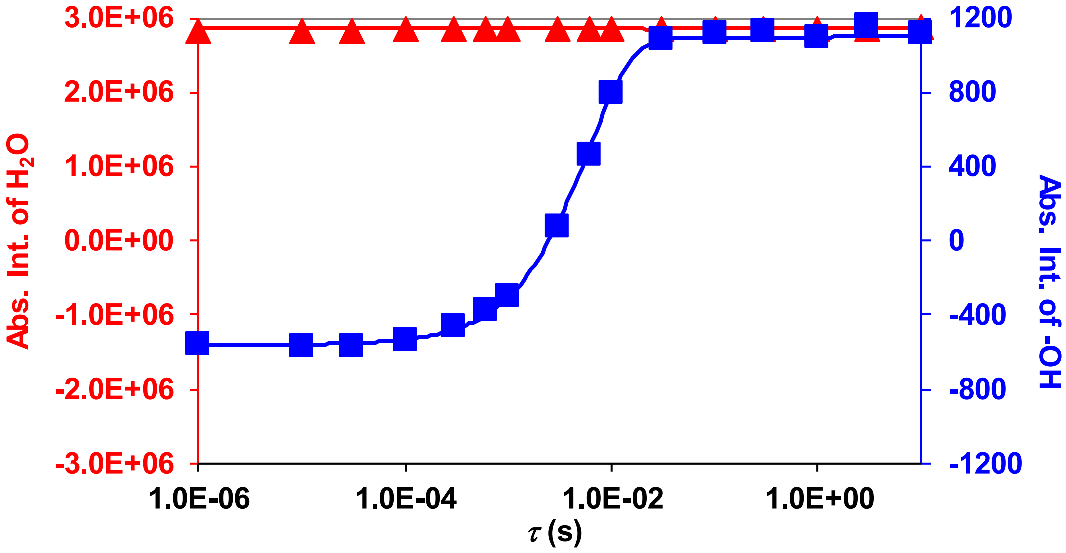

Figure S33. Abs. int. values of the **-OH** (◆) and **H_2O** (▲) protons obtained by the selective excitation of the ^{1}H NMR signals of **-OH** proton at pH=8.29 and 298 K in salicylate solution. Symbols and solid lines represent experimental and calculated absolute intensity values, respectively. ([salicylate]=0.012 M, 9.4 T, 0.15 M NaCl).

$$-OH^* + HOH \underset{v_-}{\overset{v_+}{\rightleftharpoons}} -OH + H^*OH \qquad (S23)$$

Rate of the proton exchange reaction between -OH of salicylate and H_2O can be expressed by Eq. (S24).

$$k_+[-OH]\times 2[HOH] = k_-[-OH]\times 2[HOH] \qquad (S24)$$

where k_+ and k_- are the rate constants characterizing the proton exchange process between the -OH of salicylate and H_2O in a forward and backward directions. The k_+ and k_- rate constants and T_1^{OH} and T_1^{H2O} longitudinal relaxation time of the -OH and H_2O protons have been calculated by the simultaneous fitting the integrals of the deconvoluted -OH of salicylate and $H_2O - \tau$ delay time data pairs to Eqs. (S17) and (S18). k_+ and k_- rate constants are shown in Figure S34. At 298 K the T_1^{H2O} and T_1^{OH} longitudinal relaxation times of protons in H_2O and -OH proton in salicylate were found to be 1.33 and 1.28 s, respectively.

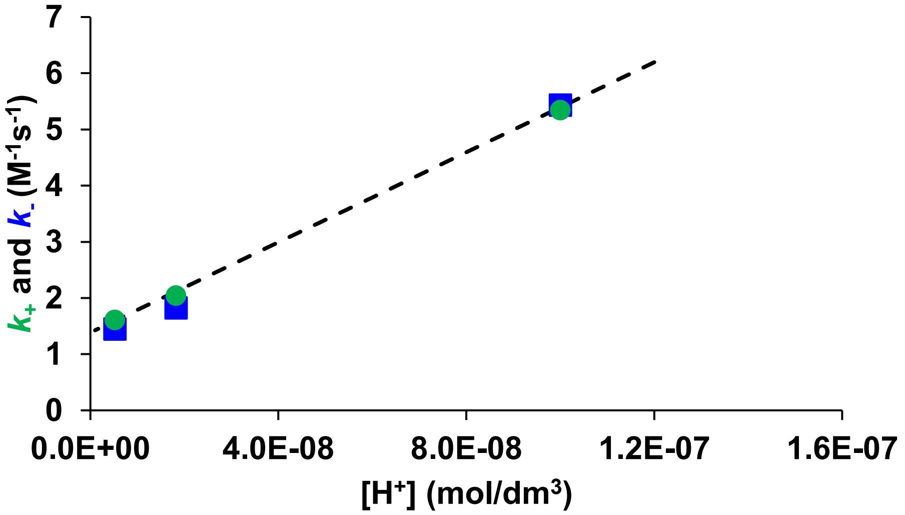

Figure S34. k_+ and k_- rate constants characterizing the exchange between **H_2O** and **-OH** protons in salicylate solution (298 K, 0.15 M NaCl). Symbols and dashed line represent experimental and calculated rate constants, respectively.

Based on the dependence of the obtained k_+ and k_- rate constants on $[H^+]$ (Figure S34), it can be assumed that the proton exchange between -OH of salicylate and H_2O occurs via a spontaneous and an acid catalyzed (proton assisted) pathway (Eq. S25 and S26) characterized with k_0 and k_1 rate constant, respectively. The increase of k_+ and k_- rate constants with the increase of $[H^+]$ can be explained by the increase of the proton assisted exchange rate (Eq. (S26)) characterized with k_1 rate constant.

k_0

$$\text{-OH* + HOH} \longrightarrow \text{-OH + HOH*} \qquad (S25)$$

$$\text{-OH* + [H}_2\text{OH]}^+ \xrightarrow{k_1} \text{-OH + [H}_2\text{OH*]}^+ \qquad (S26)$$

By taking into account all possible reaction pathways, k_+ and k_- rate constants can be expressed by Eq. (S27).

$$k_+ = k_- = k_0 + k_1[\mathrm{H}^+] \qquad (S27)$$

where k_0 and k_1 are the rate constants characterizing the spontaneous and acid catalyzed exchange between the phenolate-OH proton of salicylate and H_2O, respectively. k_0 and k_1 rate constants are calculated by fitting the k_+ and k_- - pH data pairs (Figure S34) to Eq. (S27). k_0 and k_1 rate constants characterizing the spontaneous and acid catalyzed exchange between the phenolate-OH proton of salicylate and H_2O were found to be k_0=1.38 ± 0.08 $M^{-1}s^{-1}$; k_1=(4.0 ± 0.4)×10^7 $M^{-2}s^{-1}$ at 298 K in 0.15 M NaCl. Considering the k_0 and k_1 rate constants, the k_{ex} pseudo-first-order rate constant characterizing the exchange between the phenolate-OH proton of salicylate and H_2O in a forward direction can be calculated at pH=7.4 and 298K as k_{ex}^{298}= $k_{+/-}^{298}$×2[H_2O]=(k_0 + k_1[H^+])×2[H_2O] =330 s^{-1}.

6. X-ray diffraction studies of $HDANTA^{3-}$

6.1. Experimental

Single crystals with the composition of [Na_3(HDANTA)]·EtOH·20H_2O have been obtained with the slow diffusion of EtOH and Et_2O mixture into 0.5 mL aqueous solutions of [Na_3(HDANTA)] prepared by the dissolution of 0.1 mmol H_4DANTA and 0.3 mmol NaOH (4°C). The pH of the [Na_3(HDANTA)] solution was 8.0. The crystal was fixed on a Hampton Research loop using high viscosity oil (Figure S30). Data were collected at 150 K or room temperature using a Bruker-D8 Venture diffractometer equipped with INCOATEC IμS 3.0 dual (Mo) sealed tube microsources and Photon 200 Charge-integrating Pixel Array detector. Several datasets were collected and the most reliable was used for each compound. Data collection and integration were performed using the APEX4 software (*APEX4* v2017.3-0, Bruker AXS Inc.). Data reduction and multi-scan absorption correction was applied (*SAINT* V8.38A and *SADABS2016*/2, Bruker AXS Inc) and numerical absorption correction did not improve the refinement. The structures could be solved using direct methods and refined on F^2 using SHELXL program[22] incorporated into the APEX4 suite. The statistics of the reflections as well as systematic absences indicated and supported the suggested space group. Finally, the

PLUTON crystallographic software did not report any missed symmetry elements. Refinement was performed anisotropicaly for all non-hydrogen atoms. Hydrogens were placed into geometric positions except O-H or N-H protons which usually could be found at the electron density map but the respective O-H or N-H distances were usually constrained. Tables were extracted from the edited CIF file using publCIF.[23] The PLATON program[24,25] was used for crystallographic calculations. The structural data are reasonable according to printed compilation[26] of bond distance data and stand-alone checking features of the PLATON[24,25] crystallographic software. There are several A and B level errors in the check if files and these errors can be explained by the uncertain orientation of water molecules or shape and quality of the crystals resulting in irregular ADP values, short H..H and D..A contacts and significant shift in the final refinement in some cases. The A Level errors are explained in the cif and checkcif files in Validation Reply Forms. Figures were prepared using Ortep-3,[27] CCDC Mercury[28] and PyMOL[29] software. CCDC 2541016 contain the supplementary crystallographic data for $[Na_3(HDANTA)]\cdot EtOH\cdot 20H_2O$. Related files can be obtained free of charge from The Cambridge Crystallographic Data Centre via https://www.ccdc.cam.ac.uk/structures.

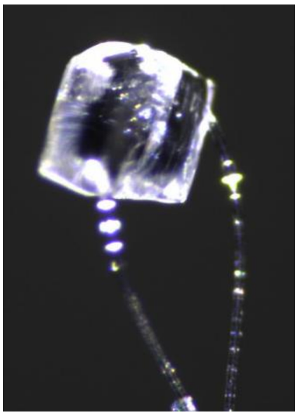

Figure S35. A single crystal of $[Na_3(HDANTA)]\cdot EtOH\cdot 20H_2O$ on a Hampton Research loop mounted on a goniometer head.

6.2. X-ray structure of the mono-protonated HDANTA^{3-}

Details of structure determination are shown in Table S4. The X-ray structure, the selected bond distances and the hydrogen bond parameters of mono-protonated HDANTA^{3-} are shown in Figures S36 and S37, Tables S5 and S6.

Table S4. Experimental data of X-ray structure determination of $[Na_3(HDANTA)]·EtOH·20H_2O$.

**Crystal data $[Na_3(HDANTA)]·EtOH·20H_2O$**	
**Chemical formula**	$(C_{18}H_{15}N_4Na_3O_8)_2.C_2H_6O·20(H_2O)$
**Sum formula**	C_{38} H_{76} N_4 Na_6 O_{37}
M_r	1318.96
**Crystal system, space group**	Triclinic, $P\bar{1}$
**Temperature (K)**	150
***a*, *b*, *c* (Å)**	8.5334 (9), 11.2573 (13), 31.185 (4)
**α, β, γ (°)**	80.561 (4), 82.453 (5), 86.550 (4)
V **(Å^3)**	2927.3 (6)
Z	2
**Radiation type**	Mo $K\alpha$
**μ (mm^{-1})**	0.17
**Crystal size (mm)**	0.55 × 0.48 × 0.27
**Data collection**	
**Diffractometer**	Bruker D8 VENTURE
**Absorption correction**	Multi-scan<br>*SADABS2016*/2 - Bruker AXS area detector scaling and absorption correction
T_{min}, T_{max}	0.64, 0.95
**No. of measured, independent and observed [$I > 2\sigma(I)$] reflections**	50102, 10991, 8677
R_{int}	0.065
**$(\sin \theta/\lambda)_{max}$ (Å^{-1})**	0.618
**Refinement**	
$R[F^2 > 2\sigma(F^2)]$, $wR(F^2)$, S	0.081, 0.244, 1.02
**No. of reflections**	10991
**No. of parameters**	897
**No. of restraints**	47
**H-atom treatment**	H atoms treated by a mixture of independent and constrained refinement
$(\Delta/\sigma)_{max}$	6.358
$\Delta\rangle_{max}$, $\Delta\rangle_{min}$ **(e Å^{-3})**	2.28, -0.73

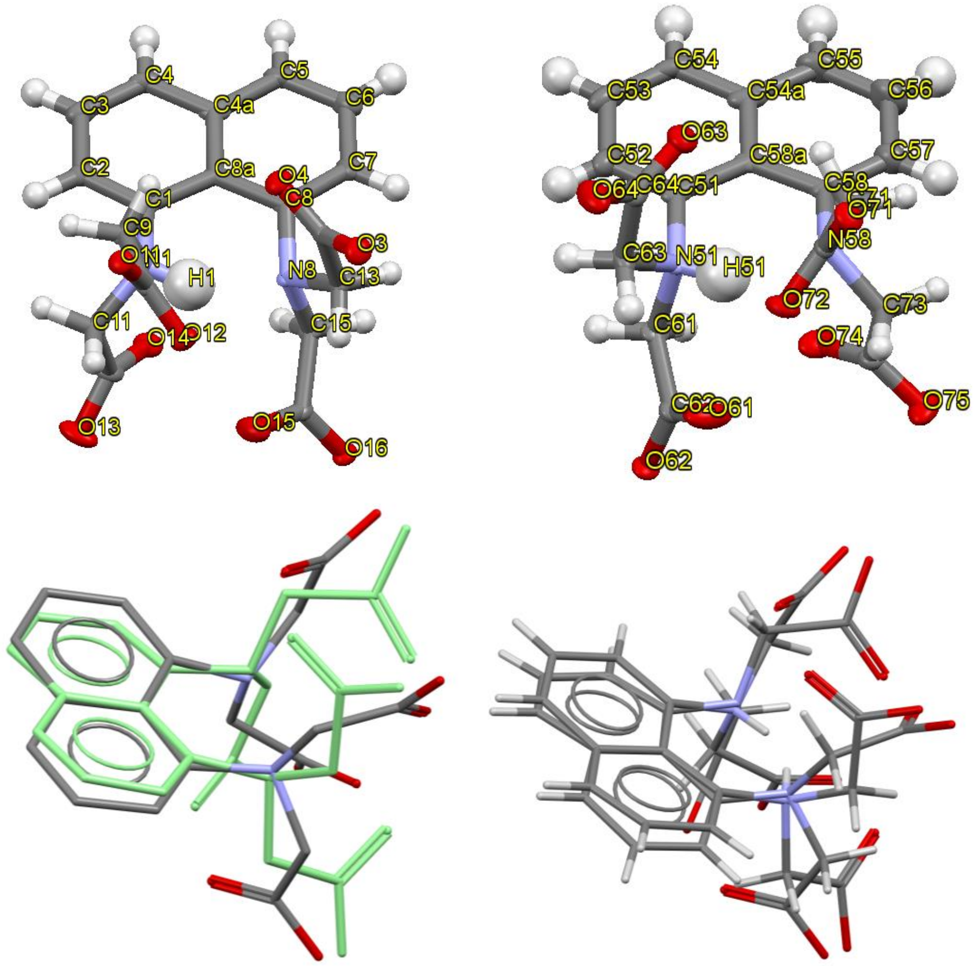

Figure S36. ORTEP view of the mono-protonated $HDANTA^{3-}$ (δ and λ isomer) at 50% probability level with numbering scheme (top). Solvent water and ethanol molecules as well as sodium counter ions are omitted for clarity. Overlay of the two molecules of the asymmetric unit in two orientations (bottom).

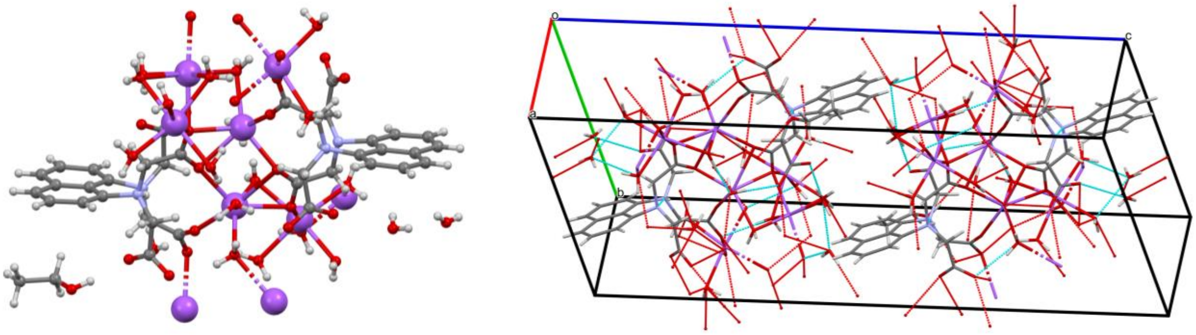

Figure S37. The asymmetric unit of $[Na_3(HDANTA)]\cdot EtOH\cdot 20H_2O$ (left) and its packing diagram (right) with the hydrogen bond network.

Table S5. Selected bond distances (Å) in [Na_3(HDANTA)]·EtOH·20H_2O

**δ isomer**		**λ isomer**	
C16—O15	1.239 (6)	C72—O72	1.242 (5)
C16—O16	1.265 (5)	C72—O71	1.259 (5)
C14—O4	1.246 (6)	C62—O61	1.237 (6)
C14—O3	1.250 (6)	C62—O62	1.260 (5)
C12—O14	1.229 (6)	C64—O63	1.239 (6)
C12—O13	1.274 (6)	C64—O64	1.256 (7)
C10—O12	1.249 (5)	C74—O74	1.217 (6)
C10—O11	1.262 (5)	C74—O75	1.265 (6)
**EtOH**			
C91—O91	1.430 (8)		

Table S6. Hydrogen bond parameters (Å, º) for [Na_3(HDANTA)]·EtOH·20H_2O. The numbering of the atoms in the δ and λ isomer starts from 1 and 51, respectively, as shown in Figure S11.

D—H···*A*	*D*—H	H···*A*	*D*···*A*	*D*—H···*A*
C2—H2···O3^{v}	0.95	2.40	3.348 (5)	176
C9—H9*B*···O4	0.99	2.38	2.991 (5)	119
C11—H11*A*···O3^{v}	0.99	2.50	3.478 (5)	170
C11—H11*B*···O12	0.99	2.51	3.105 (5)	118
C15—H15*B*···O14	0.99	2.48	3.139 (5)	124
C61—H61*A*···O74	0.99	2.34	3.024 (6)	125
C61—H61*B*···O13*W*	0.99	2.24	3.210 (5)	165
C63—H63*A*···O61	0.99	2.44	3.065 (5)	120
C71—H71*A*···O63	0.99	2.44	3.080 (5)	122
C71—H71*B*···O9*W*	0.99	2.63	3.552 (5)	155
C73—H73*A*···O72	0.99	2.64	3.200 (5)	116
C73—H73*B*···O64^{vi}	0.99	2.36	3.346 (5)	171
N1—H1···N8	0.87 (2)	1.84 (4)	2.645 (4)	153 (8)
O1*W*—H1*W*···O16	0.85 (1)	1.92 (2)	2.744 (4)	163 (6)

D—H···*A*	*D*—H	H···*A*	*D*···*A*	*D*—H···*A*
O10*W*—H10*A*···O15	0.87 (2)	2.29 (4)	3.077 (5)	150 (6)
O10*W*—H10*A*···O16	0.87 (2)	2.35 (4)	3.144 (4)	152 (7)
O10*W*—H10*B*···O62^{i}	0.87 (2)	1.97 (3)	2.806 (4)	162 (6)
O11*W*—H11*C*···O17*W*	0.85 (1)	1.94 (2)	2.785 (5)	174 (8)
O11*W*—H11*D*···O13^{vi}	0.85 (1)	2.11 (2)	2.949 (5)	169 (7)
O12*W*—H12*A*···O71^{iii}	0.86 (2)	1.99 (4)	2.782 (4)	153 (7)
O12*W*—H12*B*···O63^{iii}	0.86 (2)	2.11 (4)	2.914 (4)	158 (7)
O13*W*—H13*C*···O74^{v}	0.85 (2)	2.54 (2)	3.386 (5)	173 (7)
O13*W*—H13*C*···O75^{v}	0.85 (2)	2.42 (6)	3.055 (5)	132 (7)
O13*W*—H13*D*···O20*W*^{iv}	0.84 (2)	2.03 (4)	2.815 (5)	154 (7)
O14*W*—H14*A*···O16^{iv}	0.85 (2)	2.08 (2)	2.924 (4)	175 (7)
O14*W*—H14*B*···O13^{iii}	0.84 (2)	1.98 (5)	2.678 (4)	139 (7)
O15*W*—H15*C*···O1*W*^{iv}	0.83 (2)	1.99 (3)	2.790 (4)	162 (7)
O15*W*—H15*D*···O3^{v}	0.85 (2)	1.89 (4)	2.684 (4)	156 (7)
O16*W*—H16*A*···O18*W*	0.88 (2)	1.90 (2)	2.777 (6)	175 (8)
O17*W*—H17*A*···O12	0.84 (2)	2.60 (6)	3.271 (4)	137 (7)
O17*W*—H17*B*···O3	0.85 (2)	1.90 (2)	2.752 (4)	175 (8)
O18*W*—H18*A*···O17*W*^{v}	1.19 (4)	2.50 (4)	3.668 (6)	165 (6)
O18*W*—H18*B*···O64	0.90 (2)	1.81 (4)	2.658 (6)	156 (9)
O20*W*—H20*A*···O21*W*	0.91 (2)	1.75 (4)	2.618 (11)	158 (7)
O20*W*—H20*B*···O9*W*	0.87 (2)	2.33 (8)	2.791 (5)	113 (6)
O20*W*—H20*B*···O13*W*^{ii}	0.87 (2)	2.38 (7)	2.815 (5)	111 (6)
N51—H51···N58	0.87 (2)	1.85 (5)	2.619 (4)	146 (8)
O91—H91···O14	0.85 (2)	1.92 (3)	2.760 (5)	169 (8)

D—H···A	D—H	H···A	D···A	D—H···A
O1W—H1AW···O91^{vi}	0.85 (1)	1.94 (1)	2.782 (4)	171 (5)
O2W—H2W1···O10W^{iii}	0.84 (2)	2.30 (4)	3.085 (5)	155 (7)
O2W—H2W2···O14W^{vi}	0.84 (2)	2.63 (3)	3.127 (4)	119 (3)
O2W—H2W2···O15W^{vi}	0.84 (2)	2.37 (4)	3.061 (5)	140 (3)
O2W—H2W2···O16W^{vi}	0.84 (2)	2.63 (2)	3.251 (6)	132 (2)
O3W—H3W1···O13^{iii}	0.85 (2)	2.56 (5)	3.256 (5)	141 (7)
O3W—H3W2···O62	0.85 (2)	1.98 (2)	2.822 (4)	172 (8)
O4W—H4W1···O14^{iii}	0.85 (2)	2.05 (3)	2.873 (4)	164 (7)
O4W—H4W2···O5W	0.86 (2)	2.39 (7)	2.932 (4)	121 (6)
O5W—H5W1···O1W^{iii}	0.85 (2)	2.01 (4)	2.811 (4)	156 (7)
O5W—H5W2···O4	0.85 (2)	1.85 (2)	2.698 (4)	171 (8)
O6W—H6W1···O13	0.83 (2)	2.05 (3)	2.843 (5)	159 (7)
O6W—H6W2···O18W	0.84 (2)	1.99 (4)	2.760 (5)	152 (7)
O7W—H7W1···O75^{i}	0.85 (2)	1.89 (2)	2.737 (4)	172 (8)
O7W—H7W2···O14W^{ii}	0.84 (2)	2.01 (2)	2.852 (4)	174 (7)
O8W—H8W1···O12W^{i}	0.85 (2)	2.11 (3)	2.912 (4)	157 (7)
O8W—H8W2···O74^{i}	0.86 (2)	1.91 (3)	2.749 (4)	164 (7)
O9W—H9W1···O64^{vi}	0.84 (2)	1.89 (3)	2.700 (4)	161 (7)
O9W—H9W2···O20W	0.84 (2)	1.97 (3)	2.791 (5)	163 (9)

Symmetry codes: (i) $x, y+1, z$; (ii) $x-1, y+1, z$; (iii) $x, y-1, z$; (iv) $x+1, y-1, z$; (v) $x+1, y, z$; (vi) $x-1, y, z$.

A survey of the Cambridge Structural Database[30] reveals that the average difference of the absolute value of C-O distances in carboxylate ions is 0.026(30) Å (20000+ hits). This agrees with our corresponding data of 0.025(10) Å. The average difference of the absolute value of C-O distances in carboxylic acids is 0.09(3) Å. The average distance of the protonated nitrogen from the plane of the naphthalene ring is 0.07(6) Å (193 hits) while the same average distance

when none of the nitrogens are protonated is 0.35(15) Å (36 hits). The N-N distance for deprotonated and protonated derivatives are 2.93(12) Å and 2.59(5) Å, respectively

7. In vitro and in vivo MRI investigation of $HDANTA^{3-}$

7.1. CEST NMR spectroscopy

CEST-spectra were recorded on an 11.7 T (600MHz) or a 9.4 T (400 MHz) Varian/Agilent NMR spectrometer. The CEST experiment was performed by measuring the bulk water proton signal intensity after an optimized saturation pulse (B_1) (2.8 μT) for a specific period of time (t_{sat} = 3 s), with a repetition time between scans of 10 s and acquisition time of 1 s. The saturation frequency was arrayed at regular intervals of frequency in the range of 12000 Hz (20 ppm) and in steps of 150 Hz. The Z/CEST spectrum was obtained by plotting the percent decrease of intensity of the water signal (S_{sat}/S_0) versus the saturation frequency (ppm) over the entire range of frequency offsets as shown in Figure S38.

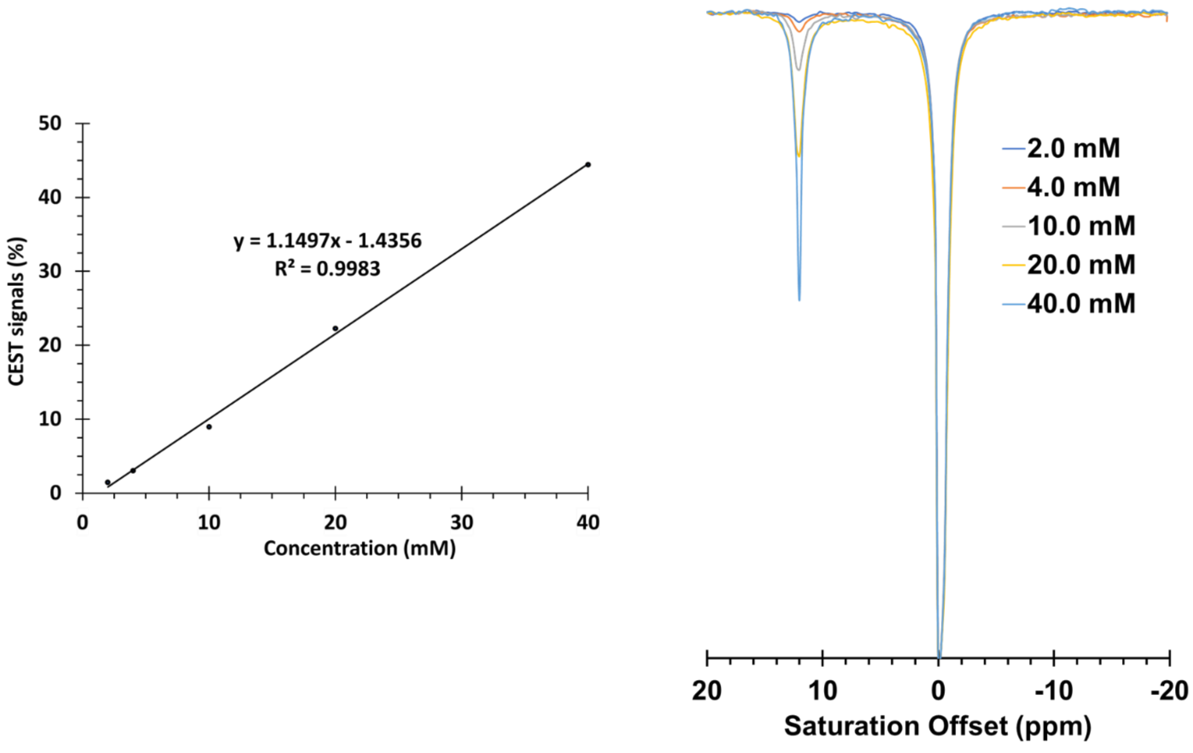

Figure S38. The dependence of the CEST effect on the concentration of DANTA at pH 7, B_1 = 2.8 μT, 37 °C, 14.1 T.

7.2. CEST spectroscopy experiments in the presence of Ca^{2+}

CEST experiments were performed in 20 mM DANTA solutions containing equimolar $CaCl_2$ at 37 °C in the pH range of 6 to 9.

7.3. Phantom imaging

DANTA and salicylic acid solutions were dissolved in 0.01 M phosphate-buffered saline (PBS) at 10, 20, 40 mM concentrations, and titrated using 1M HCl/NaOH to pH 7. The solutions were placed into glass tubes (200 µL, 4.8 × 31 mm, flat bottom vial inserts) and sealed with glue for CEST MR imaging. The samples were kept at 37°C during imaging. Phantom CEST experiments were taken on a Bruker 3 T MR scanner, using a 20 mm birdcage transmit/receive coil. CEST images were acquired using a RARE sequence[31] with CW saturation pulse length of 3 s and saturation field strength (B_1) of 1.0 µT, 3.0 µT, 5.0 µT. The CEST (Z) spectra were acquired by incrementing saturation frequency every 0.25 ppm from −20 to 20 ppm; TR = 5500 ms, effective TE = 4 ms, RARE factor = 64, matrix size = 128 × 64 and slice thickness = 2 mm.

7.4. In vivo CEST MRI

All procedures described in this study were approved by the UT Southwestern Institutional Animal Care and Use Committee (IACUC Protocol Number: 2022-103224). In vivo images were acquired on a Bruker BioSpec 3 T MR scanner, with one axial slice of 1.5 mm thickness obtained through the medulla of both kidneys. CEST images were acquired using a RARE sequence with CW saturation pulse length of 3 s and optimized saturation field strength (B_1) of 3 µT. After shimming on the water signal, a standard T_2 weighted ^{1}H image were acquired to position the axial slice plane through both kidneys. The CEST-spectra were acquired by incrementing saturation frequency every 0.25 ppm from −20 to 20 ppm; TR = 5240 ms, effective TE = 6.7 ms, RARE factor = 32, matrix size = 196 × 96 and slice thickness = 2 mm. BALB/c mice (n = 3) weighing 22-27 g (Charles River Laboratories, Wilmington, MA) were anesthetized by using 0.5–2% isoflurane and placed in a 23 mm transmit/receive mouse coil. Breath rate was monitored throughout in vivo MRI experiments using a respiratory probe. DANTA (dose: approximately 1 mmol/kg DANTA plus 0.33 mmol/kg $CaCl_2$, 200 µL solution containing 150 mM DANTA and 50 mM $CaCl_2$ in PBS buffer pH = 7) was slowly injected via a catheter into the tail vein. Custom developed in-house Matlab was used to construct the CEST images and quantify the CEST contrast.

8. References

(1) Sherry, A. D.; Castelli, D. D.; Aime, S. Prospects and Limitations of Paramagnetic Chemical Exchange Saturation Transfer Agents Serving as Biological Reporters in Vivo. *NMR in Biomed.* **2023**, *36* (6), e4698. https://doi.org/10.1002/nbm.4698.

(2) Yuan, Y.; Zhang, J.; Qi, X.; Li, S.; Liu, G.; Siddhanta, S.; Barman, I.; Song, X.; McMahon, M. T.; Bulte, J. W. M. Furin-Mediated Intracellular Self-Assembly of Olsalazine Nanoparticles for Enhanced Magnetic Resonance Imaging and Tumour Therapy. *Nat. Mater.* **2019**, *18* (12), 1376–1383. https://doi.org/10.1038/s41563-019-0503-4.

(3) Yang, X.; Song, X.; Li, Y.; Liu, G.; Ray Banerjee, S.; Pomper, M. G.; McMahon, M. T. Salicylic Acid and Analogues as diaCEST MRI Contrast Agents with Highly Shifted Exchangeable Proton Frequencies. *Angew. Chem. Int. Ed.* **2013**, *52* (31), 8116–8119. https://doi.org/10.1002/anie.201302764.

(4) Yang, X.; Yadav, N. N.; Song, X.; Ray Banerjee, S.; Edelman, H.; Minn, I.; van Zijl, P. C. M.; Pomper, M. G.; McMahon, M. T. Tuning Phenols with Intra-Molecular Bond Shifted HYdrogens (IM-SHY) as diaCEST MRI Contrast Agents. *Chem. – A Eur. J.* **2014**, *20* (48), 15824–15832. https://doi.org/10.1002/chem.201403943.

(5) Yang, X.; Song, X.; Ray Banerjee, S.; Li, Y.; Byun, Y.; Liu, G.; Bhujwalla, Z. M.; Pomper, M. G.; McMahon, M. T. Developing Imidazoles as CEST MRI pH Sensors. *Cont. Med. & Mol. Imag.* **2016**, *11* (4), 304–312. https://doi.org/10.1002/cmmi.1693.

(6) Aime, S.; Calabi, L.; Biondi, L.; De Miranda, M.; Ghelli, S.; Paleari, L.; Rebaudengo, C.; Terreno, E. Iopamidol: Exploring the Potential Use of a Well-Established x-Ray Contrast Agent for MRI. *Magn. Reson. Med.* **2005**, *53* (4), 830–834. https://doi.org/10.1002/mrm.20441.

(7) *Pilot study of Iopamidol-based quantitative pH imaging on a clinical 3T MR scanner | Magnetic Resonance Materials in Physics, Biology and Medicine | Springer Nature Link.* https://link.springer.com/article/10.1007/s10334-014-0433-8 (accessed 2026-03-23).

(8) Longo, D. L.; Dastrù, W.; Digilio, G.; Keupp, J.; Langereis, S.; Lanzardo, S.; Prestigio, S.; Steinbach, O.; Terreno, E.; Uggeri, F.; Aime, S. Iopamidol as a Responsive MRI-Chemical Exchange Saturation Transfer Contrast Agent for pH Mapping of Kidneys: In Vivo Studies in Mice at 7 T. *Magn. Reson. Med.* **2011**, *65* (1), 202–211. https://doi.org/10.1002/mrm.22608.

(9) Ward, K. M.; Aletras, A. H.; Balaban, R. S. A New Class of Contrast Agents for MRI Based on Proton Chemical Exchange Dependent Saturation Transfer (CEST). *J. Magn. Reson.* **2000**, *143* (1), 79–87. https://doi.org/10.1006/jmre.1999.1956.

(10) McMahon, M. T.; Gilad, A. A.; Zhou, J.; Sun, P. Z.; Bulte, J. W. M.; van Zijl, P. C. M. Quantifying Exchange Rates in Chemical Exchange Saturation Transfer Agents Using the Saturation Time and Saturation Power Dependencies of the Magnetization Transfer Effect on the Magnetic Resonance Imaging Signal (QUEST and QUESP): Ph Calibration for Poly-L-Lysine and a Starburst Dendrimer. *Magn. Reson. Med.* **2006**, *55* (4), 836–847. https://doi.org/10.1002/mrm.20818.

(11) Chan, K. W. Y.; Yu, T.; Qiao, Y.; Liu, Q.; Yang, M.; Patel, H.; Liu, G.; Kinzler, K. W.; Vogelstein, B.; Bulte, J. W. M.; van Zijl, P. C. M.; Hanes, J.; Zhou, S.; McMahon, M. T. A diaCEST MRI Approach for Monitoring Liposomal Accumulation in Tumors. *J. Contr. Release* **2014**, *180*, 51–59. https://doi.org/10.1016/j.jconrel.2014.02.005.

(12) Zhang, X.; Yuan, Y.; Li, S.; Zeng, Q.; Guo, Q.; Liu, N.; Yang, M.; Yang, Y.; Liu, M.; McMahon, M. T.; Zhou, X. Free-Base Porphyrins as CEST MRI Contrast Agents with Highly Upfield Shifted Labile Protons. *Magn. Reson. Med.* **2019**, *82* (2), 577–585. https://doi.org/10.1002/mrm.27753.

(13) Irving, H. M.; Miles, M. G.; Pettit, L. D. A Study of Some Problems in Determining the Stoicheiometric Proton Dissociation Constants of Complexes by Potentiometric Titrations

Using a Glass Electrode. *Anal. Chim. Acta* **1967**, *38*, 475–488. https://doi.org/10.1016/S0003-2670(01)80616-4.

(14) L. Zékány; I. Nagypál. PSEQUAD. In *Computational Methods for the Determination of Formation Constants*; D. J. Leget, Ed.; Plenum Press: New York, 1985; pp 291–353.

(15) Submeier, J. L.; Reilley, C. N. Nuclear Magnetic Resonance Studies of Protonation of Polyamine and Aminocarboxylate Compounds in Aqueous Solution. *Anal. Chem.* **1964**, *36* (9), 1698–1706. https://doi.org/10.1021/ac60215a006.

(16) Beck, M. T.; Nagypal, I. *Chemistry of Complex Equilibria*; Horwood, 1990.

(17) Forgacs, A.; Giovenzana, G. B.; Botta, M.; Brucher, E.; Toth, I.; Baranyai, Z. Influence of Gem-Dimethyl Substitution on the Stability, Kinetics and Relaxometric Properties of PDTA Complexes. *Eur. J. Inorg. Chem.* **2012**, No. 12, 2074–2086. https://doi.org/10.1002/ejic.201101294.

(18) Anderegg, G. Komplexone XXXVI. Reakinsenthalpie Und -Entropie Bei Der Bildung Der Metallkomplexe Der Höheren EDTA-Homologen. *Helv. Chim. Acta* **1964**, *47* (7), 1801–1814. https://doi.org/10.1002/hlca.19640470716.

(19) Anderegg, G.; Wenk, F. Komplexone VL. Reaktionsenthalpie Und -Entropie Bei Der Bildung Der 1 : 1-Komplexe Der Seltenen Erdionen Mit 1,3-Diaminopropan-N, N, N′, N′-Tetraacetat. *Helv. Chim. Acta* **1971**, *54* (1), 216–229. https://doi.org/10.1002/hlca.19710540119.

(20) Hibbert, F. Temperature-Jump Study of Proton Transfer from Protonated 1,8-Bis-(Dialkylamino)Naphthalenes to Hydroxide Ion in Water and Aqueous Dioxan. *J. Chem. Soc., Perkin Trans. 2* **1974**, No. 15, 1862–1866. https://doi.org/10.1039/P29740001862.

(21) Morris, G. A.; Freeman, R. Selective Excitation in Fourier Transform Nuclear Magnetic Resonance. *J. Magn. Reson. (1969)* **1978**, *29* (3), 433–462. https://doi.org/10.1016/0022-2364(78)90003-3.

(22) Sheldrick, G. M. Crystal Structure Refinement with SHELXL. *Acta Crystal. C Struct. Chem.* **2015**, *71* (Pt 1), 3–8. https://doi.org/10.1107/S2053229614024218.

(23) Westrip, S. P. publCIF: Software for Editing, Validating and Formatting Crystallographic Information Files. *J. Appl. Cryst.* **2010**, *43* (4), 920–925. https://doi.org/10.1107/S0021889810022120.

(24) Spek, A. L. PLATON, A Multipurpose Crystallographic Tool, 1998.

(25) Spek, A. PLATON, An Integrated Tool for the Analysis of the Results of a Single Crystal Structure Determination. *Acta Crystal. Section A*, 1990, *46*, c34.

(26) Allen, F. H.; Watson, D. G.; Brammer, L.; Orpen, A. G.; Taylor, R. Typical Interatomic Distances: Organic Compounds. In *International Tables for Crystallography*; John Wiley & Sons, Ltd, 2006; pp 790–811. https://doi.org/10.1107/97809553602060000621.

(27) Farrugia, L. J. WinGX and ORTEP for Windows: An Update. *J. Appl. Cryst.* **2012**, *45* (4), 849–854. https://doi.org/10.1107/S0021889812029111.

(28) Macrae, C. F.; Sovago, I.; Cottrell, S. J.; Galek, P. T. A.; McCabe, P.; Pidcock, E.; Platings, M.; Shields, G. P.; Stevens, J. S.; Towler, M.; Wood, P. A. Mercury 4.0: From Visualization to Analysis, Design and Prediction. *J. Appl. Cryst.* **2020**, *53* (1), 226–235. https://doi.org/10.1107/S1600576719014092.

(29) Schrodinger, L. The PyMOL Molecular Graphics System, 2015. http://www.pymol.org.

(30) Groom, C. R.; Bruno, I. J.; Lightfoot, M. P.; Ward, S. C. The Cambridge Structural Database. *Acta Cryst. B* **2016**, *72* (2), 171–179. https://doi.org/10.1107/S2052520616003954.

(31) Hennig, J.; Nauerth, A.; Friedburg, H. RARE Imaging: A Fast Imaging Method for Clinical MR. *Magn. Reson. Med.* **1986**, *3* (6), 823–833. https://doi.org/10.1002/mrm.1910030602.
